# A genus in disguise. Revision of the genus *Salcedia* Fairmaire, 1899 with descriptions of nine new species (Coleoptera, Carabidae, Scaritinae, Salcediini)

**DOI:** 10.3897/zookeys.901.39432

**Published:** 2020-01-09

**Authors:** Michael Balkenohl

**Affiliations:** 1 Naturhistorisches Museum Bern, Abt. Wirbellose Tiere, Bernastrasse 15, CH-3005, Bern, Switzerland Naturhistorisches Museum Bern Bern Switzerland

**Keywords:** Africa, Madagascar, Oriental region, zoogeography, biology, key to species

## Abstract

This monograph on the genus *Salcedia* Fairmaire, 1899 revises the nine described species *S. perrieri* Fairmaire, 1899, *S. coquilhati* Alluaud, 1932, *S. elongata* Alluaud, 1932, *S. africana* (Britton, 1947), *S. schoutedeni* Alluaud, 1930, *S. putzeysi* (Oberthür, 1883), *S. miranda* (Andrewes, 1920), and *S. parallela* Baehr, 1998. The following nine new species are described: *S. unifoveata***sp. nov.**, *S. faillei***sp. nov.**, *S. lukulua***sp. nov.**, *S. matsumotoi***sp. nov.**, *S. utetea***sp. nov.**, *S. robusta***sp. nov.**, *S. procera***sp. nov.**, *S. tuberculata***sp. nov.**, and *S. baroensis***sp. nov.** Photographs of the habitus and male and female genitals are provided for all species. An identification key to the species is given. Morphological characteristics of the genus are described and illustrated. Zoogeography of the group is discussed and distribution records from Africa are displayed on a map. Available biological data are summarised.

## Introduction

The genus *Salcedia* Fairmaire, 1899 comprises small grey to brownish species of approximately 2.4–4 mm length. At first glance, the species, which are generally coated with dirt, appear very homogenous, but on detailed examination they exhibit distinct species-specific characters.

The genus is closely related to two South American genera, *Holoprizus* Putzeys, 1866 and *Solenogenys* Westwood, 1859. In current catalogues the genus *Andozelma* Dostal, 1993, is placed as well in the tribe Salcediini. However, this placement is questioned by the author (compare discussion).

At the suprageneric level, the three genera seem to be isolated. According to Basilewsky (1973), Kryzhanovskiy (1976), Lorenz (2005), Bouchard et al. (2011), and Hogan (2012) they form their own tribe Salcediini among the Scaritinae. Reichardt (1975) treated the group as a subtribe, and Erwin and Sims (1984) and Erwin (1985) made it evident that the group belongs in the tribe Clivinini. He also reported studies that showed that members of Salcediina constitute the sister group of *Stratiotes* Putzeys, and thus the two subtribes Forcipatorina and Salcediina will be merged (Erwin and Sims 1984: 371, 373). Bousquet (2012) summarised results from Bell (1998) who included *Androzelma* and formed the tribe Salcediini. According to those results, salcediines share synapomorphies with rhysodids, and Bell (1998) concluded that the genus *Solenogenys* is the sister group of Rhysodidae.

*Holoprizus* is monotypic and three species of *Solenogenys* are known. Both of the genera occur on the banks of the Rio Amazonas, Rio Negro and its tributaries (Adis 1981). They were found as single specimens or in very small series. *Salcedia* is the most species rich genus, and radiated according to current knowledge to eighteen species occurring over wide areas of tropical Africa and the Oriental region.

Alluaud (1930) synonymised *Zelma* Andrewes, 1929 with *Salcedia*. *Holoprizus putzeysi* Oberthür, 1883 was transferred into *Salcedia* by Andrewes (1936). Reichardt (1975) transferred *Zelma africana* Britton, 1947 to *Salcedia*. Reichardt revised the group on the suprageneric level, provided a key to the three genera, and revised the species of *Holoprizus* and *Solenogenys* (Reichardt 1975, 1977). For *Solenogenys* a description of a new species including an update of the key to the species was provided by Adis (1981). However, for the genus *Salcedia* there has never been a complete account or a revision at the species level. A short key to three species given by Alluaud (1930) is based on mud-coated specimens. With the exception of three species (*S. miranda* (Andrewes, 1920), *S. africana* (Britton, 1947), *S. parallela* Baehr, 1998), the descriptions of the other described species are short. Most of the distinct and distinguishing characters are not mentioned by authors. The reason might be the special appearance of the specimens, which are in general covered with mud or clay like obscuring characters, making observations difficult and making the species looking strikingly similar at first glance. Therefore, authors sometimes took ratios of measurements as one of the main distinguishing characters. However, labour-intensive cleaning of the small species (see methods) demonstrated many characters were never described before. This is also a reason redescriptions of all known species were needed and are provided in this contribution.

To date, seven species from Africa and two species from the Oriental region have been described. In an effort to determine unidentified material it became apparent that the material cannot be determined with confidence without revising the described species. Investigation of the type material and determined specimens of *Salcedia* deposited in the Musée Royal de l´Afrique Centrale (MRACT) revealed that the material was confused and contains undescribed species.

In addition to the Museums in Tervuren (MRACT) and Paris (MNHN), members of the genus were only located in few other collections, mostly represented by a few specimens if any. The reason for their seemingly rarity might be that they are not recognised as carabids, because at first glance they look like Colydiidae (e.g., genus *Dastarcus* Walker, 1858) or small Tenebrionidae (e.g., genus *Gonocephalum* Chevrolat, 1849). Another reason might be they are often simply overlooked due to their hidden way of life. Many specimens so far examined were collected using light traps used along rivers and shores of lakes over wide parts of tropical Africa and Madagascar. A small series from Madagascar was made available from soil washings. From a few localities, only single specimens were found. To date, the Oriental taxa are represented by only five specimens.

This contribution aims to revise the genus *Salcedia* at the species level by considering all of the available material and information.

## Materials and methods

### Material examined

Holotypes and other available type material of the redescribed species were investigated. In addition, some other material was located and it was possible to base this monograph on a total of 652 specimens.

The complete information given on the labels is displayed in the descriptions of the species, verbatim as they appear on the labels including spelling, spacing, and punctuation. Many of the labels give historic names of former countries. In these cases, the names in use today are given in addition, under ‘distribution’ in the descriptions.

For comparison of several characters the following other species have been investigated: *Solenogenys foeda* Westwood, 1858, *S. thomsoni* Reichardt, 1975, *S. funkei* Adis, 1981, *Holoprizus serratus* Putzeys, 1866 (BMNH and MNHN).

### Terminology

**Figure 1.**
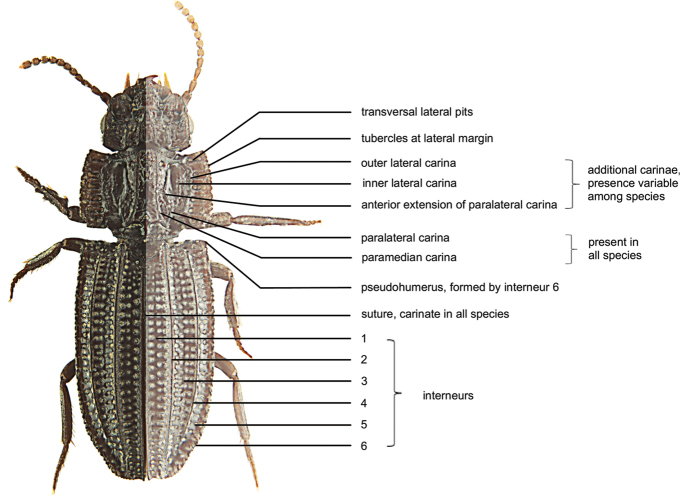
Semi-schematic dorsal view of *Salcedia perrieri* Fairmaire highlighting some of the features and discriminating characters used in the descriptions and the identification key. Interneurs 7 and 8 visible in ventral view (see Fig. 2).

**Figure 2.**
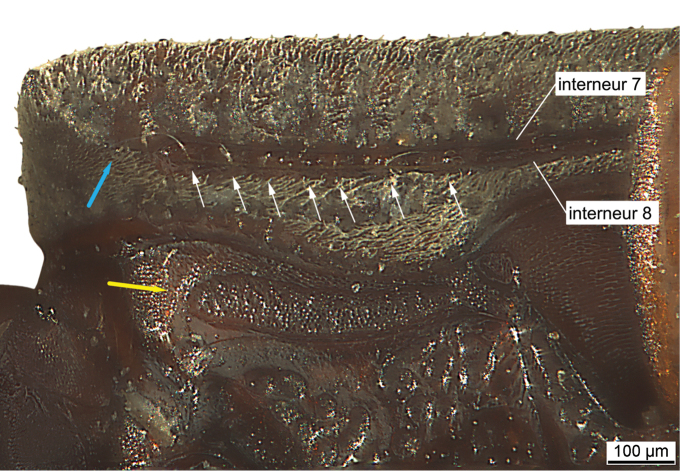
*Salcedia baroensis* sp. nov., latero-ventral view, basal half of elytron and metepisternum. White arrows: series of umbilical setae arising from tuberculate punctures. Blue arrow: real humerus, yellow arrow: longitudinal groove for the reception of the apical half of the intermediate tibia and the tarsomeres.

In general terms, descriptions of characters and methods were based on Balkenohl (2001). Special terms are explained in Figs 1, 2.

Describing the surface of the elytra is more complex than in other Clivinini. On the one hand one can describe what one sees. This results in many of the *Salcedia* species in three distinctly raised longitudinal carinae but also in 14 rows of punctures. It is obvious this cannot be intervals and striae even if it looks like it superficially and at first glance. The reason for the complexity is the lack of a precise term for the actual structures, e.g., impressed unconnected punctures arranged in longitudinal rows with or without connection, and others. This issue has already been recognised (Spilman 1971), but was also resolved by a slightly changed terminology proposed by Erwin (1974: 3–5) and based on a phylogenetic description technique. Consequently, that terminology is used. It was also applied by Adis (1981) when he revised *Solenogenys*. In *Salcedia*, and according to this terminology, six interneurs (formerly also called striae) are present on the dorsal surface (Fig. 1) and two latero-ventrally (Fig. 2). The impressed rows of circular pits are intervals. In most of the species, the impressed rows are partly or completely doubled (Figs 1, 44–51). At the base of the elytron, interneur six forms a kind of humerus. Usually the humerus is formed by interneur eight at the basal end of the lateral channel. Often the lateral channel and interneur eight bend round the humerus up to the base. In *Salcedia* the lateral channel and interneur eight already ends before reaching the base. That point is recognised as the real humerus. It is continued as carina-like line with an obtuse indistinct angle up to the dorsally visible structure formed by interneur six which is called the pseudohumerus. For the count of the tubercles at the lateral margin of the pronotum, the anterior and posterior angles are not counted.

The lateral ventral carina of the front tibia often shows a different degree of digitation. The terminal upper spine, as well as an obtuse spine that can sometimes be present at the base of the tibia, is not counted in the descriptions.

The following terms and their explanations describing the colour and surface were taken from Andrewes (1929) and Csiki (1946): coriacious – with a surface like that of leather; reticulate – covered with a network of very fine striae, which form meshes of various shape; fuscous – very dark brown, almost black; griseous – grey, almost medium grey; hinnuleous – brown like a deer; leoninous – yellowish brown coloured like a lion; melaneous – completely black.

Terms used for the male genitalia refer to the natural position in non-dissected specimens. For the description of the female gonocoxites, terminology follows Deuve (1993).

### Microscopy and measurements

Specimens were examined using a Leica M205-C stereomicroscope and a Reichert-Jung Polyvar compound microscope. Measurements and angular degrees were taken electronically using the integrated and automatically calibrating measurement system of the Imagic Client software. The respective body parts of the specimens (head, pronotum, elytron) must to be placed in an exactly horizontal position. If available, ten specimens of each species were measured including the holotype. Body length was measured from the apex of the longer mandible in closed position to the apex of the elytra. The length of the pronotum was measured along the median line including the posteriorly produced base, and the width was determined at the widest part. The length of the elytron was measured from the anterior basal tip of the lateral margin to the tip of the apex. The elytral width was measured at the maximum width of both elytra in closed position and represents the general the width of the specimen. The limits of the measurements and arithmetic means (X̄) are provided for the values of all species including ratios calculated (Table 1). For the description of the antennae and for the length of the antennomeres five to ten it was necessary to expand and refine the commonly used terms and definitions Kult (1959) used for Clivinini. So, the length/width (L/W) ratios of antennomere six is given in addition and summarised in Table 2. These measurements were taken for both sides of a specimen, and if available for up to three specimens.

**Table 1.** Measurements and ratios of *Salcedia* species. *N* = number of specimens measured; x̄ = arithmetic mean; “term used” refers to the shape of the elytra.

**Region**	**Species**	**Body length [mm**]		**Width [mm**]		**Length of elytron [mm**]		**Ratio length/width of pronotum**		**Ratio length/width of elytra**			**Posterior lateral angle of head**	**N**
		**Range**	**x**¯	**Range**	**x**¯	**Range**	**x**¯	**Range**	**x**¯	**Range**	**x**¯	**Term used**		
Madagascar	*S. perrieri* Fairmaire, 1899	3.22–3.59	3.47	1.1–1.22	1.18	1.95–2.19	2.09	0.65–0.71	0.69	1.71–1.82	1.78	subelongate	obtuse, ~125°	10
	*S. unifoveata* sp. nov.	2.6–3.11	2.85	0.91–1.13	1.03	1.48–1.81	1.65	0.74–0.75	0.75	1.54–1.63	1.59	long-ovoid	obtuse, ~118°	6
	*S. faillei* sp. nov.	3.06	–	0.98	–	1.83	–	0.73	–	1.89	–	elongate	128°	1
Continental Africa	*S. coquilhati* Alluaud, 1932	2.37–2.76	2.52	0.95–1.13	1.03	1.41–1.75	1.56	0.55–0.59	0.57	1.48–1.55	1.51	long-ovoid	reg. convex	10
	*S. elongata* Alluaud, 1932	3.3–3.65	3.49	1.04–1.17	1.13	2.07–2.27	2.17	0.67–0.76	0.72	1.83–2.03	1.92	oblongo-elongate	135–136°	10
	*S. africana* (Britton, 1947)	3.32–3.97	3.63	1.0–1.2	1.1	2.01–2.28	2.16	0.67–0.76	0.72	1.87–2.04	1.97	oblongo-elongate	118–119°	10
	*S. procera* sp. nov.	3.09–3.41	3.32	0.95–1.01	0.97	1.9–2.07	2.01	0.75–0.79	0.77	1.99–2.11	2.06	super-elongate	105–107°	8
	*S. schoudtedeni* Alluaud, 1930	3.39–3.76	3.54	1.35–1.45	1.37	2.03–2.38	2.21	0.58–0.61	0.6	1.58–1.65	1.62	subelongate	108–111°	10
	*S. nigeriensis* Alluaud, 1932	3.27–3.71	3.55	1.10–1.30	1.25	2.06–2.33	2.18	0.63–0.66	0.65	1.64–1.93	1.74	subelongate	96–98°	10
	*S. baroensis* sp. nov.	3.53–3.89	3.74	1.25–1.37	1.32	1.71–1.76	1.73	0.61–0.68	0.63	1.71–1.76	1.73	subelongate	~107°	10
	*S. utetea* sp. nov.	3.4–3.58	3.48	1.16–1.26	1.21	2.08–2.17	2.11	0.63–0.66	0.64	1.71–1.79	1.74	subelongate	96–99°	10
	*S. lukulua* sp. nov.	2.8; 2.84	–	0.89; 0.96	–	1.67; 1.72	–	0.73; 0.74	–	1.79; 1.88	–	elongate	obtuse, ~137°	2
	*S. robusta* sp. nov.	3.54–4.2	3.97	1.2–1.43	1.33	2.2–2.59	2.42	0.66–0.69	0.68	1.76–1.82	1.79	subelongate	115–117°	10
	*S. tuberculata* sp. nov.	3.43	–	1.03	–	1.99	–	0.74	–	1.94	–	oblongo-elongate	119°	1
	*S. putzeysi* (Oberthür, 1883)	3.12–3.88	3.52	1.24–1.28	0.25	2.11–2.33	1.17	0.63–0.65	0.35	1.67–1.81	0.74	subelongate	120–123°	10
	*S. matsumotoi* sp. nov.	3.02–3.59	3.36	0.94–1.19	1.1	1.82–2.24	2.08	0.72–0.81	0.75	1.84–1.96	1.9	elongate	~122°	10
Oriental region	*S. miranda* (Andrewes, 1920)	3.76; 3.96	–	1.35; 1.47	–	2.24; 2.39	–	0.64; 0.65	–	1.63; 166	–	subelongate	90°	2
	*S. parallela* Baehr, 1998	3.61	–	1.15	–	2.21	–	0.73	–	1.91	–	oblongo-elongate	102°	1

**Table 2.** Terms used for the description of the antennae of *Salcedia* species. Antennomere six is taken as reference for antennomeres five to ten.

**Term used**	**Description of term**	**Ratio length/width of antennomere six**	**Species**
sub-moniliform	slightly wider than long	0.95–1.00	*S. procera* sp. nov.
moniliform	as long as wide	1.00–1.04	*S. elongata* Alluaud, 1932
super-moniliform	slightly longer than wide	1.09–1.10	*S. africana* (Britton, 1947)
sub-elongate	longer than wide	1.14–1.20	*S. lukulua* sp. nov.
			*S. unifoveata* sp. nov.
			*S. tuberculata* sp. nov.
elongate	longer than 1.20×	1.24–1.34	*S. perrieri* Fairmaire, 1899
			*S. schoutedeni* Alluaud, 1930
			*S. nigeriensis* Alluaud, 1932
			*S. coquilhati* Alluaud, 1932
			*S. putzeysi* (Oberthür, 1883)
			*S. parallela* Baehr, 1998
			*S. matsumotoi* sp. nov.
			*S. robusta* sp. nov.
			*S. utetea* sp. nov.
			*S. faillei* sp. nov.
oblong-elongate	longer than 1.35×	1.38–1.44	*S. miranda* (Andrewes, 1920)
			*S. baroensis* sp. nov.

The genitalia dissected were mounted on transparent celon cards and embedded in polyvinylpyrrolidone. After clearing overnight, these cards were fixed on an object glass slide and used under the compound microscope. Descriptions were made of the genitals under transmitted light from beneath, or with top light (Reichert-Jung Polyvar compound microscope; magnification ×80–×500), or both light regimes. In cases where setae or trichia of the gonopod IX are rubbed off, the pores from which they emerge are clearly visible using interference contrast. Dissected specimens are indicated separately under material as males and females, respectively.

### Imaging conditions

Photographs were taken with a 5-megapixel Jenoptic core 5 digital camera, either through the Leica M205–C stereomicroscope using a motorised focussing drive and diffused light with Leica hood LED5000 HDI for single pictures stacking automatically, or for the Polyvar compound microscope using the drive manually. All pictures are composites, processed and optimised by using Imagic Client software and enhanced with CorelDRAW Graphics Suite X5.

### Cleaning of specimens

**Figures 3, 4.**
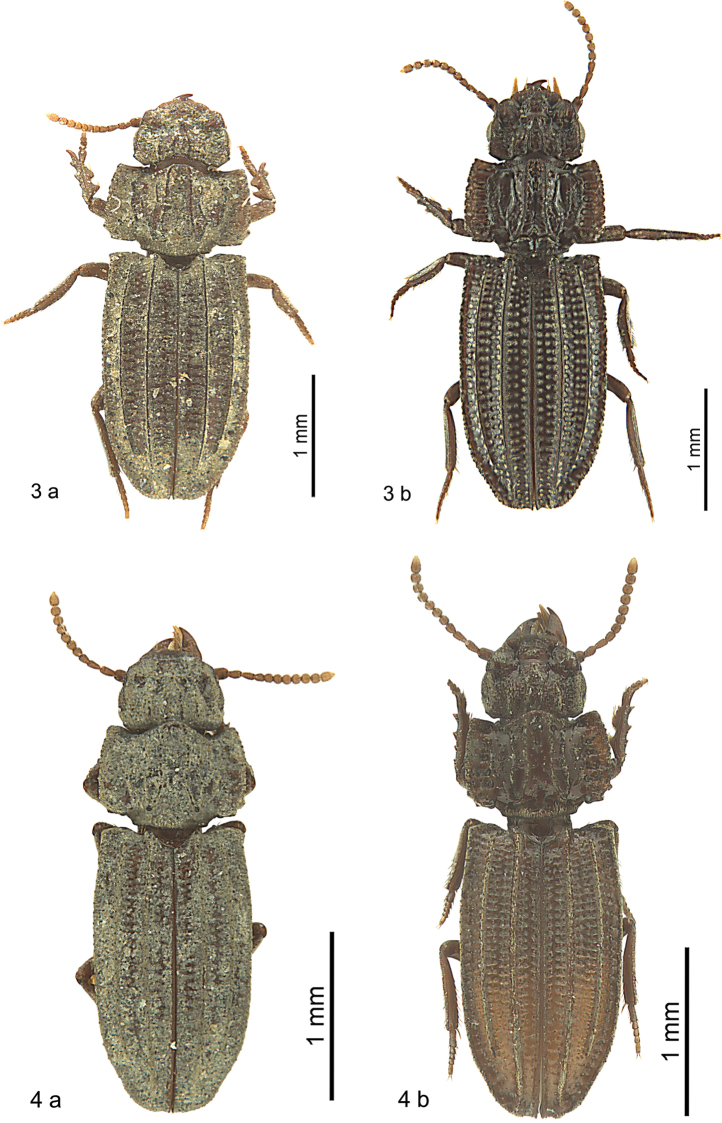
Identical *Salcedia* specimens in uncleaned (**a**) and cleaned (**b**) condition **3***S. perrieri* Fairmaire **4***S. lukulua* sp. nov.

The surface and especially the dorsal surface is usually coated with a tough film that obscures characters. Pits and grooves on the head, prothorax, and elytra are usually filled with dirt and substrate that adheres to the tough film. In most cases the pits and the space between the carinae of the prothorax and the interneurs of the elytra are mostly covered with mud or clay and in approximately half of the specimens the dorsal surface is completely clothed with substrate. However, the narrow lateral channel of the elytra and the series of umbilical setigerous punctures are always free of mud. Typical examples of two uncleaned species are provided to show exactly the same specimens in dirty and in clean condition (Figs 3, 4). Fig. 46 shows a partly cleaned elytron with the mud removed but the film coating still in place to a certain extent. In historic descriptions, this dirty covering obviously led to misinterpretation of characters. These layers cannot be removed mechanically without damaging the specimens, even when softened beforehand. The following softening solvents were not successful: water, ethanol in low and higher concentration, acetic acid, ethyl acetate, acetone or turpentine. The following cleaning method was applied and appeared to be successful: After slight boiling and soaking overnight in water containing an anionic tenside solution (5%), specimens were washed in a commercially available ultrasound bath also with anionic tenside solution (5%) for ten minutes. Small particles of mud and clay which occasionally remained in the pits of the specimens were easily removed after the ultrasound bath with a soft brush (marten hair). In some cases complete removal of the dirt was not possible except by this means. There was no damage when this method was applied, except in the case of previously repaired specimens, which fall into the formerly glued pieces and had to be glued together again after cleaning. All descriptions in this contribution refer to cleaned specimens. Members of *Salcedia* carry fine pili on wider parts of the surface and the mud adheres extremely well at these pili but also to the adhesive film. Only some representative material, and not all material, was washed in this way. This guarantees that the layer is available in the future to allow chemical investigation, if of interest. Due to cleaning, the colour and surface described in the original descriptions may differ from the true colour shown in the photographs and described in the redescriptions in this contribution. The colour of muddy specimens is not described, because differences could obviously be observed within one species depending on microhabitat. Measurements and descriptions by other authors all refer to mud-coated specimens. Therefore, results reported in this contribution might be slightly different. The identification key to the species has been constructed for cleaned specimens.

### Abbreviations

The material is deposited in the following collections:

**BMNH** (**NHMUK**) Natural History Museum, London, United Kingdom;

**CBB** Coll. Michael Balkenohl, Bonstetten near Zürich, Switzerland;

**CBP** Coll. Petr Bulirsch, Prague, Czech Republic;

**CDW** Coll. Alexander Dostal, Vienna, Austria;

**CFGC** Coll. François Génier, Gatineau, Canada;

**MFNB**Museum für Naturkunde Berlin, Germany;

**MHNM** Museu de Historia Natural de Maputo, Mozambique;

**MNHN**Muséum national d’Histoire naturelle, Paris, France;

**MRACT** Musée Royal de l´Afrique Centrale, Tervuren, Belgium;

**SMNS**Staatliches Museum für Naturkunde, Stuttgart, Germany.

Other abbreviations used:

**L/W** ratio length divided by width (used for the pronotum, elytra, and sixth antennomere);

**SSO** subapical setose organ (situated at the female coxostylus/gonopod IX);

[**sic !**] written exactly this way, as a comment by the author;

**x̄** arithmetic mean (used in Table 1 for the descriptive statistics).

## Taxonomy

### Genus Salcedia

Taxon classificationAnimaliaColeopteraCarabidae

Fairmaire, 1899

B05C2A3F-5AEB-5477-8E3E-52C354870D4E

Salcedia Fairmaire, 1899: 512.
= Zelma Andrewes, 1920: 451.

#### Type species.

*Salcedia perrieri* Fairmaire, 1899, by original designation.

#### Recognition.

Small sized (2.4–4 mm); griseous to piceous species with long oval or elongated shape of the habitus; head semi-circular or campanulate; labrum short, seven-setose; clypeus fused with clypeal wings; frons of head posteriorly with four longitudinal carinae, the median ones more or less joining anteriorly into a keel or tubercle; without clypeal and supraorbital setae; eyes either not at all or to a small extent visible in dorsal view. Head and pronotum ventrally with channel for the reception of the antennae. Pronotum with two conspicuously raised longitudinal carinae at middle, laterally with one to four additional smaller carinae, laterally broadly bent upwards, wing-like, with five or six transverse lateral pits, lateral margin crenulate to varying degrees, without marginal setae. Elytron with carinate suture, with interneurs two, four, and six sharp; lateral interneur (sixth) more or less crenulated, forming outline of elytron, interneurs two and four conspicuously raised, interneur two with distinct tubercle at base, interneur five without carina, one and three in some species with slight to indistinct carina of different lengths. Intervals with one or two longitudinal series of pits. Lateral margin and channel not visible in dorsal view, channel with a series of umbilical setigerous punctures, accompanied by interneurs seven and eight. Elytron without discal setigerous punctures. Metepisternum with longitudinal groove medially for the reception of intermediate tarsomeres and the apical part of the intermediate tibia. At the margin of the last visible abdominal sternum with one setigerous puncture at each side. All tibiae carinate, front and intermediate tarsomeres broadened.

Different from the other genera of the tribe Salcediini, mainly by the antennal channel on the ventral surface of the pronotum, by the metepisternum with longitudinal groove for the reception of the apical part of the intermediate leg, the distinctly carinate tibiae, the less visible eyes in dorsal view, and by the antennomeres two and three which are of the same length.

#### Redescription.

***Measurements:*** Body length 2.4–4 mm, width 0.9–1.5 mm.

***Colour and surface:*** Anthracite grey to fuscous or piceous, more or less shiny; top of carinae, margins of pronotum and elytra and surface of supra-antennal and supraorbital plates usually opaque, griseous, covered with pale grey pili; mandibles, legs, antennae and palpi paler.

**Figure 5.**
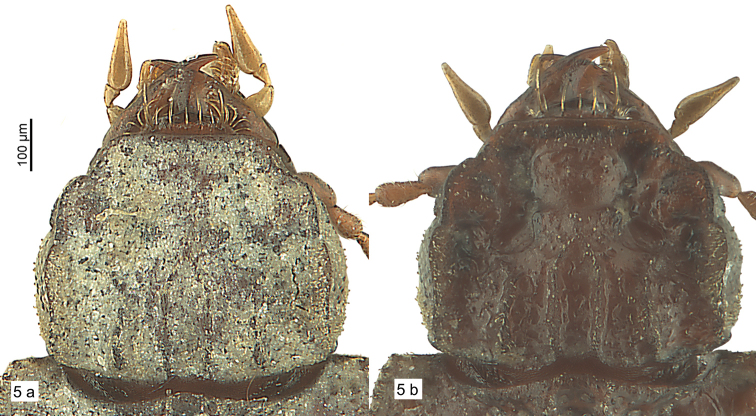
Specimen of *Salcedia lukulua* sp. nov., head, dorsal view **a** uncleaned **b** cleaned.

**Figure 6.**
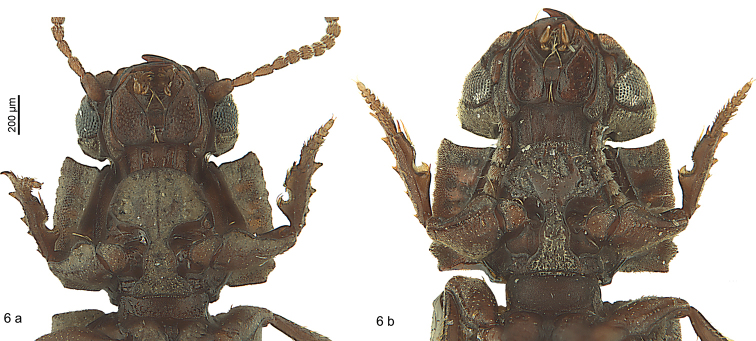
*Salcedia* species, head and pronotum, ventral view **a***S. elongata* Alluaud **b***S. baroensis* sp. nov.

***Head:*** Narrower than pronotum. Outline semi-circular, campanulate or reniform. Surface very sculptured, camouflaged by mud if not cleaned (Fig. 5). Clypeus wide, fused with clypeal wings, separated from supra-antennal plates by notches, frons with four carinae, the two median ones often joining anteriorly into a central tubercle or a longitudinal keel, with two small glossy teeth bilaterally below central tubercle, with two shorter carinae paralaterally towards base; frons and clypeus separated from supra-antennal and supraorbital plates by deep broad furrows, each furrow with moderately deep circular pit between supra-antennal plate and clypeus; margins of supraorbital plates distinctly raised. Eyes large, convex, or reduced and concave; convex eyes usually visible to small degree from above, with minute pili between ommatidia. Antenna not flattened, nearly reaching base of pronotum, densely pubescent with shorter and longer fine setae, scapus with short seta at apex, segments two to four scarcely pubescent, segment two and three of same length. Labrum short, covered for the most part by clypeus, seven-setose (example in Fig. 5). Mandible short, wide, scrobe asetose. Apical segment of maxillary palpomere securiform, terminal segment of labial palpomere short, bottle like, second segment bisetose. Glossa slender, distinctly arcuate and acute at apex. Paragena with tooth anteriorly. Mentum small, with three pairs of setae, the posterior ones pointing posteriorly, the other ones anteriorly (Fig. 6), epilobes wide, projecting and angular anteriorly, more or less margined, surface coriaciate-like, with small grooves or with flat impressions. Ligula broadened and apically truncated, with two setae close together. Head without other setae on dorsal surface.

***Pronotum:*** Outline rectangular, more or less transverse. Anterior margin bilaterally excised, the central projection carinate, fits in and hangs over the posterior excision of the head. Lateral margin straight or convex, crenulated or tuberculated. Anterior and posterior angles distinctly marked. Disc flattened or convex in lateral view, with two distinctly raised paramedian carinae more or less parallel to median line, forming two wide tubercles at base pointing posteriorly, with one to four additional shorter carinae bilaterally of which the three lateral ones are present or not, with narrow median line. Margin laterally bent upwards, wing-like, with five or six large transverse pits. Scutellum small, embedded on peduncle, somewhat hidden. No setae on dorsal surface.

**Figure 7.**
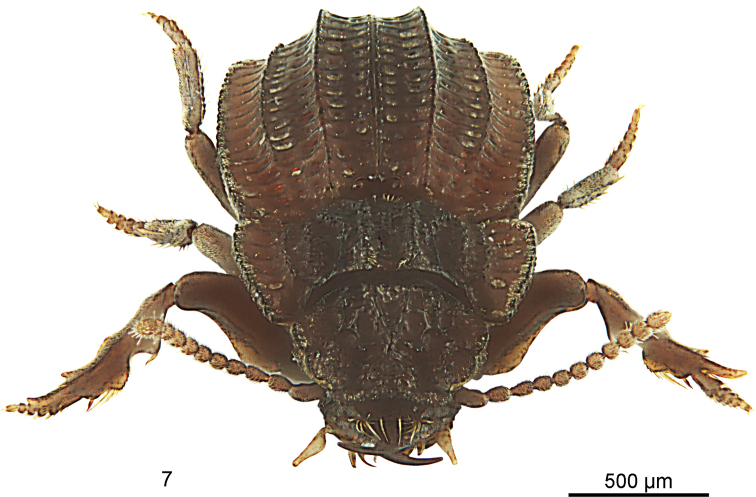
*Salcedia unifoveata* sp. nov., habitus, frontal view, cleaned specimen. The space between the suture and interneurs two, four, and six is naturally filled with mud.

***Elytron:*** Outline convex to parallel. Suture carinate. Interneurs two, four, and six sharp, distinctly to conspicuously raised (Fig. 7); lateral interneur (sixth) more or less crenulated, forming outline of elytron, forming pseudohumerus at base, interneur two with distinct tubercle at base, four at base shortened or not, interneurs one and three with or without slight carina, five without carina. If not indicated otherwise the interval on disc between suture and interneur one with two rows of pits towards base and one row of serial pits thereafter, between interneur two and three and three and four with two rows of serial pits, and between interneur four and five and five and six with two rows of serial pits, the latter ones often partly merging transversally. Pits are situated in some species on the declivity of the carinae and are not directly visible in dorsal view. No discal setae. Striole not present.

***Hind wings:*** Fully developed or reduced.

***Lower surface:*** Head with deep channel-like grooves under eyes and at lateral parts of neck for reception of antennae. Pronotum channel-like hollowed out from anterior margin nearly up to base (for reception of antenna, Fig. 6). Channel broadened towards base, surface of channel with reticulation. Elytron with lateral margin and channel not visible in dorsal view, channel narrow, accompanied by interneur seven and eight, usually more narrowed at middle, with series of umbilical setigerous punctures situated at small tubercles, interrupted at narrowed part at middle, with upright minute setae. Real humerus indistinct, obtuse-angled, situated latero-ventrally, composed of anterior end of lateral channel and obtuse carina. Pseudoepipleura (lateral interval of elytron, not visible in dorsal view) with one or two rows of pits, densely covered with pili. Lateral margin of elytron smooth or crenulated. Proepisternum carinate anteriorly, at middle and laterally. Metepisternum more or less elongate, with broad longitudinal groove (for reception of terminal part of the intermediate tibia and the intermediate tarsomeres), surface isodiametrically reticulated (Fig. 2). Metasternum and last five abdominal sternites with numerous pits, more regularly arranged at carinae and margins. Last four abdominal sternites laterally with traces of reticulation, sternite one and two densely covered with longitudinal reticulation. Sternum four to six sulcate. Last visible sternite bilaterally with one seta of medium size.

***Legs:*** Short, stout. Profemora ventro-basally wing-like dilated, dorsal surface with reticulation. Protibia with three strong short setae on ventral surface, with terminal spine arcuate at apex, laterally dentate to varying degrees, dorsally and ventrally with two carinae. Movable spur small, slightly arcuate, acute, length nearly half as long as first tarsomere; meso- and metafemora slender, slightly dilated basally, mesofemora tooth-like dilated posteriorly at base. Meso- and metatibia nearly square in cross section, with four longitudinal carinae, furnished with robust setae of moderate length. First tarsomere distinctly elongated, not quite as long as tarsomere two to five combined, front legs with tarsomeres two to four distinctly broadened and flattened. Intermediate tarsomeres moderately broadened and flattened. Claws and seta between claws relatively long.

***Male genitalia:*** Median lobe with outline moderately or slightly arcuate, in some species fractuate, apex in some species spatulate. Parameres non-pedunculate, in most of the species asetose, conspicuously asymmetrical with the ventral one very small. The median lobe is similar to the type found in the genus *Trilophidius* Jeannel, 1957.

***Female genitalia:*** Slender, coxostylus (gonopod IX) one and two fused, distinctly broadened basally, without or with one or two subapical setose organs, usually with one larger often somewhat flattened nematiform seta at beginning of basal third and five to eight slender nematiform setae. Outline similar to that of the clivinine genus *Ancus* Putzeys, 1866.

#### Distribution.

Distributed over the whole tropical belt of Africa from the west coast to the east coast, and southward to the north of the Republic of South Africa and of Madagascar. Also known from four localities in the Oriental region (Tharrawaddy, Mandalay, Palon, Calcutta).

### Special morphological features of the genus

**Channels for the reception of appendices.** The head exhibits channel like grooves on the lower surface for the reception of antennomeres four to five (Fig. 6). The groove runs between the eyes, genae and paragenae and continues along the line of the buccal fissure. On the lower surface of the pronotum, the channel continues up to the base where it is broadened. Antennomeres five to eleven fit exactly into the channel. The surface of the channel is shiny and covered with isodiametric reticulation. At the anterior margin of the pronotum and below the dorsal surface there is a tubercle at each side with a bunch of setae on the top. It exactly fits into the lower part of the gena and closes the channel laterally between the head and pronotum. The profemora is ventro-basally wing-like dilated. Together with the front tibiae, the front legs close the channel ventrally in an angled resting position.

The elongated metepisternum possesses a longitudinal groove (Fig. 2). The metatarsomeres and the apical part of the metatibia fit into this groove and close the surface in resting position. There is no groove for the hind legs. However, the hind femora are distinctly curved, allowing close attachment to the hind body. In addition the lateral margin of the elytron with the lateral channel is narrowed in the middle, where the hind femora are attached in the resting position.

This longitudinal groove on the metepisternum is not present in *Solenogenys*. It is intimated as flat longitudinal depression in *Holoprizus*.

**Layer on the surface.** Compared to other Carabidae and especially Clivinini, the species of *Salcedia* show a remarkable layer on the surface of the integument, visible with optical microscopes after removal of the mud by short cleaning. The layer covers the dorsal surface of the head, pronotum and elytra (Fig. 46). It continues laterally to the pseudoepipleura. The layer is adhesive and possibly contains wax-like chemicals. It is not completely transparent and relatively thick. Small particles of mud and sediment adhere to the surface of the layer, often very densely. One of the species (*S. unifoveata* sp. nov.) does not possess this thick adhesive layer. However, the pores and pits on the surface of that species are filled with mud and clay increasing laterally. In all of the species, this layer gives the surface an even, dull appearance making recognition of characters difficult, even when only the wax-like layer is present, without mud. For example, the head and pronotum appear flattened (Fig. 5). In immature specimens the layer is more transparent and not as dirty as in adults. The ventral surface is nearly free of this film. However, the pits and grooves are often filled with dirt. The colour of the dirt obviously depends on the microhabitat where the specimen was collected. Without removing the dirt and the film, specific characters and the colour cannot be determined exactly. Members of *Holoprizus* and *Solenogenys* have such a layer as well. However, it is not as evident as in *Salcedia*, and does not carry such a large quantity of mud.

**Setae on the surface.** Usually, longer nematiform setae (macrochaetae), often used for taxonomy in Scaritinae and especially Clivinini, are generated from distinct setigerous punctures. They are of a yellow to brown colour, are quite robust, tapering to the apex, ending acutely, are distant, and often erect. They are present in *Salcedia* in the following parts of the body: The labrum shows a total of seven setae with four long ones, two of medium size and the seta at middle is very small and usually completely hidden by the clypeus. In addition, the labrum is ciliate laterally. The generally small mentum shows a pair of setae at base and two pairs anteriorly. Setae of the other mouthparts (palpi, glossa, ligula) and the antennae exhibit a structure of setae as in other Clivinini, including a short stout apically situated seta on the scapus. The pronotum shows a group of setae at its base, which are usually arranged bilaterally as a group of four to eight setae. These setae are located opposite to the two basal tubercles of interneur two of the elytron. In addition, there is a bunch of such setae situated at the top of the two tubercles which are situated ventro-anteriorly of the anterior margin of the pronotum. The elytron shows a small basal tubercle with setigerous puncture bearing a distinct seta of medium length, situated at the basal declivity at level of interneur one. The lateral channel of the elytron, situated ventro-laterally (not visible in dorsal view), exhibits a series of light-beige umbilical setae generated from tuberculate punctures. There are longer and shorter setae which are generally thin (Fig. 2). The front, intermediate and hind legs as well as the trochanters exhibit basally one seta each. Abdominal sternum six shows one medium sized ambulatory seta at each side in the male and female. The legs show robust setae which are in general quite short. Between the claws the onychium is developed as a distinct seta, which is as long as the claws.

The following parts of the dorsal surface are covered with fine, short, soft, acutely ending, grey, and tight-fitting pili: Lateral margins of the head, parts of the supraorbital and supra-antennal plates, pronotum and elytra. In addition, all carinae on the head, pronotum and the elevated interneurs of the elytron show a band of these pili bilaterally to the top, sometimes also on the top. Moreover, slightly stronger pili are present at the declivity of the basal tubercles of the pronotum and elytra. The eyes also show short pili, arising from the interspaces of the ommatidia.

Furthermore, there is another type of fine short pili present at the lateral margins of the head, pronotum and elytra. These pili are less numerous than the small grey pili, club-like and slightly larger in diameter, rounded at the tip and often of beige to grey colour. These second type of pili are quite easily removed when robust cleaning of specimens is applied.

In general, and obviously depending on the age of the specimen, all of the setae are often rubbed off with the exception of the abdominal ambulatory setae which are present in nearly all specimens.

Number, location and appearance of setae and pili on the external surface are very similar among the species and do not exhibit much difference that is usable for species differentiation. However, in *S. baroensis* sp. nov., the pili are less numerous, in *S. miranda* the pilosity is denser.

The following setae, usually present in most of the other Clivinini, are missing: Clypeal setae, supraorbital setae, lateral pronotal setae, and discal setae of the elytron.

In *Salcedia* the female coxites 1 and 2 are fused (gonopod IX). They show a characteristic pattern of nematiform setae in the basal half. In addition, in most of the species there is a subapical setose organ (vestigial gonostylus) present generating one or two microtrichia.

### Identification key to the species of *Salcedia* (for cleaned specimens)

The small species are not easy to discriminate, if not trained. It is recommended to compare the results of the identification with the data provided in Tables 1, 2 as well as with the description of the genital characteristics.

**Table:** 

1	African species; frons of head with paramedian carinae converging anteriorly and joining into a keel or tubercle	**2**
–	Oriental species; head with paramedian carinae converging anteriorly but not completely joining, not forming a keel or tubercle	**17**
2	Species from Madagascar; with or without conspicuously reduced eyes	**3**
–	Species from continental Africa; always with well-developed eyes	**5**
3	Eyes conspicuously reduced, not visible in dorsal view, in lateral view strikingly small, embedded between lateral carinae and genae, concave; lateral margin of pronotum with 16 tubercles; intervals of elytron with one serially row of pits; body length 2.6–3.1 mm	***S. unifoveata* sp. nov.**
–	Eyes well developed, partly visible in dorsal view, in lateral view large, convex; lateral margin of pronotum with 8–12 tubercles; intervals of elytron with more than one row of pits	**4**
4	Lateral margin of pronotum with 12 tubercles; head with raised tubercle in middle; pseudohumerus of elytron with laterally projecting tooth; body length 3.2–3.6 mm	***S. perrieri* Fairmaire, 1899**
–	Lateral margin of pronotum with 8–9 tubercles; head with keel at middle; pseudohumerus of elytron without projecting tooth; body length 3.1 mm	***S. faillei* sp. nov.**
5	Outline of elytron in dorsal view distinctly convex; species smaller than 3.0 mm (but see exception *S. baroensis* sp. nov. at 3.7 mm)	**6**
–	Outline of elytron in dorsal view parallel or slightly rounded with straight or indistinctly rounded parts; species larger than 3.0 mm	**8**
6	Pronotum conspicuously transverse with ratio length/width 0.55–0.59, with row of five transverse lateral pits, lateral margin with 9 tubercles; head reniform, posterior genae convex, without angle (dorsal view); smallest species; body length 2.4–2.8 mm	***S. coquilhati* Alluaud, 1932**
–	Pronotum moderately transverse with ratio length/width 0.72–0.75, with row of six transverse lateral pits, lateral margin with 12–13 tubercles; head semi-circular or campanulate, posterior genae with distinct angle (dorsal view), without convexity	**7**
7	Paramedian carinae on frons of head joining V-like anteriorly and ending with minute tubercle; antenna with segments five to ten sub-elongate (L/W 1.16); base of pronotum produced wing-like posteriorly, distinctly emarginated bilaterally towards middle; head obtuse angled postero-laterally (angle 137°); body length 2.8 mm	***S. lukulua* sp. nov.**
–	Paramedian carinae on frons of head joining into a distinct keel; antenna with segments five to ten elongate (L/W 1.24); base of pronotum not produced posteriorly, with slight notch bilaterally towards middle; head postero-laterally with distinct angle (around 122°); body length 3.3–3.4 mm	***S. matsumotoi* sp. nov.**
8	Elytron super-elongate or oblong-elongate, with lateral outline parallel, with interneur two parallel at middle	**9**
–	Elytron sub-elongate or elongate, with outline slightly convex or slightly dilated posteriorly with straight or indistinctly rounded parts in basal third only, with interneur two convex at middle	**12**
9	Elytra super-elongate with lateral outline nearly parallel but slightly to indistinctly concave at middle, maximum width before and behind middle; antennomeres with joint 5–10 sub-moniliform (L/W 0.95–1.02); body length 3.1–3.4 mm	***S. procera* sp. nov.**
–	Elytra oblong-elongate with lateral outline parallel, straight, maximum width at middle; antennomeres with joint 5–10 moniliform or sub-elongate	**10**
10	Base of pronotum bilaterally with distinct tubercle pointing posteriorly; elytron with distinct tubercle at base of interneur four; pronotum one fifth wider than long; body length 3.4 mm	***S. tuberculata* sp. nov.**
–	Base of pronotum without distinct tubercle; elytron without tubercle at base of interneur four; pronotum one third or one quarter wider than long	**11**
11	Pronotum a quarter wider than long; pronotum bilaterally with two additional carinae; elytron with interneur four shortened at base; Elytron with interneur two shortened at apex; antennomeres with joint 5–10 moniliform, male median lobe with apex long, slender, apex stick-like in cross section; body length 3.3–3.7 mm	***S. elongata* Alluaud, 1932**
–	Pronotum one third wider than long; pronotum bilaterally with three additional carinae; elytron with interneur four running up to base; Elytron with interneur two running distinctly up to apex; antennomeres with joint 5–10 super-moniliform; male median lobe with apex continuously narrowed to apex, apex flattened in cross section; body length 3.3–4 mm	***S. africana* (Britton, 1947)**
12	Pronotum with paramedian carinae conspicuously raised, interrupted by distinct deep notches; lateral margin of pronotum with 8–(10) tubercles; body length 3.5–4.2 mm	***S. robusta* sp. nov.**
–	Pronotum with paramedian carinae moderately raised, more or less continuing, not interrupted by distinct notches; lateral margin of pronotum with 10–15 tubercles	**13**
13	Antennomeres with joints five to ten oblong-elongate (ratio length/width 1.41); elytra shorter, length 1.71–1.76 mm; elytra slightly diverging posteriorly, maximum width behind middle, interneur three developed as slightly raised carina; body length 3.5–3.9 mm	***S. baroensis* sp. nov.**
–	Antennomeres with joints five to ten elongate (ratio length/width 1.25–134); elytra longer, length 2.03–2.38 mm; elytra slightly convex laterally but not diverging, with maximum width at middle, interneur three without raised carina	**14**
14	Pronotum with all three lateral carinae complete; elytron with pseudohumerus obtuse-angled (angle around 97°); elytron with lateral margin (not visible in dorsal view) smooth; median lobe of aedeagus at apex spatulate in total, in cross section with concavity; female coxostylus with eight nematiform setae and with one SSO; body length 3.4–3.6 mm	***S. utetea* sp. nov.**
–	Pronotum with lateral carinae incomplete, two of the three carinae completely missing; elytron with pseudohumerus nearly right-angled; elytron with lateral margin (not visible in dorsal view) sub-crenulate; median lobe of aedeagus at apex ovoid in cross section	**15**
15	Pronotum with lateral margin regularly convex with maximum width at middle, with 10 to 11 tubercles; elytron with lateral outline straight at middle; head baso-laterally with obtuse angle (angle 120–123°); body length 3.1–3.9 mm	***S. putzeysi* (Oberthür, 1883)**
–	Pronotum with lateral margin either regularly convex or diverging anteriorly with maximum width behind middle, with > 12 tubercles; elytron with lateral outline convex or converging and with straight parts; head baso-laterally with angle <110°	**16**
16	Lateral margin of pronotum convex and converging anteriorly, lateral tubercles indistinct; elytron with lateral outline with straight and almost parallel part directly posterior pseudohumerus; pseudohumerus with distinct tooth-like tubercle; median lobe of aedeagus with apex sinuate, in cross section oval; female coxostylus with six nematiform long setae and one SSO; body length 3.4–3.8 mm	***S. schoutedeni* Alluaud, 1930**
–	Lateral margin of pronotum slightly and regularly convex, lateral tubercles distinct; elytron with lateral outline slightly convex; pseudohumerus without or with indistinct projecting tooth; median lobe of aedeagus straight, pointing dorsally, flattened preapically and in cross section oval at apex; female coxostylus with eight nematiform setae; body length 3.3–3.7 mm	***S. nigeriensis* Alluaud, 1932**
17	Elytron without laterally projecting tooth at pseudohumerus, interval between interneurs four and five and five and six with two serially rows of pits; lateral margin of pronotum convex; antennomeres five to ten oblong-elongate (ratio length/width 1.44); body length 4 mm	***S. miranda* (Andrewes, 1920)**
–	Elytron with distinct laterally projecting tooth at pseudohumerus, interval between interneurs four and five with two serially rows of pits, and between interneurs five and six with one row of serial pits; lateral margin of pronotum straight; antennomeres five to ten elongate (ratio length/width 1.22); body length 3.6 mm	***S. parallela* Baehr, 1998**

### Salcedia perrieri

Taxon classificationAnimaliaColeopteraCarabidae

Fairmaire, 1899

2539ACAE-EF2E-5F4D-A7DF-755F030D1F55

Figs 1 , 3 , 8 , 26 , 44 , 52 , 68 , 84

Salcedia perrieri Fairmaire, 1899: 512; Csiki 1927: 546; Alluaud 1930: 22; Jeannel 1946: 233; Basilewsky 1973: 293; Reichardt 1975: 103; Peyrieras 1976: 157; Dostal 1993: 121; Deuve 1993: 141; Lorenz 2005: 155.

#### Type material.

**Holotype**: ♂, with labels and data: brownish, printed, black framed “Madag^r^. Suberb^lle^. H.Perrier” (MNHN).

#### Remark.

The holotype is complete. The specimen was located in the collection Fairmaire in MNHN.

**Figures 8–13.**
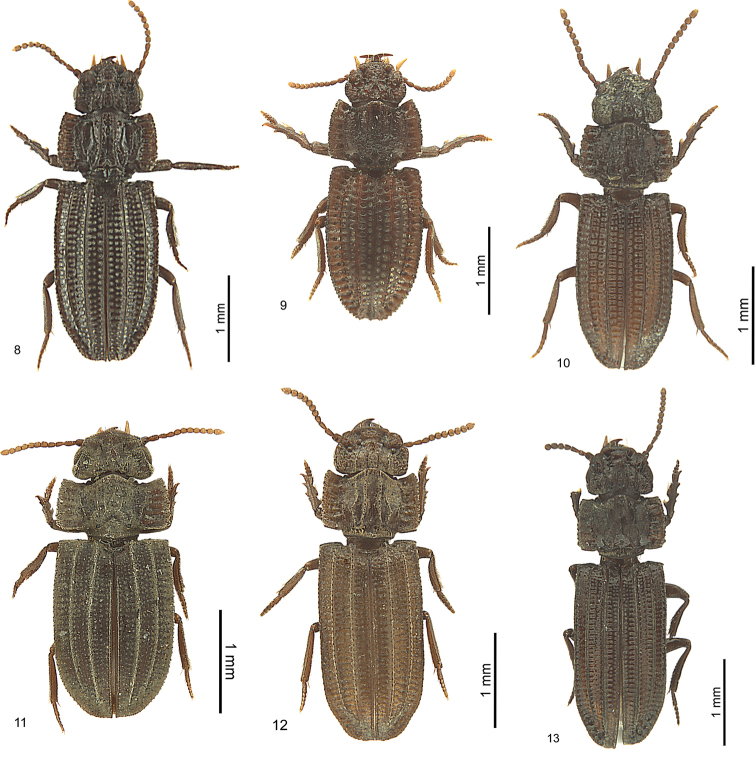
*Salcedia* species, habitus, dorsal view, holotype **8***S. perrieri* Fairmaire **9***S. unifoveata* sp. nov. **10***S. faillei* sp. nov. **11***S. coquilhati* Alluaud **12***S. elongata* Alluaud **13***S. africana* (Britton).

#### Additional material.

1 spec., Madagascar Ron Maroantsetra V 35 Vadon ! (MNHN, CBB); 29 specs., Madagascar Ron Maroantsetra IV 38 Vadon ! / Andranofotsy (MNHN); 2 ♂, 2 specs., same data but with label COLL. MUS. Congo Madagascar : Col.P.Basilewsky / one of them with additional blue label: MUSÉUM PARIS 1938 J. VADON et E. LEBIS (MRACT); 4 specs., Madagascar Ron Maroantsetra IV 38 Vadon ! / Antakotako (MNHN); 1 spec., same data but Mahakiry (MNHN); 1 ♀, 3 specs., Madagascar, Fampanambo XII.1958 86 (Inondations) / MUS. ROY. CENTR. Madagascar Est: Bale d’Antongli J. Vadon (MRACT); 1 spec., same data but Maroantsetra Fampanambo III. 56 (MRACT); 1 spec., Madagascar: Fampanambo II.1960 J. Vadon / *Salcedia perrieri* Frm. P. Basilewsky det., 1970 / COMP. TYP. BASILEWSKY (MRACT); 1 spec., same data but III 65 (Vadon) (MNHN); 1 spec., Maroantsetra Ambodivoangy J.Vadon / INSTITUT SCIENTIFIQUE MADAGASCAR (MNHN); 11 specs., Ankofa VII 56 n. 7 / INSTITUT SCIENTIFIQUE MADAGASCAR (MNHN, CBB); 1 ♂, 3 ♀, 1 spec., Ankofa XII. 55 n. 7 / INSTITUT SCIENTIFIQUE MADAGASCAR / COLL. MUS: TERVUREN (ex Muséum Paris) Coll. P. Basilewsky (MRACT); 1 spec., Tananarive Tsimbazaza / INSTITUT SCIENTIFIQUE MADAGASCAR / *Salcedia perrieri* Fairm. P. Basilewsky det. 1971 (MNHN); 1 spec., same data but with add. label COLL. MUS: TERVUREN (ex Muséum Paris) Coll. P. Basilewsky (MRACT); 1 ♂, 2 ♀, MADAGASCAR c. Antsiranana prov., 2002 Ambanja, Ambodidimaka r. 15–16.12., Ivo Jeniš leg. (CBP); 1 spec., Madagascar Ron Maroantsetra IV 38 Vadon ! / Andranofotsy / *Salcedia perrieri* Fm. / COLLECTIO KAREL KULT COLL.A.DOSTAL, 1999 / Coll. P. Bulirsch Prague Czech Republic (CBP); 1 ♀, MADAGASCAR ?? Perinet 1989 (CBP); 1 spec., right antenna, right hind tibia and right intermediate tarsomere missing, MADAGASCAR, Ambanja Antsiranana pr. XII. 2002 / BMNH (E) 2008-52 P.Bulirsch/ SALCEDIA perrieri Fairmaire, 1899 P.Bulirsch det. 2011 (BMNH); 2 specs., Madagascar Ron Maroantsetra IV 38 Vadon! / Andranofotsy / with additional blue label: MUSÉUM PARIS 1938 J. VADON et E. LEBIS / with additional white label: BRIT.MUS. 1947-246. (BMNH); 1 ♀, Madag Perrier (MNHN); 1 ♂, Morondava fôret sud de Befasy I-56 R.P. / INSTITUT SCIENTIFIQUE MADAGASCAR (MNHN).

#### Diagnosis.

A medium sized species, with sub-elongate oval outline of the elytra with maximum width at middle and the pronotum with three additional lateral carinae. The pseudohumerus is nearly rectangular and distinctly dentate. The antennomeres are elongate. Distinguished most clearly from the similar species *S. faillei* sp. nov. by the tubercle on the frons of the head, the twelve tubercles at the lateral margin of the pronotum, and the pseudohumerus of the elytron which shows a laterally projecting tooth. Moreover, the median lobe of the male genitalia is different and the dorsal paramere has a pilus at the apex.

#### Redescription.

Measurements in Table 1.

***Colour and surface:*** Anthracite grey, opaque; top of carinae on head, pronotum and elytra shiny; mandibles and legs piceous, antennae and palpi leoninous.

***Head:*** Two-thirds of width of pronotum. Outline semi-circular. Clypeus wide, straight anteriorly, separated from convex clypeal wings by indistinct obtuse notches, clypeal wings separated from supra-antennal plates by distinct notches, with raised pentagon-shaped field at middle, separated from frons by deep transverse furrow, frons with two raised paramedian carinae, joining anteriorly, prolonged anteriorly into a central distinctly erected tubercle, with two small glossy teeth bilaterally anterior to central tubercle, with two parallel carinae paralaterally near base; frons and clypeus separated from supra-antennal and supraorbital plates by deep broad furrows, each furrow with deep circular pit between supra-antennal plate and clypeus; supra-antennal and supraorbital plates distinctly margined, margin raised, carina-like, indistinctly crenulated, supra-antennal plates vaulted. Base emarginated at middle, obtuse angled laterally (angle 120°). Eyes convex, genae slightly convex, both of them partly visible from above, with pentagonal shape in lateral view. Antenna with segments five to ten elongate (L/W 1.26), densely pubescent, segments two to four scarcely pubescent, scapus with longitudinal reticulation. Labrum excised anteriorly. Mandible moderately short, wide, slightly arcuate at apex. Mentum small, epilobes wide, projecting and obtuse angled anteriorly, completely margined, surface coriaciate-like with flat impressions.

***Pronotum*** (Fig. 26): Outline rectangular, transverse, a third wider than long. Lateral margin convex, maximum width at middle, converging anteriorly and posteriorly. Lateral margin distinctly crenulated, with 12 tubercles, with distinct emargination at posterior angles. Base posteriorly moderately wing-like produced, with distinct notch between wing and central part of base, straight posteriorly. Declining flat keel at middle of base pointing posteriorly. Disc slightly convex (lateral view), with two raised paramedian carinae parallel to median line, slightly diverging posteriorly, with median line small, short, ending in pits anteriorly and posteriorly, with four additional shorter carinae bilaterally, the paralateral one joining with the paramedian carinae at base and forming a tooth-like tubercle pointing posteriorly, extended anteriorly as less raised paralateral carina. Two much shorter and less raised inner and outer lateral carinae, the inner one joining with the anterior extension of paralateral carina, all carinae sub-crenulate. Lateral margin and space between lateral margin and paralateral carina wing-like bent up, with six large transverse pits, the basal ones partly separated into two small circular pits.

***Elytron*** (Fig. 44): Flattened in anterior half (lateral view), convex in frontal view. Sub-elongate, margin moderately convex, maximum width at middle. Pseudohumerus nearly rectangular, distinctly dentate. Apex rounded, acutely denticulate at suture. Disc with interneur six sub-crenulate, interneur two running up to apex as slightly convex line, conspicuously raised; interneur four running in parallel to interneur six, not reaching base, shortened at apex. Interneur five and six with two rows of serial pits, the latter ones partly doubled at base and transversally connected apically.

***Hind wings:*** Fully developed.

***Lower surface:*** Antennal channel of pronotum with isodiametric reticulation. Pseudoepipleura with one row of serial pits, lateral margin of elytron smooth. Metasternum and last five abdominal sternites with numerous irregularly situated larger and smaller pits, regularly arranged at carinae and margins.

***Legs:*** Profemora with irregular pits on dorsal surface. Protibia laterally with two larger and one smaller tooth, dorsally with one and ventrally with two carinae. Metafemora with small, short grooves at dorsal surface.

***External sexual dimorphism:*** Not observed.

***Male genitalia*** (Fig. 52): Median lobe large, moderately slender, in lateral view nearly straight, in dorsal view slightly arcuate in basal half and at middle, distinctly arcuate in apical third, with few fine pili at beginning of apical third, apex stick-like. Endophallus with two longitudinal groups of densely situated microtrichia, groups as long as one third of median lobe. Dorsal paramere moderately wide at base, bisinuate, with pilus at apex, lateral and basal apophysis blunt; ventral one of moderate size; both parameres somewhat distorted.

***Female genitalia*** (Fig. 68): Coxostylus moderately broad at base, obtusely curved, moderately broad in apical third, rounded at apex, at end of basal third with one broader and five slender nematiform setae laterally, with one long slender nematiform seta ventrally, SSO with one microtrichium.

***Variation:*** The carinae on the frons of the head are tuberculated to a different degree. The number and shape of the pits on the ventral surface varies to a small degree. Slight differences could also be observed with the flat impressions on the lower surface. The female specimen from Perinet (label indicating uncertain location) is slightly larger than the other ones but in all other characters matches *S. perrieri*.

#### Distribution

(Fig. 84): Known from several localities in north-east and Central Madagascar. As far as the label information allows it can be stated the specimens were collected at the margins of inland waters. Some of the specimens were collected after flooding. The type locality Suberbieville is located on the River Ikopa near Maevatana in the north-west of Madagascar.

### Salcedia unifoveata sp. nov.

Taxon classificationAnimaliaColeopteraCarabidae

C171B614-D98D-503E-BA55-C6817CED53DC

http://zoobank.org/17AA3B99-C9A0-4FB5-B371-C7921872FD1C

Figs 7 , 9 , 27 , 45 , 53 , 69 , 84

#### Type material.

***Holotype:*** ♂, with labels and data: white, black printed “MUS. ROY. CENTR. Madagascar Est : Bale d’Antongli J. Vadon” / black printed and handwritten “Ambodivoangy II. 1959 (Lavage de terre) 12.” (MRACT). ***Paratypes***: 3 ♀♀, same data as holotype; 1 ♀, same data as holotype but “05.”; 1 ♀, same data as holotype but “21.” (MRACT, CBB).

#### Diagnosis.

A medium sized species, with long-ovoid outline of the elytra with maximum width at middle and pronotum with three additional carinae and at the lateral margin with eight to nine tubercles. The pseudohumerus is obtuse angled and not dentate. The antennomeres are sub-elongate. Distinguished from all other species of the genus by the very small and laterally embedded eyes, and the elytron with one row of pits between the suture and the interneurs. In addition, the hind wings are reduced to threadlike rudiments.

#### Description.

Measurements in Table 1.

***Colour and surface:*** Anthracite grey, pronotum laterally and elytra shiny; pronotum laterally and antero-lateral part of elytra slightly translucent piceous, top of carinae on head, pronotum and elytra shiny; mandibles, legs and antennae piceous, palpi leoninous. Surface without adhesive layer. Short pili on surface less numerous compared to other species.

***Head:*** Two-thirds of width of pronotum. Outline pentagonal. Clypeus wide, clypeal wings small, nearly fused with clypeus, separated from supraorbital plates by distinct rectangular notches, with raised pentagon-shaped field at middle, separated from frons by deep transverse furrow, frons with two raised paramedian carinae, joining anteriorly, prolonged anteriorly into a flat central keel, with two small glossy tooth-like tubercles laterally anterior to keel and two large tubercles paralaterally at genae level; frons and clypeus separated from supra-antennal and supraorbital plates by deep broad furrows; each furrow with deep circular pit between supra-antennal plate and clypeus; supra-antennal and supraorbital plates distinctly margined, margin sub-crenulate, supra-antennal plate vaulted, separated from supraorbital plate by distinct notches. Base slightly emarginated at middle, laterally narrowed and rounded off without angle. Eyes reduced, conspicuously small (lateral view), reniform, concave, genae developed as small obtuse carinae. Antenna with segments five to ten sub-elongate (L/W 1.14), densely pubescent, segments two to four scarcely pubescent. Labrum short, straight anteriorly. Mandible moderately short, slender and arcuate in apical half, right one broadened in basal h Glossa slightly arcuate, obtuse at apex. Mentum small, epilobes wide, projecting and obtuse angled anteriorly, completely margined, with carina medially, surface coriaciate-like with flat impressions.

***Pronotum*** (Fig. 27): Outline rectangular, moderately transverse, a quarter wider than long. Anterior margin bilaterally excised. Lateral margin convex, maximum width behind middle, converging distinctly anteriorly and slightly posteriorly. Anterior angles acute, posterior angles obtuse. Lateral margin distinctly crenulated, with 17–18 tubercles, with small emargination at posterior angles. Base straight laterally, with declining flat keel at middle pointing posteriorly. Disc with two raised paramedian carinae parallel to middle, slightly diverging posteriorly, raised keel-like in anterior quarter, with flat broad median line deepened in longitudinal pits anteriorly and posteriorly, with four additional shorter carinae bilaterally, the paralateral one joining with the paramedian carinae at base and forming at each side tooth-like tubercle pointing posteriorly, extended anteriorly as less raised paralateral carina. With two much shorter and less raised inner and outer lateral carinae, the inner one joining with the anterior extension of paralateral carina. Lateral margin and space between lateral margin and paralateral carina wing-like bent up, with five large transverse and two small circular pits.

***Elytron*** (Fig. 45): Slightly and regularly convex anterior half (lateral view), convex in frontal view. Anterior sixths of margin straight and laterally bent up, posterior five sixths long-ovoid, maximum width at middle. Pseudohumerus nearly obtuse angled, distinctly dentate. Apex rounded, with concave emargination, acutely denticulate at suture. Disc with interneur six crenulated, interneur two running apically as slightly convex line, ending distinctly before reaching apex, conspicuously raised; interneur four running subparallel to interneur two, distinctly abbreviated at base and apex. Interval between suture and interneur one and between the other interneurs with one row of serial pits, and between four and six with two rows of transversally connected pits, transverse connection of the latter ones is so intense that the interneur line is hardly recognisable.

***Hind wings:*** Reduced to threadlike rudiments.

***Lower surface:*** Antennal channel of pronotum with isodiametric reticulation. Pseudoepipleura with one row of serial pits, lateral margin of elytron smooth. Metepisternum of moderate length, with broad longitudinal groove. Metasternum and last five abdominal sternites with numerous irregularly situated larger and smaller pits, regularly arranged at carinae and margins. Sternum four to six obtusely sulcate.

***Legs:*** Profemora with few transverse wrinkles on dorsal surface. Protibia with terminal spine hook-like at apex, laterally with two teeth, dorsally with one and ventrally with two carinae. Movable spur short, length a quarter of first tarsomere. First tarsomeres distinctly elongated, almost as long as tarsomeres two to five together.

***External sexual dimorphism:*** Not observed.

***Male genitalia*** (Fig. 53): Median lobe moderately broad, in lateral view indistinctly bisinuate, in dorsal view slightly cracked at apical third, nearly straight to apex, with some fine pili at beginning of apical third, apex spatulate, spatula almond-shaped in cross section, distorted. Endophallus with small group of spines. Dorsal paramere slender, sinuate, lateral and basal apophysis blunt; ventral one short. Both parameres slightly distorted.

***Female genitalia*** (Fig. 69): Coxostylus slender at base, distinctly curved, slender in apical third, at end of basal third with one broader and five slender nematiform setae close together laterally, with one long slender nematiform seta ventrally, SSO with one microtrichium.

***Variation:*** The number and shape of the pits on the dorsal surface varies to a certain degree.

#### Etymology.

The name refers to the single row of pits on the elytron between the suture and the interneurs, which is unique in the whole genus (Latin *unus* = one, *fovea* = pit).

#### Distribution

(Fig. 84): Known only from the type locality, Ambodivoangy in Central Madagascar, located by a lake. The specimens were collected by soil washing.

### Salcedia faillei sp. nov.

Taxon classificationAnimaliaColeopteraCarabidae

E1F1F136-A434-5310-B47B-738FBDA5CE30

http://zoobank.org/0BB41F29-24D5-4DD7-8740-AE40DFA8FA2F

Figs 10 , 28 , 54 , 84

#### Type material.

**Holotype**: ♂, with labels and data: white, black printed “S MADAGASCAR Isalo N.P., 11–12.1. Ranohira env., 2010 F.Pavel leg., 825 m / 22°33'07,5"S, 045°24'49,5"E (SMNS).

#### Diagnosis.

A small sized species, with elongate oval outline of the elytra with maximum width slightly behind middle and the pronotum with three additional carinae. The pseudohumerus is nearly rectangular and not dentate. The antennomeres are elongate. Distinguished most clearly from the similar species *S. perrieri* by the keel on the frons of the head, the eight to nine tubercles at the lateral margin of the pronotum, and the pseudohumerus of the elytron which is also right angled but without projecting tooth. The male genitalia has a different median lobe and the ventral paramere is setose at apex.

#### Description.

Measurements in Table 1.

***Colour and surface:*** Piceous to fuscous; top of carinae on head, pronotum and elytra as well as intervals shiny; legs, mandibles, scapus, and pedicellus piceous, antennomeres three to eleven and palpi leoninous.

***Head:*** Three-quarters of pronotum width. Outline campanulate. Clypeus wide, straight anteriorly, not separated from convex clypeal wings, clypeal wings separated from supra-antennal plates by distinct notches, with raised pentagon-shaped field at middle, separated from frons by flat transverse furrow. Frons with two raised paramedian carinae, joining anteriorly, prolonged anteriorly into central keel, with two small teeth bilaterally anterior to central keel, with two carinae paralaterally near base converging anteriorly; frons and clypeus separated from supra-antennal and supraorbital plates by deep broad furrows, each furrow with deep circular pit between supra-antennal plate and clypeus and another pit bilaterally to clypeus; supra-antennal and supraorbital plates distinctly carinate, indistinctly crenulated, supra-antennal plates vaulted. Base emarginated at middle, obtuse angled laterally (angle 128°). Eyes convex, genae slightly convex, both of them partly visible from above, with transverse-oval shape in lateral view. Antenna with segments five to ten elongate (L/W 1.22), densely pubescent, segments two to four scarcely pubescent, scapus with longitudinal reticulation. Labrum straight. Mandible moderately short, wide, slightly arcuate at apex. Mentum moderately sized, with isodiametric reticulation, epilobes wide, projecting and acute angled anteriorly, amargined, surface covered with flat pits.

***Pronotum*** (Fig. 28). Outline rectangular, transverse, a third wider than long. Lateral margin convex, maximum width slightly behind middle, converging anteriorly and posteriorly. Lateral margin distinctly crenulated, with eight to nine tubercles, with distinct emargination at posterior angles. Base posteriorly slightly wing-like produced, with slight notch between wing and central part of base, straight posteriorly. Declining flat keel at middle of base broad, pointing posteriorly. Disc slightly convex (lateral view), with two raised paramedian carinae parallel to median line and slightly diverging posteriorly, median line invisible, with four additional shorter carinae bilaterally, the paralateral one joining with the paramedian carinae at base and forming tooth-like tubercle pointing posteriorly, extended anteriorly as less raised paralateral carina. With two much shorter and less raised inner and outer lateral carinae, all outer carinae connected to each other, smooth. Lateral margin and space between lateral margin and paralateral carina wing-like bent up, with six large transverse pits, transverse pits consisting of two large circular and connected pits.

***Elytron:*** Flattened in anterior half (lateral view), flattened at middle in frontal view and moderately convex laterally. Elongate, margin moderately convex, maximum width at middle. Pseudohumerus slightly obtuse angular (angle 104°), distinctly angulate. Apex rounded, acutely denticulate at suture. Disc with interneur six indistinctly crenulated, interneur three convex in basal third, interneur two running up to apex as slightly convex line, distinctly raised; interneur four running in parallel to interneur six, distinctly not reaching base, shortened at apex. Interneur five and six with three rows of transversally connected pits, connection of the latter ones is of such intense that the interval line is hardly recognisable.

***Hind wings:*** Fully developed.

***Lower surface:*** Antennal channel of pronotum with isodiametric reticulation. Pseudoepipleura with one row of serial pits, lateral margin of elytron smooth. Metasternum and last five abdominal sternites with numerous irregularly situated larger and smaller pits, regularly arranged at carinae and margins.

***Legs:*** Profemora with irregular reticulation on dorsal surface. Protibia laterally with two larger and two fine teeth, dorsally with one and ventrally with two carinae. Metafemora with irregular reticulation at dorsal surface.

***External sexual dimorphism:*** Not observed.

***Male genitalia*** (Fig. 54). Less sclerotised as other species. Median lobe short, broad in basal half, slender in apical third, in lateral view indistinctly bisinuate, in dorsal view slightly cracked in at apical third, straight to apex, with some fine pili at middle and at beginning of apical third, apex oval in cross section, slightly bent dorsally. Endophallus with longitudinal group of robust spines, group as long as one fifth of median lobe, with second short group adverse. Dorsal paramere slender, sinuate, apophyses elongated; ventral one moderately long, with minute seta at apex; both parameres slightly distorted.

***Female genitalia:*** Unknown.

***Variation:*** Slight intra-individual variation between the two lateral margins of the pronotum was observed in the number of tubercles.

#### Etymology.

The species is dedicated to Dr. Arnaud Faille (SMNS) who particularly supported this contribution and who made me aware of this species.

#### Distribution

(Fig. 84). Known from the type locality in the south of Madagascar.

### Salcedia coquilhati

Taxon classificationAnimaliaColeopteraCarabidae

Alluaud, 1932

9A02A9FC-7D49-5695-BB7D-B3E4F23798EE

Figs 11 , 29 , 55 , 70 , 84

Salcedia coquilhati Alluaud, 1932: 3; Csiki 1933: 641; Alluaud 1935: 19; Burgeon 1935: 159; Reichardt 1975: 103; 1932, Dostal 1993: 121; Lorenz 2005: 155.

#### Type material.

***Holotype:*** ♂, with labels and data: white, printed and handwritten “MUSÉE DU CONGO Coquilhatville –XII– 1924 D^r^H.Schouteden” / red, glued “TYPE” / white printed and handwritten “R. DÉT. 2019 f” / white, black framed, handwritten and printed “*Salcedia coquilhati* All. Alluaud det. 19” / red, black double framed “TYPE” / white, black printed “RMCA ENT 000019377” and square barcode (MRACT). ***Paratype***: 1 ♀, with labels and data: white, printed and handwritten “MUSÉE DU CONGO Coquilhatville -XII- 1924 D^r^H.Schouteden” / white, handwritten “*Salcedia coquilhati* n.sp. Ch. Alluaud det.” / red, black double framed “PARATYPE” (MRACT).

#### Remark.

In the holotype the left anterior leg is missing and the hind body shows some slight damages. In the paratype the left hind leg is missing.

#### Additional material.

1 ♂, 1 ♀, 1 spec., Eala I-1935 and XI-1934 J.Ghesquière MUSÉE DU CONGO; 1 ♂, 1 ♀, 2 specs., Irebu 30-XII-1920 D^r^H.Schouteden MUSÉE DU CONGO (MRACT, CBB); 10 specs., COLL. MUS. Congo Ubangi: Nouvelle-Anvers 9-XII-1952 P. Basilewsky / A la lumière (MRACT, CBB); 1 spec., I.R.S.A.-MUS.R.A.C. Equateur : terr. Bikoro, Mabali IX.1959 N. Leleup / Forêt inondée à la lumière (MRACT); 1 spec., Coquilhatville – a table le soir – G Juillet 1909 / MUSÉE DU CONGO Voyage de S.A.R. le Prince Albert 1909 (MRACT); 1 spec., MUSÉE DU CONGO Stanleyville a Coquilhatville Dr. Géranrd. (MRACT); 1 spec., MUSÉE DU CONGO Kai Bumba D^r^H.Schouteden 11-X-1920 (MRACT); 1 spec., COLL. MUS. CONGO Tshuapa : Bamania XII-1952 R. P. Hulstaert (MRACT); 1 spec., Congo Belge, P.N.G. Miss. H. De Saeger Mabanga 1949–1952 Réc. H. De Saeger (MRACT); 1 spec., Congo belge, P.N.G. Miss. H. De Saeger I/b/3’’, Mabanga 14-IV-1950 Réc. H. De Saeger.458 / Salcedia (MRACT); 1 spec., MUSEUM PARIS Afrique Équatoriale Fr. BRAZZAVILLE ANDRÉ GIDE 1925 / AOUT / Salcedia coquilhati Alluaud det. 1934 (MNHN).

#### Diagnosis.

A small sized species, with long-ovoid outline of the elytra with maximum width at posterior third. The pseudohumerus is obtuse angled and not dentate. The antennomeres are elongate. Distinguished from all other species of the genus by the extraordinary transverse pronotum which shows at the lateral margin five large transverse pits instead of six pits present in all other species, and the head exhibits a reniform outline in dorsal view.

#### Redescription.

Measurements in Table 1.

***Colour and surface:*** Melaneous, opaque; top of carinae griseous; mandibles and legs fuscous, antennae and palpi hinnuleous.

***Head:*** Two-thirds of width of pronotum. Outline reniform. Clypeus wide, slightly but distinctly convex anteriorly, fused with clypeal wings, separated from supra-antennal plates by obtuse notches, frons with two flattened paramedian carinae, joining anteriorly into a convex bulge not clearly separated from frons, with two small glossy teeth bilaterally anterior to central bulge, with two parallel carinae paralaterally near base; frons and clypeus separated from supra-antennal and supraorbital plates by deep broad furrows, each furrow with moderately deep circular pit between supra-antennal plate and clypeus; supra-antennal and supraorbital plates distinctly margined, supra-antennal plates slightly vaulted, margin of supraorbital plates conspicuously raised. Base emarginated at middle, laterally rounded-off and constricted to neck without angle. Eyes large, convex, genae slightly convex, both of them clearly visible from above, with nearly circular shape in lateral view. Antenna with segments five to ten elongate (L/W 1.26), densely pubescent, segments two to four scarcely pubescent, scapus with minute longitudinal reticulation. Labrum straight anteriorly. Mandible moderately short, wide, slightly arcuate at apex. Mentum small, epilobes wide, projecting and nearly rectangular anteriorly, completely margined, surface coriaciate-like with flat impressions.

***Pronotum*** (Fig. 29): Outline rectangular, conspicuously transverse, nearly half times wider than long. Lateral margin straight but converging anteriorly, maximum width at posterior third, slightly rounded anteriorly and to posterior angles. Lateral margin distinctly crenulated, with nine tubercles, with distinct emargination at posterior angles. Base with wide tubercle laterally. Disc with two raised paramedian irregularly sinuate carinae parallel to median line, joining at anterior margin, with median line indistinct, with two additional shorter carinae bilaterally, the paralateral one joining with the paramedian carinae at base and forming tooth-like tubercle pointing posteriorly, extended anteriorly as less raised paralateral carina. With one distinctly long outer lateral carina running up to anterior margin. Lateral margin and space between lateral margin and lateral carina wing-like bent up, with five large transverse pits.

***Elytron:*** Slightly convex in anterior half (lateral view), convex in frontal view. Outline in posterior five sixths long-ovoid, maximum width at middle. Pseudohumerus sub-rectangular, with small tubercle-like tooth. Apex acute at suture. Disc with interneur six crenulated, interneur two running up to apex as distinctly convex line, conspicuously raised; interneur four running in parallel to interneur six, reaching base, not reaching apex. Interneur four and six with two to three indistinct rows of pits becoming transverse apically.

***Hind wings:*** Fully developed.

***Lower surface:*** Pronotum with surface of antennal channel with irregular reticulation. Pseudoepipleura with two rows of serial pits, lateral margin of elytron smooth. Metepisternum elongate, with broad longitudinal groove. Metasternum and last five abdominal sternites with numerous pits, regularly arranged at carinae and margins. Sternum four to six distinctly sulcate.

***Legs:*** Profemora with dorsal surface indistinctly reticulated. Protibia with terminal spine arcuate at apex, laterally with three teeth, dorsally and ventrally with two carinae. Movable spur arcuate, acute, length nearly as half as first tarsomere. First tarsomeres distinctly elongated, almost as long as tarsomeres two to five together.

***External sexual dimorphism:*** Not observed.

***Male genitalia*** (Fig. 55): Median lobe short, stout, in dorsal view straight, in lateral view straight, with a few fine pili in apical third, oroficium small, apex circular in cross-section, slightly turned dorsally. Endophallus with two elongated groups of microtrichia, groups as long as one fifth of median lobe. Dorsal paramere sinuate in apical half, lateral and basal apophysis slender; both parameres slightly distorted.

***Female genitalia*** (Fig. 70): Coxostylus less sclerotised, broadened just anterior base, distinctly curved dorsally and slightly medially, slender in apical third, at end of basal third with one shorter strong and three slender nematiform setae laterally, with another slender seta at middle, with one long strong nematiform seta ventrally.

***Variation:*** On the elytron between the interneurs four and six, the rows of pits vary in the number of pits and rows. In some of the specimens the labrum is indistinctly convex.

#### Distribution

(Fig. 84). Known from several localities along the course of the Congo River and at the mouth of the river Tshuapa. Some of the specimens were collected at light. The historic name Coquilhatville refers to the city today called Mbandaka, that of Stanleyville to Kisangani, and Brazzaville to Kinshasa.

### Salcedia elongata

Taxon classificationAnimaliaColeopteraCarabidae

Alluaud, 1932

553B9670-AD03-5754-888D-7EDABC5968D3

Figs 6a , 12 , 30 , 46 , 56 , 71 , 84

Salcedia elongata Alluaud, 1932: 3; Burgeon 1935: 159; Csiki 1933: 641; Reichardt 1975: 103; Dostal 1993: 121; 1932, Lorenz 2005: 155.

#### Type material.

***Holotype:*** ♂, with labels and data: white, printed and handwritten “MUSÉE DU CONGO Katanga: Lufira 13-IV-1925 Ch. Seydel” / red, glued “TYPE” / white printed and handwritten “R. DÉT. 2019 ol” / white, black framed, handwritten and printed “*Salcedia elongata* All. Alluaud det. 1931” / red, black framed “HOLOTYPUS” (MRACT). ***Paratypes***: 1 spec., same data as holotype but “R. DÉT. 2019 e” and red, black double framed “PARATYPE”; 1 spec., same data but “MUSÉE DU CONGO Katanga: Kangele 4-IV-1925 Ch. Seydel” (MRACT).

#### Remark.

In the paratype from Lufira the pronotum and head is missing.

#### Additional material.

2 ♂, 1 ♀, 4 specs., “COLL. MUS. CONGO Elisabethville –X–1940 H.J. Brédo” (MRACT); 5 specs., “COLL. MUS. CONGO Elisabethville II/V-1949 Ch. Seydel” / black framed “A la lumière” (MRACT); 6 specs., “COLL. MUS. CONGO Elisabethville (lumière) XI. 1951 – II. 1952 Ch. Seydel” (MRACT, CBB); 6 specs., “COLL. MUS. CONGO Elisabethville (a la lumière) 1.III-52/30-IX-1953 Ch. Seydel” (MRACT); 4 specs., “COLL. MUS. CONGO Elisabethville (a la lumière) 1953/1955 Ch. Seydel” (MRACT); 28 specs., “COLL. MUS. CONGO Elisabethville, A la lumière XI-50/VI-51 Ch. Seydel” (MRACT, CBB); 1 spec., “COLL. MUS. CONGO Elisabethville IX-1958/V-1959 Ch. Seydel” (MRACT); 1 spec., “MUSÉE DU CONGO Katanga: Lufira 13-IV-1925 Ch. Seydel” (MRACT). 1 spec. “COLL. MUS. CONGO Elisabethville (à la lumière) XII-1952 H. Bomans” (MRACT); 2 ♀, “COLL. MUS. CONGO Tanganika: Musosa, 980 m. (a la lumière) XI-1953 H. Bomans” (MRACT); 1 ♀, 2 specs., “I.R.S.A.C.-MUS. CONGO Kivu : Kavimvira (Uvira) (a la lumière) II/III-1955 G. Marlier” (MRACT); 2 specs., MUS. ROY. AFR. CENTR. Lualaba : Zilo (écorces) 124 13-II-1960 Dr. V. Allard (MRACT); 4 specs., COLL. MUS. CONGO Lulua : Kapanga V/VII-1958, IV/IX-1958, V/VII-1959, V/VII-1959, J. Allaer (MRACT, CBB); 1 ♂, 3 ♀, 1 spec., Congo Belge, P.N.G. Miss. H. De Saeger Mabanga, 25-III-1952 H. De Saeger. 3220; 3411; 3114;3339; 3883 (MRACT, CBB); 2 ♀, Congo Belge, P.N.G. Miss. H. De Saeger Pidigala, 24-IV-1952 H. De Saeger. 3325 (MRACT); 1 ♀, Soil-Zoological Congo-Brazzaville Kindamba,Méya settlement / 9.11.1963, No 147 by lamplight leg.Endrödi-Younga (CBP); 1 ♂, 1 ♀, 4 specs., ZAMBIA NORTHERN PROVINCE 15 km NE LUWINGU 03.-04.2008 1400 M A.KUDRNA JR LGT (CBP); 1 ♀, ZAMBIA c., Northern pr. CHIPONA FALLS, 5–8.xiI 30 km S of Chinsali leg. F. + L. Kantner 2002 (CBP); 1 ♀, Angola : Nzargi-Andrada VIII/XII-1952 A. de Barros Machado (MRACT); 1 ♂, 3 specs., UGANDA NC. 20km NE of GULU PATIKO env. 5.12.2001 Lgt.M.SNIŽEK (CBP);

#### Diagnosis.

A large species, with oblong-elongate parallel outline of the elytra with maximum width at middle and the pronotum with three additional carinae of which the inner and outer lateral carinae are distinctly shortened. The pseudohumerus is nearly rectangular with indistinct tooth. The antennomeres are moniliform. Distinguished most clearly from the similar species *S. africana* by the pronotum with anteriorly projecting angles and with shortened inner and outer lateral carinae. Moreover, in *S. africana* the antennomeres are sub-elongate, interneur four of the elytron is running up to the base, and the front tibia shows five lateral teeth instead of four. The second similar species, *S. procera* sp. nov. is much smaller, its antennomeres are sub-moniliform, and the elytra are super-elongate with the lateral outline slightly concave.

#### Redescription.

Measurements in Table 1.

***Colour and surface:*** Piceous, areas between carinae and pits shiny; lateral fifth of pronotum translucent fuscous; mandibles, legs, and antennae fuscous, palpi leoninous.

***Head:*** Four-fifths of the pronotum width. Outline pentagonal shaped. Clypeus wide, straight anteriorly, fused with clypeal wings, separated from supra-antennal plates by distinct notches, with convex field at middle, separated from frons by deep transverse furrow. Frons with two raised paramedian carinae, joining anteriorly, prolonged anteriorly into a central distinctly erected tubercle, with two small rounded glossy teeth bilaterally anterior to central tubercle, with two parallel carinae paralaterally near base; frons and clypeus separated from supra-antennal and supraorbital plates by deep broad furrows, each furrow with deep slightly longitudinal pit between supra-antennal plate and clypeus; supra-antennal and supraorbital plates distinctly margined, margin raised, carina-like, indistinctly crenulated, supra-antennal plates slightly vaulted. Base emarginated at middle, distinctly but obtuse angled laterally (angle 135–136°). Eyes large, convex, with transverse-pentagonal shape in lateral view, with small part just visible in dorsal view; genae slightly convex. Antenna with segments five to ten moniliform (L/W 1.02), densely pubescent, segments two to four scarcely pubescent, scapus, and pedicellus with longitudinal reticulation. Labrum slightly pointed anteriorly. Mandible moderately short, wide, slightly arcuate at apex. Apical segment of maxillary palpomere moderately long. Mentum small, epilobes wide, projecting and distinctly angled anteriorly, completely margined, surface covered with pits.

***Pronotum*** (Fig. 30): Outline rectangular, transverse, a quarter wider than long. Lateral margin straight and parallel at middle, maximum width at middle, slightly converging at anterior and posterior angles. Lateral margin distinctly crenulated, with twelve to thirteen tubercles, with anterior angles right-angled projecting, with moderate emargination at posterior angles. Base straight laterally, without keel at middle. Disc with two raised paramedian carinae parallel to median line and diverging posteriorly, with few slight notches, with median line narrow but longer and ending in pits anteriorly and posteriorly, with four additional shorter carinae bilaterally, the paralateral ones joining with the paramedian carinae at base and forming tooth-like tubercle pointing posteriorly, extended anteriorly as less raised paralateral carina. With two very short and less raised inner and outer lateral carinae, the inner one connecting the paralateral with the small outer lateral carina. All carinae sub-crenulate. Lateral margin and space between lateral margin and paralateral carina wing-like bent up, with six large transverse pits.

***Elytron*** (Fig. 46): Flattened in anterior half (lateral view), regularly convex in frontal view. Oblong-elongate, margin straight, parallel, maximum width at middle. Pseudohumerus nearly rectangular, indistinctly dentate. Apex rounded, acutely and small denticulate at suture. Disc with interneur six crenulated, interneur two running up to apex as parallel line, conspicuously raised; interneur four in parallel at middle, convex towards apex, not reaching apex, nearly reaching base. Interneur five nearly not visible due to transversally connected pits. Pits approaching very close to interneur six.

***Hind wings:*** Fully developed.

***Lower surface*** (Fig. 6a): Antennal channel of pronotum with isodiametric reticulation. Pseudoepipleura with two rows of serial pits, one of the rows becoming indistinct apically, lateral margin of elytron smooth. Metepisternum distinctly elongated, with broad longitudinal groove. Last visible sternum with longitudinal carina laterally and a longitudinal blunt carina at middle.

***Legs:*** Profemora dorsally with a pit and isodiametric reticulation. Protibia stout, with short moderately curved terminal spine, laterally with four teeth of decreasing size, dorsally and ventrally with two carinae. Movable spur short, length a quarter of first tarsomere. Meso- and metafemora of moderate length, slender, slightly dilated basally. First tarsomeres distinctly elongated, almost as long as tarsomeres two to five together.

***External sexual dimorphism:*** The longitudinal central blunt carina at middle of sternum six is more distinctly developed in females.

***Male genitalia*** (Fig. 56): Median lobe elongated, slender, in dorsal view moderately arcuate in basal two thirds, distinctly bent at beginning of apical third, apex straight, in lateral view bisinuate, with fine pili at middle to apical third, apex stick-like in cross section sub-ovoid, round directly at apex. Oroficium large. Endophallus with two groups of microtrichia, a broader elongated one with length of one fifth of median lobe, a second shorter group at the opposite side. Dorsal parameres long, slender, sinuate, with lateral and basal apophysis narrow, slightly distorted; ventral one inconspicuous.

***Female genitalia*** (Fig. 71): Coxostylus broadened, distinctly curved, with distinct carina dorsally in apical third, at end of basal third with one fine small seta, one strong and five slender nematiform setae laterally, SSO with two microtrichia.

***Variation:*** In some of the specimens the elytra are indistinctly depressed in anterior half in lateral view. Protibia in some specimens with five teeth laterally. In specimens from Luwingu (Zambia) the lateral margin of the elytron is not exactly straight but indistinctly curved and the lateral margin of the pronotum shows eleven to twelve tubercles.

#### Distribution

(Fig. 84). The species occurs in the south-west and the south of the Democratic Republic of the Congo and in the north of Uganda. Finds were also made in the north of the Democratic Republic of the Congo at the Lufira and Luabala River. Many specimens were collected at light. The historic name Elisabethville refers to the city today called Lubumbashi, that of Brazzaville to Kinshasa.

### Salcedia africana

Taxon classificationAnimaliaColeopteraCarabidae

(Britton, 1947)

3DA2BF1D-5255-51E9-8E77-ADE2617A7375

Figs 13 , 31 , 47 , 57 , 72 , 84

Zelma africana Britton, 1947: 126.
Salcedia africana (Britton, 1947): Reichardt 1975: 103; Dostal 1993: 121; Lorenz 2005: 155.

#### Type material.

**Holotype**: ♀, with labels and data: white, printed, with red line “N.Rhodesia: N’Changa. C.T.Macnamara. B.M.1931-179.” / white, handwritten “HOLOTYPE. Zelma Africana Britton” / white, circle, black printed, with red circle “Type” (BMNH).

#### Remark.

In the holotype, the following parts are missing: Mentum and half of the mouthparts including glossa, ligula and mentum, right antennae from joint eight onwards, parts of left front tibia and all left front tarsomeres, right meso tarsomeres. Head, pronotum and hind body were glued.

#### Additional material.

1 ♀, ZAMBIA LUSAKA PROVINCE 30 km S LUSAKA 16.-17.12.2002 A. KUDRNA JR. LGT. (CBB); 1 ♀, ZAMBIA CENTRAL PROVINCE 15 km S KAPIRI MPOSHI 30.11.2002 A. KUDRNA JR. LGT. (CBB); 1 ♀, Zambia, Copperbelt Prov. 60 km SE of Kitwe, 13.7.2002 F. & L. Kantner lgt. (SMNS); 1 ♂, 2 ♀, 1 spec., ZAMBIA COPPERBELT 50 km W CHINGOLA 31.12.02-2.1.03 A. KUDRNA JR. LGT. (CBB); 2 specs., ZAMBIA COPPERBELT 45 km SE KITWE 12.–15.01.2003 A. KUDRNA JR. LGT. (CBB); 1 ♂, 1 spec., ZAMBIA: CENTRAL PR. 6.5 km N Chunga, 1100 m, 14°59'40"S, 26°01'11"E 4.XII.2010, open central Zambezian & Miombo woodland light trap, F. Génier, 2010-43 (CBP); 1 spec., ZAMBIA 30.xi.2002 KAPIRI MPOSHI env. Central prov. F. & L. Kantner leg. (CBP); 2 ♂, ZAMBIA 19./30.XI.14NW Province Chimfunsi Wildlifereserve 12°21.762'S, 27°30.911'E P. Schüle leg. (CBP); 2 ♂, ZAMBIA C. 40 km N Kabwe Sungala school env. 22.11.2004 Snižek, Tichý lgt. / Coll. A. DOSTAL (CDW); 1 ♂, 2 ♀, 12 specs., N.RHODESIA: Mwinilunga District, Ikelenge, nr. Kalene Zambetsi Rapids. / E.Pinhey. 3.v.1963. M.V. Light trap. B.M.1963-742.; 1 spec. with add. label “*Salcedia africana* (Britt.) J. Balfour-Brown det. 1963” (BMNH, CBB); 2 ♂, ZAMBIA 1346 m Kambishi School 11°45'42"S, 25°28'50"E 10–13.xi2017. Actinic Light Trap. / Carter,M., Lloyd,A., Miles,W., Oram,D., Smith,R. leg. ANHRT: 2017:27, ZM-011 BMNH(E) 2017-194 / label with barcode and NHMUK013685510 (BMNH); 1 spec., MUSEUM PARIS ZAMBÈZE NOVA CHOUPANGA PRÈS CHEMBA P. LESNE 1929 (MNHN).

#### Diagnosis.

A large species, with oblong-elongate nearly parallel outline of the elytra with maximum width at middle and the pronotum with three complete additional carinae. The pseudohumerus is slightly obtuse angular and distinctly dentate. The antennomeres are super-moniliform. Distinguished most clearly from the similar species *S. elongata* by the pronotum with convex lateral margin and angles not projecting anteriorly, and with complete inner and outer lateral carinae. Moreover, in *S. elongata* the antennomeres are moniliform, interneur four of the elytron is somewhat shortened at base, and the front tibia shows four lateral teeth. The second similar species, *S. procera* sp. nov. is much smaller, its antennomeres are sub-moniliform, and the elytra are super-elongate with the lateral outline slightly concave.

#### Redescription.

Measurements in Table 1.

***Colour and surface:*** Piceous, areas between carinae and pits shiny; lateral fifth of pronotum fuscous; mandibles and legs piceous, antennae hinnuleous, palpi leoninous.

***Head:*** Three-quarters of pronotum width. Outline shaped like a frustum of a pyramid. Clypeus wide, straight anteriorly, fused with clypeal wings, separated from supra-antennal plates by distinct notches, with transverse elevated field at middle, separated from frons by deep transverse furrow. Frons with two raised paramedian carinae, joining anteriorly, prolonged anteriorly into a central small but distinctly erected tubercle, with two small rectangular glossy teeth bilaterally anterior to central tubercle, with two parallel carinae paralaterally near base; frons and clypeus separated from supra-antennal and supraorbital plates by deep broad furrows, each furrow with deep longitudinal pit between supra-antennal plate and clypeus; supra-antennal and supraorbital plates distinctly margined, margin raised, carina-like, at supraorbital plates indistinctly crenulated, supra-antennal plates slightly vaulted, surface of supraorbital and supra-antennal plates with numerous small flat irregular impressions. Basal border emarginated at middle, distinctly and obtuse angled laterally (angle 118–119°). Eyes convex, with transverse-rectangular shape in lateral view, with small part clearly visible in dorsal view; genae slightly convex, with small indistinct notch anterior angle. Antenna with segments five to ten super-moniliform (L/W 1.1), densely pubescent, segments two to four scarcely pubescent, scapus, and pedicellus with longitudinal reticulation. Labrum clearly visible from above, convex anteriorly. Mandible moderately short, wide, slightly arcuate at apex. Epilobe of mentum wide, projecting and distinctly angled anteriorly, completely margined, surface covered with pits.

***Pronotum*** (Fig. 31): Outline rectangular, transverse, one third wider than long. Lateral margin almost straight, slightly converging at anterior angles, maximum width at middle. Lateral margin distinctly crenulated, with twelve tubercles, with moderate emargination at posterior angles. Base straight laterally, with broad flat keel at middle. Disc, with two raised paramedian carinae parallel to median line, sinuate and diverging posteriorly, interrupted by notches, with median line small but longer and ending in pits anteriorly and posteriorly, with four additional shorter carinae bilaterally, the paralateral one joining with the paramedian carinae at base and forming tooth-like tubercle pointing posteriorly, extended anteriorly as distinctly raised paralateral carina. With shorter inner lateral carina and quite long outer lateral carina. Carinae nearly smooth on top. Lateral margin and space between lateral margin and paralateral carina wing-like bent up, with six large transverse pits.

***Elytron*** (Fig. 47): Indistinctly depressed in anterior half (lateral view), regularly convex in frontal view. Oblong-elongate, margin straight, parallel in anterior two thirds, maximum width at middle. Pseudohumerus slightly obtuse angular, distinctly dentate. Apex long-oval, edenticulate. Disc with interneur six indistinctly crenulated, interneur two running up to apical margin as parallel line, conspicuously raised; interneur four running in parallel at middle, slightly convex towards apex, not reaching apex, nearly reaching base.

***Hind wings:*** Fully developed.

***Lower surface:*** Antennal channel of pronotum with isodiametric reticulation. Pseudoepipleura with rows of indistinct flat hardly recognisable pits, lateral margin of elytron smooth. Metepisternum distinctly elongated, with broad longitudinal groove. Last visible sternum with acute longitudinal carina at middle, hollowed out laterally.

***Legs:*** Profemora with dorsal surface indistinctly irregularly reticulated. Protibia longer, with terminal spine curved at apex, laterally with five teeth of decreasing size, dorsally and ventrally with two carinae. Movable spur short, length a third of first tarsomere, curved ventrally. Meso- and metafemora of moderate length, slender. First tarsomeres distinctly elongated, almost as long as tarsomeres two to five together.

***External sexual dimorphism:*** Not observed.

**Male genitalia** (Fig. 57): Median lobe elongated, slender, in dorsal view regularly arcuate from base nearly up to apex, in lateral view straight at middle, sinuate at base and apex, with fine scattered pili in middle, apex spatulate, in cross section convex at apex, convexity directed ventrally. Oroficium of moderate size. Endophallus with two shorter groups of microtrichia, the broader one with more robust trichia, a second shorter group at the opposite side. Dorsal paramere long, slender, bisinuate, slightly distorted, with apophyses narrow; ventral one small, stub-like.

***Female genitalia*** (Fig. 72): Coxostylus robust, distinctly curved, with distinct carina dorsally in apical third, with obtuse hook-like apex somewhat rounded, at end of basal third with one large nematiform seta and six slender nematiform setae laterally, SSO with two microtrichia.

***Variation:*** In a few specimens from Zambia, the outlines of the elytra are not exactly straight but show an indistinct convexity.

#### Distribution

(Fig. 84). The holotype was found in the N’Changa district in northern Zimbabwe, formerly Rhodesia. In addition, the species is known from localities in Zambia (Central Province up to Lusaka) at the upper and lower course of the Zambezi River.

### Salcedia schoutedeni

Taxon classificationAnimaliaColeopteraCarabidae

Alluaud, 1930

5DEE114B-7FB4-5465-93D6-7C204D8440B6

**Figures 14–19.**
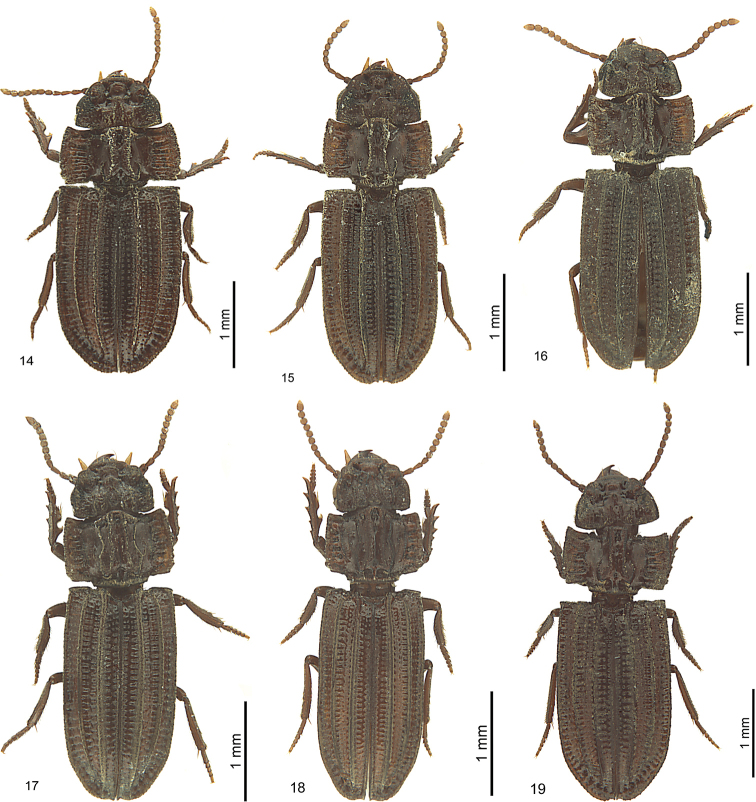
*Salcedia* species, habitus, dorsal view, holotype **14***S. schoutedeni* Alluaud **15***S. nigeriensis* Alluaud (cotype) **16***S. putzeysi* (Oberthür) **17***S. matsumotoi* sp. nov. **18***S. procera* sp. nov. **19***S. baroensis* sp. nov.

Figs 14 , 32 , 48 , 58 , 73 , 84

Salcedia schoutedeni Alluaud, 1930: 21; Alluaud 1932: 2; Csiki 1933: 641; Alluaud 1935: 19; Burgeon 1935: 159; Andrewes 1936: 64.
Salcedia schoutedeni schoutedeni Alluaud, 1930: Reichardt 1975: 103; Dostal 1993: 121; Lorenz 2005: 155.

#### Type material.

**Holotype**: ♀, with labels and data: white, printed and handwritten “MUSÉE DU CONGO Kwamouth 9-VII-1921 Dr H.Schouteden” / white, printed black framed “R. DÉT. 1783 c” red, black double framed “HOLOTYPE” / white, black framed, handwritten and printed “*Salcedia Schoutedeni* Type Alluaud, det. 1930” (MRACT).

#### Remark.

In the holotype both of the front legs are broken and glued.

#### Additional material.

1 ♂, 1 ♀, 22 specs., Léopoldville I-1947 Dr E. Dartevelle COLL. MUS. CONGO (MRACT, CBB); 1 ♀, same data (MNHN); 1 spec., Léopoldville -1930 Eg. Devroye MUSÉE DU CONGO / R. DÉT. 2019 b / *Salcedia schoutedeni* All. Alluaud. det. 1931 (MRACT); 1 spec., Léopoldville -1930 E. Devroey MUSÉE DU CONGO (MRACT); 1 spec., same data but -1931 (MRACT).

#### Diagnosis.

A large sized species, with sub-elongate outline of the elytra with maximum width at middle and the pronotum with a rudiment of the outer lateral carina. The pseudohumerus is rectangular with a distinctly laterally projecting tooth. The antennomeres are elongate. Distinguished most clearly from the similar species *S. nigeriensis* by the pronotum with the lateral margin converging from middle to the anterior angles, the pseudohumerus of the elytron with a distinct tooth, and the female coxostylus with six large setae and one SSO. In addition, *S. schoutedeni* has a distinct keel on the frons of the head.

#### Redescription.

Measurements in Table 1.

***Colour and surface:*** Anthracite grey, shiny; top of carinae on head, pronotum and elytra as well as margins of pronotum and elytra opaque, covered with pale grey pili; legs piceous, mandibles, antennae and palpi piceous.

***Head:*** Less than two-thirds of width of pronotum. Outline semi-circular. Clypeus wide, convex anteriorly, fused with clypeal wings, separated from supra-antennal plates by distinct notches, with slightly raised pentagon-shaped field at middle, separated from frons by deep transverse furrow. Frons with two raised paramedian carinae, joining anteriorly into a V-like keel, with two small glossy teeth bilaterally anterior to central keel, with two indistinct short parallel carinae paralaterally at base; frons separated from supraorbital plates by deep broad furrows; with conspicuously deep and broad pit at front-eye level; supra-antennal and supraorbital plates margined, margin of supraorbital plate distinctly raised, carina-like, supra-antennal plates vaulted. Base with narrow emargination at middle, sharply rectangular laterally (angle around 109°). Eyes large, convex, genae slightly convex, both of them clearly visible from above, with nearly circular shape in lateral view. Antenna with segments five to ten elongate (L/W 1.25), densely pubescent, segments two to four scarcely pubescent, scapus with irregular reticulation. Labrum nearly straight anteriorly. Mandible moderately short, wide, slightly arcuate at apex. Mentum small, with slight tooth at middle, epilobes wide, projecting and angled anteriorly, margined anteriorly, surface with small pits anteriorly, with isodiametric reticulation basally.

***Pronotum*** (Fig. 32): Outline rectangular, transverse, two-fifth wider than long. Lateral margin convex, distinctly converging anteriorly, maximum width behind middle. Lateral margin crenulated, with 12–13 small tubercles, tubercles larger basally, with two notches at posterior angles. Base straight laterally, with declining flat keel at middle pointing posteriorly. Disc flattened in lateral view, with two raised paramedian carinae parallel to median line and diverging posteriorly, with median line small, long, and ending in pits anteriorly and posteriorly, with two additional shorter carinae bilaterally, the paralateral one joining with the paramedian carinae at base and forming tooth-like tubercle pointing posteriorly, without anterior extension, without inner lateral carina, with very small outer lateral carinae. All carinae sub-crenulate. Lateral margin broadly wing-like bent up, with six large and deep transverse pits, the basal and/or frontal ones partly separated into two smaller pits. Space between carinae and pits smooth.

***Elytron*** (Fig. 48): Flattened in anterior half (lateral view), convex in frontal view. Sub-elongate, margin straight in anterior half up to pseudohumerus, moderately convex to apex, maximum width at middle, sub-undulate. Pseudohumerus rectangular, tooth distinctly projecting laterally. Apex rounded, with small but acute tooth at suture. Disc with interneur six sub-crenulate, interneur three slightly carinate in basal third, interneur two running up to apex as slightly convex line, conspicuously raised; interneur four running in parallel to interneur six, almost reaching base, shortened at apex. Interneur five and six with two rows of serial pits partly merging transversally.

***Hind wings:*** Fully developed.

***Lower surface:*** Antennal channel of pronotum with isodiametric reticulation. Pseudoepipleura with one row of slightly transverse pits, lateral margin of elytron smooth. Metepisternum elongate, with broad longitudinal groove. Metasternum, abdominal sternites one and five with numerous irregularly situated larger and smaller pits, sternite two to three smooth, with band of small punctures at middle. Last three abdominal sternites laterally with isodiametric reticulation, sternite one with longitudinal reticulation, two with transverse reticulation. Sternum four to six slightly sulcate. Sternum six at apical margin with hollowed out transverse impression at apex.

***Legs:*** Profemora with surface indistinctly reticulated. Protibia with short, robust, moderately curved terminal spine, laterally with laterally with two larger and two smaller teeth, dorsally and ventrally with two carinae. First tarsomere distinctly elongated, as long as tarsomeres two to five together, front and intermediate legs with tarsomeres two to four broadened and somewhat flattened.

***External sexual dimorphism:*** Sternum six with the hollowed out transverse impression at the apical margin is obtusely longitudinally sulcate in females.

***Male genitalia*** (Fig. 58): Median lobe stout, in dorsal view distinctly and regularly arcuate, straight to apex, in lateral view straight, apparently no fine pili visible, apical part bent ventrally apex dorsally, in cross section oval at apex. Oroficium small. Endophallus with group of longer microtrichia near oroficium, with additional longer group of brush-like microtrichia basally. Dorsal paramere relatively short, bisinuate, with short apophyses; ventral one shaped like a convex spatula, with fine seta at apex; both parameres slightly distorted.

***Female genitalia*** (Fig. 73): Coxostylus regularly broadened to base, distinctly curved, acute at apex, with indistinct carina dorsally in apical third, at end of basal third with one strong and five slender nematiform setae laterally, SSO with one fine pilus.

***Variation:*** The tubercles at the lateral margin of the pronotum are more or less strongly developed. Number and shape of the pits on the ventral surface varies to a small degree.

#### Distribution

(Fig. 84). Known from several localities in the west of the Democratic Republic of the Congo along the lower course of the river Congo. The locality Kwamouth refers to the short estuary of the river Kasai into the River Congo. The location “Zambèze, Nova Chupanga près Chemba” quoted in Alluaud (1935) as new record for *S. schoutedeni* refers to *Salcedia africana*.

### Salcedia nigeriensis

Taxon classificationAnimaliaColeopteraCarabidae

Alluaud, 1932

C1C48FBD-2BFA-5BAE-B32E-68DD362ADB2D

Figs 15 , 33 , 49 , 59 , 74 , 84

Salcedia schoutedeni ssp. nigeriensis Alluaud, 1932: 2; Salcedia schoutedeni var. nigeriensis Alluaud, 1932, Csiki 1933: 641; Salcedia schoutedeni nigeriensis Alluaud, 1932, Jeannel 1946: 233.
Salcedia nigeriense [sic !] Alluaud, 1932: Basilewsky 1973: 293.
Salcedia schoutedeni nigeriensis Alluaud, 1932: Reichardt 1975: 103.
Salcedia nigeriense [sic !] Alluaud, 1932: Dostal 1993: 121.
Salcedia nigeriensis Alluaud, 1930 [sic !]: Lorenz 2005: 155.

#### Type material.

**Paratypes**: 1 ♂, with labels and data: white, handwritten “Tillabéry Niger Alluaud XII.1930 h° 8.” / white, “R. DÉT. 2019 c” / white, black framed, handwritten and printed “Schoutedeni ssp. nigeriensis All. Alluaud det. 1931” / red, black framed, printed “PARATYPE” / white, printed “COLL. MUS. CONGO” (MRACT); 1 ♀, same data but printed, handwritten “Tillabéry, Niger Ch. Alluaud, XII.1930” (MRACT); 1 spec., same data as before (MRACT); 3 specs. mounted on one card, one without head, with labels and data: white, handwritten in black ink “Gao bords du Niger I. 1931 (Alluaud)” / “Salcedia schoutedeni Subsp. nigeriensis Cotypes All. Alluaud det.” (MNHN); 2 specs. mounted on one card, with labels and data: handwritten in black ink “Gao bords Niger no. 7” / white with red line: “co-type” / “white, handwritten and printed, black framed: “*Salcedia schoutedeni* v. *nigeriensis* Alluaud det. 1931” (MNHN); white, handwritten in black ink: “Tillabery Alluaud XII 1930” / “Salcedia schoutedeni ssp. nigeriensis Alluaud cotypes” / blue, printed “MUSEUM PARIS 1932 Ch Alluaud” (MNHN).

#### Additional material.

16 specs., SOUDAN FRANÇAIS Gao / MUSÉUM PARIS 12 – 1930 – VI – 1931 Ch. Alluaud & P. A. CHAPPUIS (MNHN, CBB); 1 spec., same data but add. label with: *Salcedia nigeriense* All. P. Basilewsky det., 19 “ / COLL. MUS. CONGO Coll. P. Basilewsky (MRACT); 1 spec., Bords Niger près Ansongo –I–1931 (Ch. Alluaud) MUSÉE DU CONGO / R. DÉT. 1944 E (MRACT); 1 ♂, Soudan franç.: Gao Alluaud – Chappuis; backside: don R. Jeannel, Jeannel Coll. P. Basilewsky / COLL. MUS. CONGO Coll. P. Basilewsky (MRACT);

#### Taxonomic remarks.

The holotype should be deposited in MNHN (Alluaud 1932) but could not be located. However, there were seven specimens labelled by Alluaud as “Co-types” among the 23 specimens deposited in MNHN. The species was originally described as subspecies of *Salcedia schoutedeni* by Alluaud, 1932 from Gao. The labels of the type material with handwriting of Alluaud also indicate “Schoutedeni ssp. nigeriensis Alluaud”. However, Basilewsky (1973) listed it as a species “*Salcedia nigeriense* Alluaud” without further comment. The material used by Basilewsky for his study contains a mixture of paratypes from Alluaud and material determined by Basilewsky which is labelled “*Salcedia nigeriense* All.” in Basilewsky’s handwriting. Examination revealed all specimens are conspecific. So, it is presumed that “nigeriense” is a writing error by Basilewsky.

**Diagnosis.** A large sized species, with sub-elongate outline of the elytra with maximum width at middle and the pronotum with a short outer lateral carina near base. The pseudohumerus is rectangular and without distinctly projecting tooth. The antennomeres are elongate. Distinguished most clearly from the similar species *S. schoutedeni* by the pronotum with the lateral margin slightly convex but not converging anteriorly, the pseudohumerus of the elytron without distinctly projecting tooth, and the female coxostylus with eight large setae and without SSO. In addition, *S. nigeriensis* shows a distinctly erected tubercle on the frons of the head.

#### Redescription.

Measurements in Table 1.

***Colour and surface:*** Fuscous to griseous, shiny; top of carinae on head, pronotum and elytra as well as margins of pronotum and elytra opaque, covered with pale grey pili; legs and mandibles fuscous, antennae and palpi leoninous.

***Head:*** Four-fifths of the pronotum width. Outline semi-circular. Clypeus wide, straight anteriorly, fused with clypeal wings, separated from supra-antennal plates by obtuse notches, with raised transverse field at middle, separated from frons by broad flattened shiny transverse furrow, frons with two raised paramedian carinae, joining anteriorly into a V-like keel, prolonged anteriorly into a central small but distinctly erected tubercle, with two small glossy teeth bilaterally anterior to central keel, with two indistinct short diverging carinae paralaterally at base. Frons separated from supraorbital plates by flattened broad furrows; with conspicuously deep and broad pit at front-eye level; supra-antennal and supraorbital plates margined, margin of supraorbital plate distinctly raised, carina-like, supra-antennal plates slightly vaulted. Basal border with narrow emargination at middle, nearly rectangular laterally (angle 96–98°). Eyes large, convex, genae slightly convex, both of them clearly visible from above, with ovoid shape in lateral view. Antenna with antennomeres five to ten elongate (L/W 1.24), densely pubescent, segments two to four scarcely pubescent, scapus with sub-elongate reticulation. Labrum slightly convex anteriorly. Mandible moderately short, wide, slightly arcuate at apex. Mentum small, without tooth at middle, epilobes wide, projecting and angled anteriorly, nearly completely margined, surface with indistinct pits, with isodiametric reticulation.

***Pronotum*** (Fig. 33): Outline rectangular, transverse, a third wider than long. Lateral margin slightly and regularly convex, maximum width at middle. Lateral margin distinctly crenulated, with 13–15 distinct tubercles, tubercle anterior basal angle larger, with two notches at posterior angles. Base straight laterally, with declining flat keel at middle pointing posteriorly. Disc slightly flattened in lateral view, with two distinctly raised paramedian carinae parallel to median line and diverging posteriorly, with median line long, broad anteriorly and narrow posteriorly, with two additional shorter carinae bilaterally at base, joining with the paramedian carinae and forming tooth-like tubercle at base pointing posteriorly, without anterior extension, without inner lateral carina, with very small and indistinct outer lateral carinae. All carinae sub-crenulate. Lateral margin broadly wing-like bent up, with six large and deep transverse pits, the basal ones partly separated into two smaller pits. Space between carinae and pits smooth.

***Elytron*** (Fig. 49): Flattened in anterior half (lateral view), convex in frontal view. Sub-elongate, slightly convex laterally but not diverging, maximum width at middle, slightly narrowed posterior pseudohumerus. Pseudohumerus rectangular, without distinctly projecting tooth. Apex rounded, with small but acute tooth at suture. Disc with interneur six sub-crenulate, interneur one slightly carinate in basal quarter, interneur three slightly carinate in whole length, interneur two running up to apex as slightly convex line, conspicuously raised, reaching apex; interneur four running in parallel to interneur six, reaching base, shortened at apex. Interneur five and six with two rows of serial pits partly merging transversally.

***Hind wings:*** Fully developed.

***Lower surface:*** Antennal channel of pronotum with isodiametric reticulation. Pseudoepipleura with a short row of pits, lateral margin of elytron sub-crenulate. Metepisternum elongate, with broad longitudinal groove. Metasternum, abdominal sternites one and five with numerous irregularly situated larger and smaller pits, sternite two to three smooth, with band of small punctures at middle. Last two abdominal sternites laterally with isodiametric reticulation, sternite one with sub-longitudinal reticulation, two with irregular reticulation. Sternum four to six slightly sulcate. Sternum six with hollowed out circular impression at apex.

***Legs:*** Profemora with surface indistinctly reticulated. Protibia with robust, with moderately curved terminal spine, laterally with four teeth of decreasing size, the basal one with some distance from prebasal one, dorsally and ventrally with two carinae. First tarsomere distinctly elongated, as long as tarsomeres two to four together.

***External sexual dimorphism:*** Not observed.

***Male genitalia*** (Fig. 59): Median lobe stout but moderately slender in middle part, in dorsal view moderately arcuate, strongly bent in apical third, in lateral view convex, with few fine pili laterally in apical third, apical part bent regularly ventrally, thickened towards apex, in cross section spatula-like and hollowed out dorsally, oval at apex. Oroficium small. Endophallus with bundle of longer microtrichia near oroficium, with additional longitudinal group of small microtrichia basally. Dorsal paramere relatively short, distinctly bisinuate, with elongated apophyses; ventral one shaped like a longitudinal spatula, with fine seta at apex; both parameres slightly distorted.

***Female genitalia*** (Fig. 74): Coxostylus slender, abruptly broadened to base, distinctly curved, acute at apex, with indistinct carina dorsally in apical third, at end of basal third with one strong and seven slender nematiform setae laterally.

***Variation:*** The number of tubercles at the lateral margin of the pronotum varies from 13 to 15.

#### Distribution.

(Fig. 84). Known from several localities at the middle course of the river Niger and its confluents in Mali and the west of Niger.

#### Remark.

There is one additional female specimen from a different collection location with label data “BURKINA FASO BOROMO 11°45'06"N, 2°50'56"W / 9 VII 2005 LEG: P. MORETTO LIGHT TRAP” (CBP). This specimen shares some but not all characters with *S. nigeriensis* Alluaud. More material and/or a male specimen from that locality are needed for a proper identification.

### Salcedia putzeysi

Taxon classificationAnimaliaColeopteraCarabidae

(Oberthür, 1883)

E968F7C0-41BC-5F2A-81ED-DFC752C2DDB1

Figs 16 , 34 , 60 , 75 , 84

Holoprizus putzeysi Oberthür, 1883: XL; Csiki 1927: 553.
Salcedia putzeysi (Oberthür, 1883): Reichardt 1975: 103; Dostal 1993: 121; Lorenz 2005: 155.

#### Type material.

**Holotype**: ♂, with labels and data: beige, black framed and printed “Addah.W.Afrika Goldküste.” / white, handwritten “Holoprizus Putzeysi Oberth.” / red, black printed “TYPE” (MNHN).

#### Remark.

The holotype was in two pieces but glued. The aedeagus was dissected and stored in a microvial at the same pin as the specimen. The specimen is complete. The type label was hidden under the determination label.

#### Additional material.

4 ♂, 1 ♀, 2 specs., with labels and data: blue, printed, black framed “MUSEUM PARIS DAHOMEY ENV. DE PORTO-NOVO WATERLOT 1912” (MNHN, CBB), 1 spec. with label: beige, handwritten “*Salcedia schoutedeni* All.” (MNHN); 1 ♂, 1 ♀, with labels and data: white black printed “GHANA: Ashanti region Kumasi, Nhisau 330 m, N 6 43 – W1 36 Dr. S. ENDRODY-YOUNGA” / “Nr.284 black light 27.X.1967” (CBP).

#### Diagnosis.

A large sized species, with sub-elongate outline of the elytra with maximum width at middle and the pronotum with a short outer lateral carina near base. The pseudohumerus is rectangular and with an indistinct tooth. The antennomeres are elongate. Distinguished most clearly from the most similar species, *S. schoutedeni* and *S. nigeriensis*, by the head with an obtuse angle laterally at the base, the lateral outline of the elytra which are straight at middle, the less convex lateral margin of the pronotum, and by the different aedeagus. It differs most from *S. matsumotoi* sp. nov. by the different characters of the ratios of the elytra and the pronotum and the less numerous tubercles at the lateral margin.

#### Redescription.

Measurements in Table 1.

***Colour and surface:*** Griseous, shiny; top of carinae on head, pronotum and elytra as well as margins of pronotum and elytra opaque, covered with pale grey pili; legs and mandibles fuscous, antennae and palpi leoninous.

***Head:*** Three-quarters of pronotum width. Outline campanulate. Clypeus wide, slightly convex anteriorly, fused with clypeal wings, separated from supra-antennal plates by obtuse notches, with raised transverse field at middle, separated from frons by broad transverse furrow, frons with two raised paramedian carinae, joining anteriorly into a V-like keel, keel slightly tooth-like overhanging anteriorly, with two small glossy teeth bilaterally anterior to central keel, with a short distinctly diverging carina at each side paralaterally at base. Frons separated from supraorbital plates by flattened broad furrows; with conspicuously deep, broad pit at level of front of eyes; supra-antennal and supraorbital plates margined, margin of supraorbital plate distinctly raised, carina-like, supra-antennal plates slightly vaulted. Basal border with broad emargination at middle, obtuse angled laterally (angle 120–123°). Eyes large, convex, genae slightly convex, parts of both of them clearly visible from above, with slightly trapezoid shape in lateral view. Antenna with segments five to ten elongate (L/W 1.25), densely pubescent, segments two to four scarcely pubescent, scapus with sub-elongate reticulation. Labrum convex anteriorly. Mandible moderately short, wide, slightly arcuate at apex. Mentum small, convex anteriorly, without tooth, epilobes wide, projecting and angled anteriorly, slightly margined medially, surface smooth, with isodiametric reticulation.

***Pronotum*** (Fig. 34): Outline rectangular, transverse, a third wider than long. Lateral margin slightly and regularly convex, maximum width at middle. Lateral margin distinctly crenulate, with ten to eleven distinct tubercles, tubercle anterior basal angle slightly more prominent, with two notches at posterior angles. Base straight laterally, with declining obtuse keel at middle pointing posteriorly. Disc flattened in lateral view, with two distinctly raised paramedian carinae parallel to median line and diverging posteriorly, without gap-like notches, with median line long, broadened anteriorly and narrowed posteriorly, with two additional and shortened carinae bilaterally at base, joining with the paramedian carinae and forming tooth-like tubercle at base pointing posteriorly, without anterior extension, without inner lateral carina, with very small and indistinct outer lateral carinae. All carinae sub-crenulate. Lateral margin broadly wing-like bent up, with six large and deep transverse pits, the basal ones partly separated into two smaller pits. Space between carinae and pits smooth.

***Elytron:*** Slightly depressed in anterior half (lateral view), convex in frontal view. Sub-elongate, convex laterally but with straight part at middle, not diverging, maximum width at middle. Pseudohumerus rectangular, without distinctly projecting tooth. Apex rounded, with small but acute tooth at suture, slightly retracted. Disc with interneur six sub-crenulate, interneur one indistinctly carinate in anterior three-thirds, interneur three slightly carinate in whole length, interneur two running up to apex as nearly straight line, conspicuously raised, not reaching apex; interneur four running in parallel to interneur six, reaching base, shortened at apex. Interneur five and six with two rows of serial pits towards apex.

***Hind wings:*** Fully developed.

***Lower surface:*** Antennal channel of pronotum with isodiametric reticulation. Pseudoepipleura with a row of pits, pits transverse towards apex, lateral margin of elytron smooth. Metepisternum elongate, with broad flat longitudinal groove. Metasternum, abdominal sternites one and five with numerous irregularly situated larger and smaller pits, sternite two to three smooth. Last abdominal sternites laterally with isodiametric reticulation, sternite one with longitudinal reticulation, two with isodiametric reticulation. Sternum four to six slightly sulcate. Sternum six with longitudinal flat pits laterally, with keel at apex.

***Legs:*** Profemora with surface indistinctly reticulated. Protibia with robust, moderately curved terminal spine, laterally with four teeth of decreasing size and with equal distance from each other, dorsally and ventrally with two carinae. First tarsomeres distinctly elongated, as long as tarsomeres two to four together.

***External sexual dimorphism:*** Not observed.

***Male genitalia*** (Fig. 60): Median lobe stout, in dorsal view distinctly arcuate, slightly cracked at middle and at beginning of apical spatula, in lateral view convex, with very few fine pili in apical half, apical part oblique, in cross section suboval at apex. Oroficium small. Endophallus with dense group of brush-like microtrichia. Dorsal paramere relatively long, bisinuate, with moderate apophyses; ventral one relatively long, shaped club-like; both parameres slightly distorted.

***Female genitalia*** (Fig. 75): Coxostylus slender, regularly broadened to base, less distinct curved, acute at apex, with eight large nematiform setae in basal half, one of them situated towards middle, one strong one at end of basal third.

***Variation:*** The lateral margin of the pronotum varies from nearly straight (Ghana, Kumasi) to slightly more convex in specimens from Porto-Novo. The two specimens from the south of Ghana are slightly larger.

#### Distribution

(Fig. 84). Known from a lagoon in Ivory Coast and from Benin as well as from the south of Ghana.

The specimens from Porto-Novo, Benin (formerly Dahomey) are quoted in Alluaud (1935) under “localités nouvelles” as *S. schoutedeni*.

### Salcedia matsumotoi sp. nov.

Taxon classificationAnimaliaColeopteraCarabidae

820CDC15-FC9E-51F0-8DE3-27BAC99E2C98

http://zoobank.org/E45ECE64-EF94-4181-8C1A-8141E4137118

Figs 17 , 35 , 61 , 76 , 84

#### Type material.

***Holotype:*** ♂, with labels and data: white, printed “IVORY COAST 479 m Denguele Classified Forrest (sudanian forest) 09°30'0.6"N, 07°40'51.1"W 6–14.vi.2018” / “Actinic Light Trap Aristophanous, M., Miles, W., Moretto, P., Outtara. Y. leg. ANHRT: 2018.28, BMNH(E) 2018-153” / “NHMUK013685508 square barcode” (BMNH). ***Paratypes***: 3 ♀♀, same data as holotype but with third label “NHMUK013685506 square barcode”, and 013685505, 013685506, 013685507 (BMNH, CBB); 1 ♂, 1 ♀, 7 specs. same data as holotype but with third label “NHMUK013650247 square barcode”, 013650248 – 013650252, 013680129 and -38, 014055537 (BMNH, CBB); 1 ♀, with labels and data: white, printed “IVORY COAST 417 m Gbando Village (sudanian forest with gallery forest) 09°34'17.1"N, 06°41'1.1"W 15–22.vi.2018” / “MV Light Trap. Aristophanous, M., Outtara, Y. leg. ANHRT: 2018.28, BMNH(E) 2018-153” / “NHMUK013650253 square barcode” (BMNH).

#### Remark.

In one of the paratypes the left front tibia is missing, in another one three tarsomeres of the right intermediate and hind leg are missing.

#### Diagnosis.

A large sized species, with elongate outline of the elytra with maximum width at middle and the pronotum with nearly complete lateral carinae. The pseudohumerus is obtuse-angular and with a tooth. The antennomeres are elongate. Distinguished most clearly from the most similar species *S. putzeysi* by the elongate elytra and measurement ratios, the angle of the pseudohumerus, the pronotum with nearly complete lateral carinae, the female coxostylus with different setae pattern, and the different aedeagus.

#### Description.

Measurements in Table 1.

***Colour and surface:*** Fuscous, shiny; top of carinae on head, pronotum and elytra as well as margins of pronotum and elytra opaque, covered with pale grey pili; pronotum laterally translucent-hinnuleous, legs and mandibles fuscous, antennae and palpi hinnuleous.

***Head:*** Three-quarters of pronotum width. Outline campanulate. Clypeus wide, straight anteriorly, fused with clypeal wings, separated from supra-antennal plates by obtuse notches, with raised transverse field at middle, separated from frons by broad flat transverse furrow. Frons with two raised paramedian carinae, joining anteriorly into a V-like keel, keel tubercle-like increasing anteriorly, with two small glossy teeth bilaterally anterior to central keel, with a short parallel running carina at each side paralaterally at base; frons separated from supraorbital plates by flattened broad furrows; with conspicuously deep and broad pit at front-eye level; supra-antennal and supraorbital plates margined, margin of supraorbital plate distinctly raised, carina-like, with indistinct tubercle anterior hind angle; supra-antennal plates vaulted. Basal border with broad emargination at middle, angled laterally (angle around 122°). Eyes large, convex, genae flattened, parts of eyes visible from above, with indistinct triangle shape in lateral view. Antenna with segments five to ten elongate (L/W 1.24), densely pubescent, segments two to four scarcely pubescent, scapus with sub-elongate reticulation. Labrum like a flat triangle anteriorly. Mandible moderately short, wide, slightly arcuate at apex. Mentum small, ovoid, without tooth, with isodiametric reticulation; epilobes wide, projecting and angled anteriorly, margined anteriorly and slightly medially, surface covered with small pits.

***Pronotum*** (Fig. 35): Outline rectangular, transverse, somewhat smaller than a quarter wider than long. Lateral margin slightly and regularly convex, maximum width at middle. Lateral margin distinctly crenulated, with 13 tubercles, tubercle anterior basal angle slightly more prominent, with two notches at posterior angles. Base straight laterally, with declining flat keel at middle pointing posteriorly. Disc convex in lateral view, with two distinctly raised paramedian carinae parallel to median line and distinctly diverging posteriorly, with slight transverse notches, with median line long, broad anteriorly and narrow posteriorly, with two additional carinae bilaterally at base, joining with the paramedian carinae and forming tooth-like tubercle at base pointing posteriorly, with distinct anterior extension, with inner lateral carina of irregular structure, with outer lateral carinae becoming indistinct anteriorly. All carinae sub-crenulate. Lateral margin broadly wing-like bent up, with six large and deep transverse pits, the basal ones partly separated into two smaller pits. Space between carinae and pits smooth.

***Elytron:*** Indistinctly depressed in anterior third (lateral view), convex in frontal view. Elongate, slightly and regularly convex laterally, maximum width at middle. Pseudohumerus obtuse-angular (angle 114°), with projecting tooth. Apex rounded, with small but acute tooth at suture. Disc with interneur six sub-crenulate, interneur two running up to apex as indistinctly convex line, distinctly raised, reaching apex; interneur four running at middle in parallel to interneur six, nearly reaching base, shortened at apex. Interneur five and six with two rows of serial pits distinctly merging transversally.

***Hind wings:*** Fully developed.

***Lower surface:*** Antennal channel of pronotum with isodiametric reticulation. Pseudoepipleura with a row of indistinct pits, transverse apically, lateral margin of elytron indistinctly sub-crenulate. Metepisternum elongate, with moderately narrow longitudinal groove. Metasternum, abdominal sternites with numerous irregularly situated larger pits, sternite one and two with longitudinal reticulation. Last abdominal sternite laterally with irregular reticulation. Sternum four to six slightly sulcate. Last sternite with longitudinal flat keel laterally and at middle, apex somewhat hollowed out and with isodiametric reticulation.

***Legs:*** Profemora with surface indistinctly reticulated. Protibia with robust, slightly curved terminal spine, laterally with five teeth of decreasing size, the basal one with some distance from the others, dorsally and ventrally with two carinae. First tarsomeres distinctly elongated, as long as tarsomeres two to four together.

***External sexual dimorphism:*** Not observed.

***Male genitalia*** (Fig. 61): Median lobe long, slender, in dorsal view more evidently curved at end of basal third and in apical quarter, in lateral view nearly straight, with few scattered fine pili, apical part elongated, somewhat distorted, in cross section spoon-like dorsally. Oroficium small. Endophallus with three groups of microtrichia, two near oroficium and one basally. Dorsal paramere of moderate size, slightly bisinuate, with small elongated apophyses; ventral one shaped like a longitudinal spatula, both parameres slightly distorted, both of them hyaline at apex.

***Female genitalia*** (Fig. 76): Coxostylus slender, gently broadened to base, distinctly curved, acute at apex, apex somewhat rounded, with seven setae in basal half, one of them larger; SSO near apex with one microtrichium.

***Variation:*** On the pronotum the notches on the paramedian carinae show variability in the distinctness. The outer one of the three lateral carinae is becoming indistinct anteriorly to a different degree.

#### Etymology.

The species is dedicated to Keita Matsumoto (BMNH) who supported especially this work by intensive searching for *Salcedia* specimens among the huge West African material in the BMNH.

#### Distribution

(Fig. 84). Known from Denguele in the north-west of the Ivory Coast.

### Salcedia procera sp. nov.

Taxon classificationAnimaliaColeopteraCarabidae

F70D1751-B5C3-552E-8288-1857E3DBDA7B

http://zoobank.org/AD23EE1E-DDCE-44BB-B08F-65A80A794867

Figs 18 , 36 , 62 , 77 , 84

#### Type material.

***Holotype:*** ♂, with labels and data: white, printed and handwritten in black ink “Angola : Lac Calundo 105 km E. Vila Luso 4492.8 XII-1954 E. Luna de Carvalho” / green, black framed “Récolté sous des pierres” / white, handwritten in pencil “ANG:4592-8” (MRACT). ***Paratypes***: 1 spec., same data as holotype; 1 ♂, 1 ♀, same data as holotype but “4724-2”; 1 ♀, 3 specs., same data as holotype but with numbers 4510-18, 4538-11, 4643-5, 4623-3 and additional label “ A la lumière” (MRACT, CBB).

#### Remark.

In some of the paratypes, parts of the tarsomeres and antennae are missing.

#### Diagnosis.

A medium sized species, with super-elongate outline of the elytra with maximum width anterior and posterior middle and the pronotum with three additional complete carinae. The pseudohumerus is obtuse angular and dentate. The antennomeres are sub-moniliform. Distinguished most clearly from the similar species *S. africana* by the smaller size, the sub-moniliform antennomeres and the front tibia with four lateral teeth. The second similar species, *S. elongata* Alluaud is much larger, its antennomeres are moniliform, the elytra are oblong-elongate and the anterior angles of the pronotum are acutely produced anteriorly. Moreover, it is the only species of the genus with slightly concave outline of the elytra, so that the maximum width is anterior and posterior to the middle.

#### Description.

Measurements in Table 1.

***Colour and surface:*** Piceous, areas between carinae and pits shiny; legs, and antennae fuscous, palpi leoninous.

***Head:*** Three-quarters of pronotum width. Outline pentagonal shaped. Clypeus wide, straight anteriorly, fused with clypeal wings, separated from supra-antennal plates by distinct notches, with convex transverse field at middle, separated from frons by deep transverse furrow. Frons with two raised paramedian carinae, joining anteriorly into a central erected tubercle, with two minute acute glossy teeth bilaterally anterior to the central tubercle, with two parallel carinae paralaterally near base; frons and clypeus separated from supra-antennal and supraorbital plates by deep broad furrows, each furrow with deep slightly longitudinal pit between supra-antennal plate and clypeus; supra-antennal and supraorbital plates distinctly margined, margin raised, carina-like, nearly smooth, supra-antennal plates slightly vaulted. Base emarginated at middle, nearly right-angled laterally (angle 105°–107°). Eyes large, convex, with transverse shape in lateral view, with small part visible in dorsal view; genae slightly convex. Antenna with segments five to ten sub-moniliform (L/W 0.95–1.02), densely pubescent, segments two to four scarcely pubescent, scapus and pedicellus with longitudinal reticulation. Labrum convex anteriorly. Mandible moderately short, wide, slightly arcuate at apex. Apical segment of maxillary palpomere moderately long. Mentum small, elongated, epilobes elongated, projecting and distinctly angled anteriorly, margined medially, surface covered with flattened irregular pits.

***Pronotum*** (Fig. 36): Outline rectangular, transverse, a fifth wider than long. Lateral margin nearly straight at middle, slightly converging anteriorly, maximum width behind middle, slightly converging at anterior and posterior angles. Lateral margin indistinctly crenulated, with nine to ten small tubercles, tubercles sometimes doubled (100× magnification); with anterior angles right-angled, with distinct emargination at posterior angles. Base straight laterally, with distinct keel at middle. Disc with two raised paramedian carinae parallel to median line and diverging posteriorly, with two slight notches, with median line narrow and moderately short, ending in longitudinal pits anteriorly and posteriorly, with four additional shorter carinae bilaterally, the paralateral ones joining with the paramedian carinae at base and forming tooth-like tubercle pointing posteriorly, extended anteriorly up to anterior margin as less raised paralateral carina. With inner and outer lateral carinae well developed, the inner one connected with the extension of the paralateral carina, not connected with the outer lateral carina. All carinae sub-crenulate. Lateral margin and space between lateral margin and paralateral carina wing-like bent up, with six large transverse pits.

***Elytron:*** Flattened to slightly depressed in anterior half (lateral view), moderately convex in frontal view. Super-elongate, margin nearly parallel but slightly concave at middle, maximum width anterior and posterior middle. Pseudohumerus obtuse angular (angle 111°), dentate. Apex rounded, indistinctly denticulate at suture. Disc with interneur six sub-crenulate, interneur three slightly carinate in anterior third, interneur two running up to apex as parallel line, distinctly raised; interneur four running in parallel at middle, convex towards apex, almost reaching apex, nearly reaching base. Interval five and six with two rows of serial pits. Interneur five indistinctly visible due to transversally connected pits.

***Hind wings:*** Fully developed.

***Lower surface:*** Antennal channel of pronotum with isodiametric reticulation. Pseudoepipleura with one row of pits, interrupted at middle, lateral margin of elytron smooth. Metepisternum distinctly elongated, with broad longitudinal groove, groove isodiametrically reticulated. Last visible sternum with longitudinal carina at middle.

***Legs:*** Profemora dorsally with a small pit and irregular reticulation. Protibia of moderate length, with short curved and flattened terminal spine, laterally with four teeth of decreasing size, dorsally and ventrally with two carinae. Movable spur short, length a third of first tarsomere. Meso- and metafemora of moderate length, slender, slightly dilated basally. First tarsomere distinctly elongated, almost as long as tarsomere two to five together.

***External sexual dimorphism:*** Not observed.

***Male genitalia*** (Fig. 62): Median lobe conspicuously slender, elongated, in dorsal view regularly arcuate from base to apex, in lateral view slightly bisinuate, with single fine pili at beginning of apical third, apex spatulate, in cross section convex at apex, convexity directed dorsally. Oroficium of moderate size. Endophallus with two groups of microtrichia, a broader one with spine-like trichia, a small one at base of the first one at the opposite side. Dorsal paramere elongated, bisinuate, with elongated narrow apophyses; ventral one conspicuously small; both parameres slightly distorted.

***Female genitalia*** (Fig. 77): Coxostylus slender, obtusely curved, acute at apex, at end of basal third with one larger nematiform seta, five slender and one small nematiform setae laterally, SSO with one microtrichium.

***Variation:*** The number of small tubercles at the lateral margin of the pronotum varies from eight to ten. In some specimens the tubercles are doubled.

#### Etymology.

The species name refers to its very slender shape and is expressed as a Latinised adjective (procerus, a, um = slender).

#### Distribution.

Known from the type locality at the Lac Calundo in the west of Angola. The specimens were found under stones.

#### Remark.

There is one additional female specimen from a different collecting locality with label data “9.XI.2011 ANGOLA, Bié Province, 50 km NEE Kuito. Chissamba Mission P. Schüle leg.” (CBP). This specimen shares some characters with *S. procera* sp. nov. but it could not be determined with certainty and is therefore not included in the series of paratypes.

### Salcedia baroensis sp. nov.

Taxon classificationAnimaliaColeopteraCarabidae

D305BE20-5EE4-5307-92CB-742FBE62222F

http://zoobank.org/D1FFC9DF-18AD-4F55-83AA-C5200C2BB009

Figs 2 , 6b , 19 , 37 , 50 , 63 , 78 , 84

#### Type material.

***Holotype:*** ♂, with labels and data: white, black printed, with black cross stripe “Coll. Mus. Tervuren Ethiopie: Ilubabor prov Pokwo, Baro riv 25.VIII 72” / white, black printed “Coll. Mus. Tervuren Ethiopie R.O.S.Clarke” (MRACT). ***Paratypes***: 2 ♂♂, 4 ♀♀, 57 specs., same data as holotype (MRACT, CBB); 1 spec., white, black printed and handwritten with black ink “Illubabor Prov.: POKWO besides Bari Riv. VIII.72” / “Coll. Mus. Tervuren Ethiopie R.O.S. Clarke” (MRACT); 7 specs., white, black printed and handwritten with black ink “Illubabor Prov.: alt. 1800 m POKWO VIII.72” / “Coll. Mus. Tervuren Ethiopie R.O.S. Clarke” (MRACT); 1 ♂, 2 ♀♀, 16 specs., white, black printed “Illubabor Prov.: Gambela 16/19.X.1972” / “Coll. Mus. Tervuren Ethiopie R.O.S. Clarke” (MRACT, CBB); 2 specs., same data but “1 km W of Gambela 17XI.1972” (MRACT); 1 spec., same data but handwritten with black ink “21–23.V.1972” (MRACT).

#### Diagnosis.

A large sized species, with sub-elongate outline of the elytra with maximum width behind middle and the pronotum with a rudiment of the outer lateral carina at the posterior third. The pseudohumerus is rectangular and shows a laterally projecting tooth. The antennomeres are oblong-elongate. Distinguished most clearly from the similar species *S. nigeriensis* by the lateral margin of the pronotum with eleven tubercles and the oblique base. In addition, the elytra diverge slightly posteriorly. Moreover, it also differs from *S. utetea* sp. nov. in the elongate antennomeres, and shorter elytra.

#### Description.

Measurements in Table 1.

***Colour and surface:*** Piceous, shiny; top of carinae on head, pronotum and elytra as well as margins of pronotum and elytra opaque, covered with pale grey pili; legs and mandibles fuscous, antennae and palpi leoninous.

***Head:*** Four-fifths of the pronotum width. Outline semi-circular. Clypeus wide, distinctly convex anteriorly, fused with clypeal wings, separated from supra-antennal plates by obtuse notches, with raised pentagon-shaped field at middle, separated from frons by broad flattened shiny transverse furrow. Frons with two raised paramedian carinae, joining anteriorly V-like into a broad slightly elevated keel, with two small glossy teeth bilaterally anterior to central keel, with two short diverging carinae paralaterally at base; frons separated from supraorbital plates by flattened broad furrows; with conspicuously deep and broad pit at front-eye level; supra-antennal and supraorbital plates margined, margin of supraorbital plate distinctly raised, carina-like, supra-antennal plates slightly vaulted. Basal border with narrow emargination at middle, sub-rectangular laterally (angle around 107°). Eyes large, convex, genae slightly convex, both of them just visible from above, with longovoid shape and straight anterior and posterior margin in lateral view. Antenna with segments five to ten oblong-elongate (L/W 1.41), densely pubescent, segments two to four scarcely pubescent, scapus with longitudinal reticulation. Labrum completely covered by clypeus, convex anteriorly. Mandible moderately short, wide, regularly arcuate, more distinct at apex. Mentum small, sub-elongate, isodiametrically reticulated with obtuse tooth at middle, epilobes wide, projecting and angled anteriorly, margined laterally, surface with indistinct punctures, shiny.

***Pronotum*** (Fig. 37): Outline rectangular, transverse, a third wider than long. Lateral margin slightly and regularly convex, maximum width at middle. Lateral margin distinctly crenulated, with eleven distinct tubercles, tubercle anterior to basal angle larger, with two notches at posterior angles. Base oblique laterally, with declining flat ridge at middle. Disc flattened in lateral view, with two distinctly raised paramedian carinae slightly concavely depressed in lateral view, running parallel to median line and diverging posteriorly, with median line medium sized, ending in broad pits anteriorly and posteriorly; with two additional shorter carinae bilaterally at base, joining with the paramedian carinae and forming tooth-like tubercle at base pointing posteriorly, without anterior extension, without inner lateral carina, with very short and partly indistinct outer lateral carinae. All carinae sub-crenulate. Lateral margin broadly wing-like bent up, with six large and deep transverse pits, the basal ones somewhat flattened. Space between carinae and pits smooth at middle.

***Elytron*** (Fig. 50): Flattened and slightly depressed in anterior half (lateral view), convex in frontal view. Sub-elongate, slightly convex laterally and diverging posteriorly, maximum width slightly behind middle, indistinctly narrowed posterior pseudohumerus. Pseudohumerus rectangular, with laterally projecting tooth. Apex rounded, retracted, with small but acute tooth at suture. Disc with interneur six crenulated; interneur one with traces of carina basally, interneur three slightly carinate in whole length, interneur two running up to apex as slightly convex line, moderately raised, reaching apex; interneur four running in parallel to lateral carina, reaching base, distinctly shortened apically. Interneur five and six with two rows of serial pits, the latter ones partly merging transversally.

***Hind wings:*** Fully developed.

***Lower surface*** (Figs 2, 6b): Antennal channel of pronotum with isodiametric reticulation. Pseudoepipleura with two rows of pits, indistinctly hollowed out basally, lateral margin of elytron sub-crenulate. Metepisternum elongate, with broad longitudinal groove. Metasternum and abdominal sternite one with numerous irregularly situated larger and smaller pits, sternites two to four smooth, with band of small punctures at middle, sternite one and two with longitudinal reticulation, all other with isodiametric reticulation laterally. Sternum four to six slightly sulcate. Sternum six two irregular transverse rows of punctures, with obtuse transverse sinuate carina apically.

***Legs:*** Profemora with surface indistinctly reticulated. Protibia with short, robust, moderately curved terminal spine, laterally with four teeth of decreasing size, dorsally and ventrally with two carinae. First tarsomeres distinctly elongated, as long as tarsomeres two to four together.

***External sexual dimorphism:*** Not observed.

***Male genitalia*** (Fig. 63): Median lobe large, in dorsal view moderately arcuate from base up to apical fifth, in lateral view nearly straight, with fine pili laterally in middle third, apex thickened, convex, club-like, in cross section bilaterally with concavity at apex. Oroficium nearly half as long as median lobe. Endophallus with three groups of fine microtrichia, laterally with few larger ones in between. Dorsal paramere distinctly sinuate, with one minute seta at apex, with distinct apophyses, ventral one like a convex spatula, both parameres slightly distorted.

***Female genitalia*** (Fig. 78): Coxostylus slender, distinctly broadened to base, distinctly curved, acute at apex, with fine carina dorsally in apical third, at end of basal third with one larger and six slender nematiform setae laterally, SSO with one microtrichium.

***Variation:*** The slight carina on interneur three of the elytron shows variation in length, width and height, but is developed in all specimens. The indistinct thin carina at the base of interneur one varies much more, and is visible in approximately half of the specimens. At sternum six the number and position of punctures varies. In any case they form two irregular transverse bands of punctures. However, no differences between sexes were observed.

#### Etymology.

The name refers to the River Baro in the west of Ethiopia where the species has been found.

#### Distribution

(Fig. 84). Known from several localities at the River Baro, Illubabor province, in Ethiopia.

### Salcedia utetea sp. nov.

Taxon classificationAnimaliaColeopteraCarabidae

0AAB6D66-BA06-5AF0-92A2-4DF24EDB1041

http://zoobank.org/33502FE5-EE6E-4405-86AD-BC8FE93E1A6D

**Figures 20–25.**
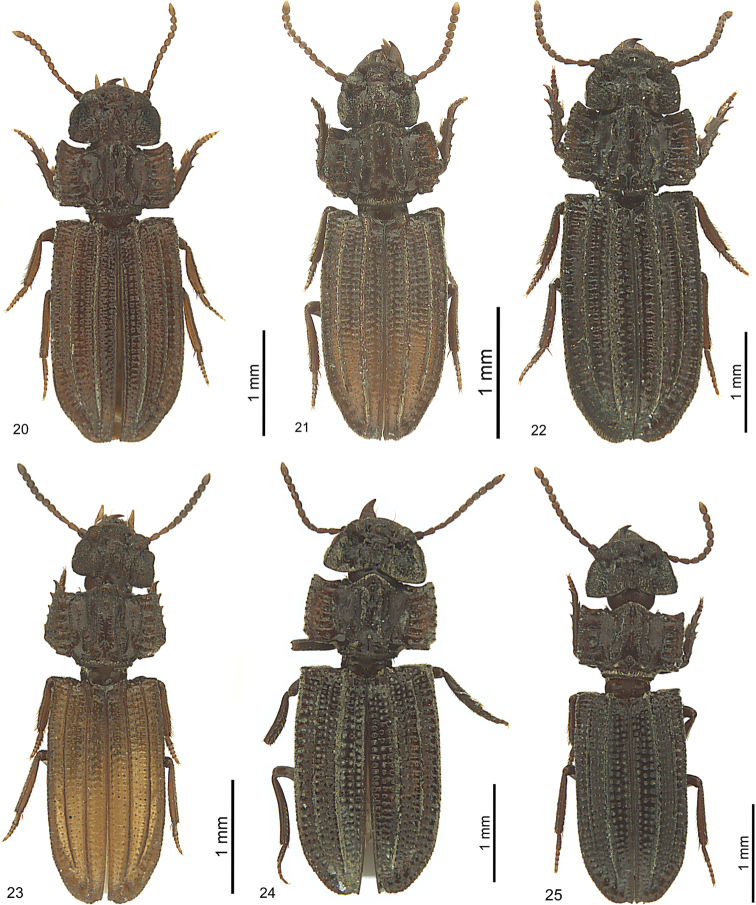
*Salcedia* species, habitus, dorsal view, holotype **20***S. utetea* sp. nov. **21***S. lukulua* sp. nov. **22***S. robusta* sp. nov. **23***S. tuberculata* sp. nov. **24***S. miranda* (Andrewes) **25***S. parallela* Baehr.

Figs 20 , 38 , 64 , 79 , 84

#### Type material.

***Holotype:*** ♂, with labels and data: white, black printed „TANZANIA or.mer. 8°03,2'S, 38°52,7'E; 70m 15 km N Utete, pr.Pwani leg. F. Kantner 16.i.2007” (SMNS). ***Paratypes***: 1 ♂, 1 ♀, 12 specs., same data as holotype (SMNS, CBB); 1 ♂, 3 specs., same data as holotype (CBP); 1 spec., “Tanzania prov. Pwani, 15 km E Utete, 75 m, 16.1.2007, leg. Halada / Salcedia sp. det. Schuh 2009 / Salcedia spec. P. Schüle det. 2012” (CBP); 1 ♂, with labels and data: beige, black printed and handwritten with black ink “Narobi b. Tanga D. O. Afr. V 1915 leg. Methner” / “COM. INST. ENT. COLL. NO. 13458” (MFNB); 1 ♂, same data as holotype but first label handwritten “Ksagara” (MFNB).

#### Diagnosis.

A medium sized species, with sub-elongate outline of the elytra with maximum width at middle and the pronotum with complete inner and outer lateral carinae. The pseudohumerus is sub-rectangular with a laterally projecting tooth. The antennomeres are elongate. Distinguished most clearly from the similar species *S. nigeriensis* by the lateral margin of the pronotum with ten tubercles and the complete set of carinae on the disc. In addition and in contrast to *S. nigeriensis*, the lateral margin of the elytron (not visible in dorsal view) is smooth.

#### Description.

Measurements in Table 1.

***Colour and surface:*** Piceous, shiny; top of carinae on head, pronotum and elytra as well as margins of pronotum and elytra opaque, covered with pale grey pili; legs, mandibles and antennae fuscous, palpi leoninous.

***Head:*** Two-thirds of width of pronotum. Outline campanulate. Clypeus wide, distinctly convex anteriorly, fused with clypeal wings, not separated from supra-antennal plates, with raised sub-square field at middle, separated from frons by broad flattened shiny transverse furrow. Frons with two raised paramedian carinae, joining anteriorly into a V-like keel, with two small glossy teeth bilaterally anterior to keel, with two short parallel carinae paralaterally at base; frons separated from supraorbital plates by flattened broad area; with conspicuously deep and broad pit at front-eye level; supra-antennal and supraorbital plates margined, margin of supraorbital plate distinctly raised, carina-like, supra-antennal plates slightly vaulted. Base with moderately broad emargination at middle, laterally obtuse-angled (angle 103°-105°), with small but distinct tooth at angle, with small notch anterior tooth. Eyes large, convex, genae slightly convex, both of them just visible from above, with long ovoid shape and straight posterior margin in lateral view. Antenna with segments five to ten elongate (L/W 1.3), densely pubescent, segments two to four scarcely pubescent, scapus with longitudinal reticulation. Labrum convex anteriorly. Mandible of moderate size, regularly arcuate, more distinct at apex. Mentum small, pentagon-like shaped, isodiametrically reticulated, without tooth, epilobes wide, projecting and angled anteriorly, margined completely, surface with medium sized pits, shiny.

***Pronotum*** (Fig. 38): Outline rectangular, transverse, a third wider than long. Lateral margin regularly convex, maximum width at end of second third. Lateral margin distinctly crenulated, with 10(-12) distinct tubercles, with a notch at posterior angles. Base oblique laterally, with declining flat ridge at middle. Disc flattened at middle in lateral view, with two distinctly raised paramedian carinae running parallel to median line and diverging at base, with row of ten small tubercles arranged saw-like, with median line medium sized, ending in broad pits anteriorly and posteriorly; with two additional shorter carinae bilaterally at base, joining with the paramedian carinae and forming tooth-like tubercle at base pointing posteriorly, with long anterior extension, with short inner lateral carina, with indistinct outer lateral carinae. All carinae sub-crenulate. Lateral margin broadly wing-like bent up, with six large and deep transverse pits. Space between carinae and pits smooth at middle.

***Elytron:*** Flattened in anterior half (lateral view), convex in frontal view. Sub-elongate, slightly convex laterally, maximum width at middle. Pseudohumerus sub-rectangular (angle 96°), with laterally projecting tooth. Apex rounded, without tooth at suture. Disc with interneur six crenulated; interneur three slightly carinate in basal sixth, interneur two running up to apex as slightly convex line, conspicuously raised, reaching apex; interneur four running in parallel to interneur six, not reaching base, distinctly shortened apically. Interneur five and six with two rows of serial pits, the latter ones distinctly merging transversally.

***Hind wings:*** Fully developed.

***Lower surface:*** Antennal channel of pronotum with isodiametric reticulation. Pseudoepipleura with row of small pits, lateral margin of elytron smooth. Metepisternum elongate, with broad longitudinal groove reticulated isodiametrically. Metasternum with distinct pits partly joining; abdominal sternite one with longitudinal reticulation, sternite two with isodiametric reticulation, other sternites with densely arranged pits, with isodiametric reticulation laterally. Sternum four to six sulcate. Sternum five to six with isodiametric reticulation laterally, sternum six with longitudinal large pit apically.

***Legs:*** Profemora with surface reticulated. Protibia with short, moderately curved terminal spine, laterally with three larger and three smaller teeth, dorsally and ventrally with two carinae. First tarsomeres distinctly elongated, as long as tarsomere two to four together.

***External sexual dimorphism:*** Last visible sternum in females with lateral and central longitudinal carinae slightly more distinct developed as in males.

***Male genitalia*** (Fig. 64): Median lobe small, in dorsal view slightly arcuate in basal half, distinctly arcuate in apical half, in lateral view distinctly cracked at beginning of apical third, with fine pili laterally in apical half, apex spatulate, in cross section with concavity at apex. Oroficium nearly half as long as median lobe. Endophallus with group of conspicuously regularly arranged microtrichia near oroficium, laterally with a small group. Dorsal paramere elongated, gently sinuate apically, with hyaline apex, with short apophyses, ventral one relatively long, both parameres slightly distorted.

***Female genitalia*** (Fig. 79): Coxostylus of moderate size, distinctly broadened at base, distinctly curved, moderately acute at apex, with indistinct carina dorsally in apical third, at end of basal third with one large and seven slender nematiform setae laterally, SSO with one fine pilus.

***Variation:*** On the head, the lateral margin of the supra-antennal and supraorbital plates shows a different degree of the slight crenulation. In one of the paratypes the clypeal wings are separated from the supra-antennal plates by slight obtuse notches. The pronotum with the lateral margin shows an inter- and intra-specific variation of the number of tubercles (12 to 15). The protibia exhibits in some specimens one or two additional small teeth with a seta. In one specimen from “Narobi b. Tanga” the pronotum is slightly more constricted anteriorly.

#### Etymology.

The name refers to the location Utete in Tanzania where most of the type material was found.

#### Distribution

(Fig. 84). Known from the type locality in the north-east of Tanzania. The abbreviation on the label (“D. O. Afr.”) refers to the former notation Deutsch Ost Afrika (German East Africa).

**Figures 26–35.**
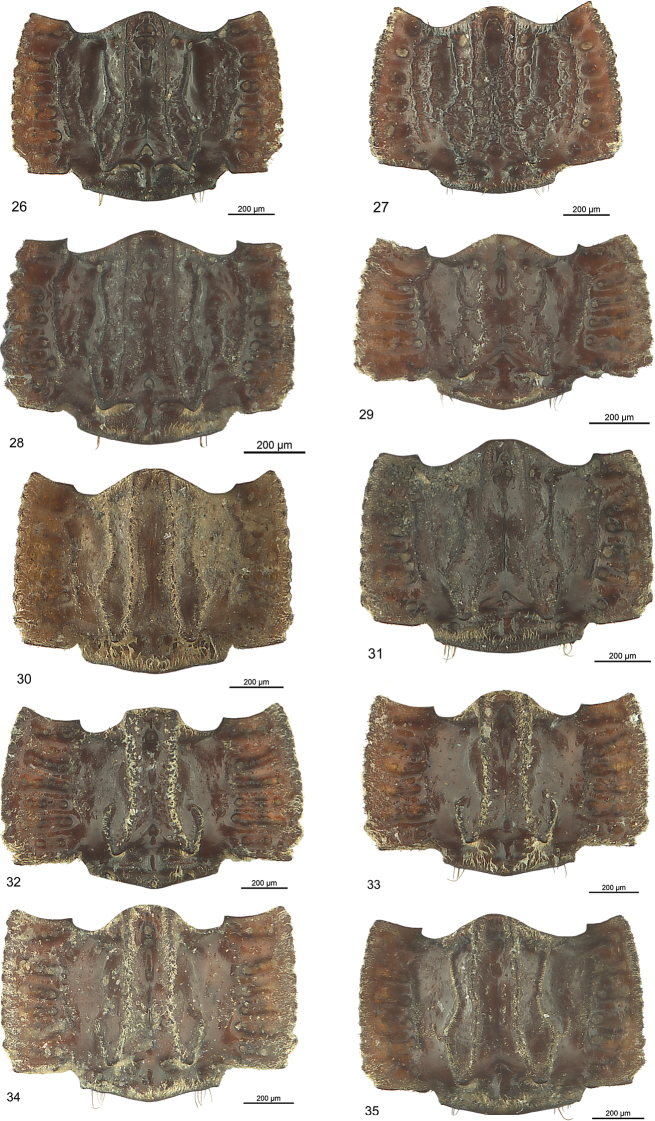
*Salcedia* species, pronotum, dorsal view **26***S. perrieri* Fairmaire **27***S. unifoveata* sp. nov. **28***S. faillei* sp. nov. **29***S. coquilhati* Alluaud **30***S. elongata* Alluaud **31***S. africana* (Britton) **32***S. schoutedeni* Alluaud **33***S. nigeriensis* Alluaud **34***S. putzeysi* (Oberthür) **35***S. matsumotoi* sp. nov.

### Salcedia lukulua sp. nov.

Taxon classificationAnimaliaColeopteraCarabidae

E32A2623-2DE7-59B0-8FA3-D4C69DFCF1B7

http://zoobank.org/3845BD70-983E-477F-92B0-B4ADB54C7117

Figs 4 , 5 , 21 , 39 , 80 , 84

#### Type material.

***Holotype:*** ♀, with labels and data: white, black printed, “I.R.S.A.C.-MUS. CONGO Katanga : Galerie forest , de la Lukulu, terr. Manono X-1958” / “Mission Z. Bacq N. Leleup” (MRACT). ***Paratype***: 1 ♀, same data as holotype (CBB).

#### Remark.

In the paratype two terminal antennomeres of the left antennae are missing.

#### Diagnosis.

A small sized species, with elongate oval outline of the elytra with maximum width at middle and the pronotum with three additional carinae with the inner lateral carina isolated. The pseudohumerus is obtuse angled and not dentate. The antennomeres are sub-elongate. Distinguished most clearly from the similar species *S. perrieri* by the distinctly convex outline of the elytra without tooth at the pseudohumerus, and the pronotum with the posterior lateral part of the base distinctly wing-like produced posteriorly with distinct notch between wing and central part of base, slightly emarginated posteriorly.

**Figures 36–43.**
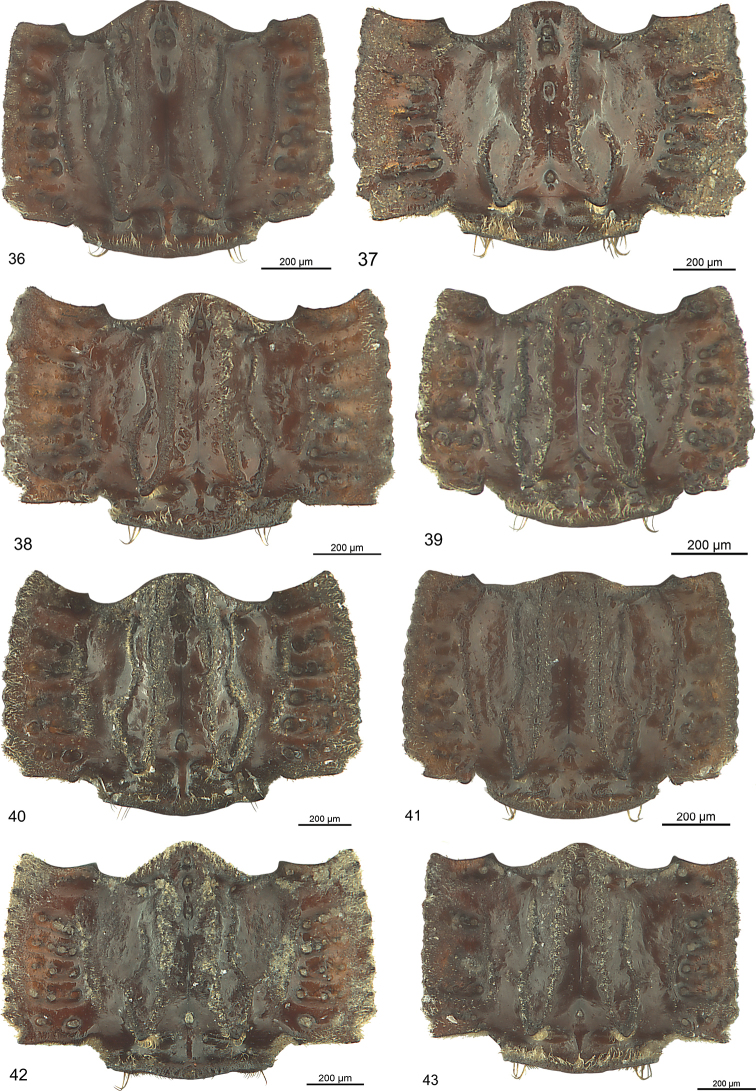
*Salcedia* species, pronotum, dorsal view **36***S. procera* sp. nov. **37***S. baroensis* sp. nov. **38***S. utetea* sp. nov. **39***S. lukulua* sp. nov. **40***S. robusta* sp. nov. **41***S. tuberculata* sp. nov. **42***S. miranda* (Andrewes) **43***S. parallela* Baehr.

#### Description.

Measurements in Table 1.

***Colour and surface:*** Piceous to slightly fuscous, shiny, top of carinae on head, pronotum and elytra opaque; mandibles, antennae, palpi and legs fuscous, antennae with scapus piceous.

***Head*** (Fig. 5): Three-quarters of pronotum width. Outline campanulate. Clypeus wide, straight anteriorly, separated from convex clypeal wings by indistinct obtuse notches, clypeal wings separated from supra-antennal plates by distinct notches, with raised oval field at middle, separated from frons by deep transverse furrow. Frons with two raised paramedian carinae, joining V-like anteriorly with minute tubercle, with two small obtuse teeth bilaterally anterior to central carinae, with two parallel carinae paralaterally near base; frons and clypeus separated from supra-antennal and supraorbital plates by deep broad furrows, each furrow with deep circular pit between supra-antennal plate and clypeus; supra-antennal and supraorbital plates distinctly margined, margin raised, carina-like, smooth, supra-antennal plates vaulted. Base distinctly emarginated bilaterally towards middle, obtuse angled laterally (angle 137°). Eyes convex, genae moderately convex, both of them partly visible from above, with triangle-like shape in lateral view, posteriorly emarginated. Antenna with segments five to ten sub-elongate (L/W 1.16), densely pubescent, segments two to four scarcely pubescent, scapus with longitudinal reticulation. Labrum obtuse angled anteriorly. Mandible moderately short, wide, slightly arcuate at apex. Mentum small, nearly circular, epilobes wide, projecting, nearly right-angled anteriorly, completely margined, surface with regular pits.

***Pronotum*** (Fig. 39): Outline rectangular, transverse, a third wider than long. Lateral margin convex, maximum width at end of second third, crenulated, more distinct posteriorly, with 12 (-13) tubercles, with distinct emargination at posterior angles. Base posteriorly wing-like produced, with distinct notch between wing and central part of base, slightly emarginated posteriorly, flat keel at middle broad, indistinct. Disc flattened (lateral view), with two raised paramedian carinae parallel to median line and slightly diverging posteriorly, with median line small, long, ending in pits anteriorly and posteriorly, with three additional shorter carinae bilaterally, the paralateral ones joining with the paramedian carinae at base and forming tooth-like tubercles pointing posteriorly, extended anteriorly as less raised paralateral carinae. With well-developed outer lateral carinae, with isolated short and less raised inner lateral carinae. All carinae sub-crenulate. Lateral margin and space between lateral margin and paralateral carina wing-like bent up, with six large transverse pits, each pit consisting of two deep circular and connected pits.

***Elytron:*** Flattened in anterior two thirds (lateral view), convex in frontal view. Elongate, margin long-convex, maximum width at middle. Pseudohumerus appearing nearly rectangular but is obtuse (angle 108°), not dentate. Apex rounded, with small acute tooth at suture. Disc with interneur six sub-crenulate, more distinct apically, interneur two running up to apex as slightly convex line, distinctly raised, conspicuously raised at base; interneur four running in parallel to interneur six, not reaching base, distinctly shortened at apex. Interneur five and six with two rows of serial pits, the latter ones distinctly merged transversally.

***Hind wings:*** Fully developed.

***Lower surface:*** Antennal channel of pronotum with isodiametric reticulation. Pseudoepipleura with two rows of pits, lateral pits larger; lateral margin of elytron smooth. Metasternum with numerous irregularly situated larger and smaller pits, last five abdominal sternites with small pits and irregular reticulation, four to six distinctly sulcate, last visible sternum with longitudinal carina at middle and three flat oval impressions apically.

***Legs:*** Profemora indistinctly reticulated. Protibia laterally with three larger teeth and with a smaller one basally, dorsally with one and ventrally with two carinae. Metafemora with transverse flat rugae at ventral surface.

***External sexual dimorphism:*** Not observed.

***Male genitalia:*** Unknown.

***Female genitalia*** (Fig. 80): Coxostylus broad at base, distinctly curved, moderately broad in apical third, at end of basal third with one shorter strong and five slender nematiform setae laterally, with one long slender nematiform seta ventrally, SSO with two microtrichia.

***Variation:*** The slight separation of the clypeus from the clypeal wings is more distinct in the paratype.

#### Etymology.

The name refers to the river Lukulu where the two specimens were found.

#### Distribution.

(Fig. 84). Known from the type locality at the River Lukulu (gallery forest) in Katanga, south-east of the Democratic Republic of the Congo.

**Figures 44–51.**
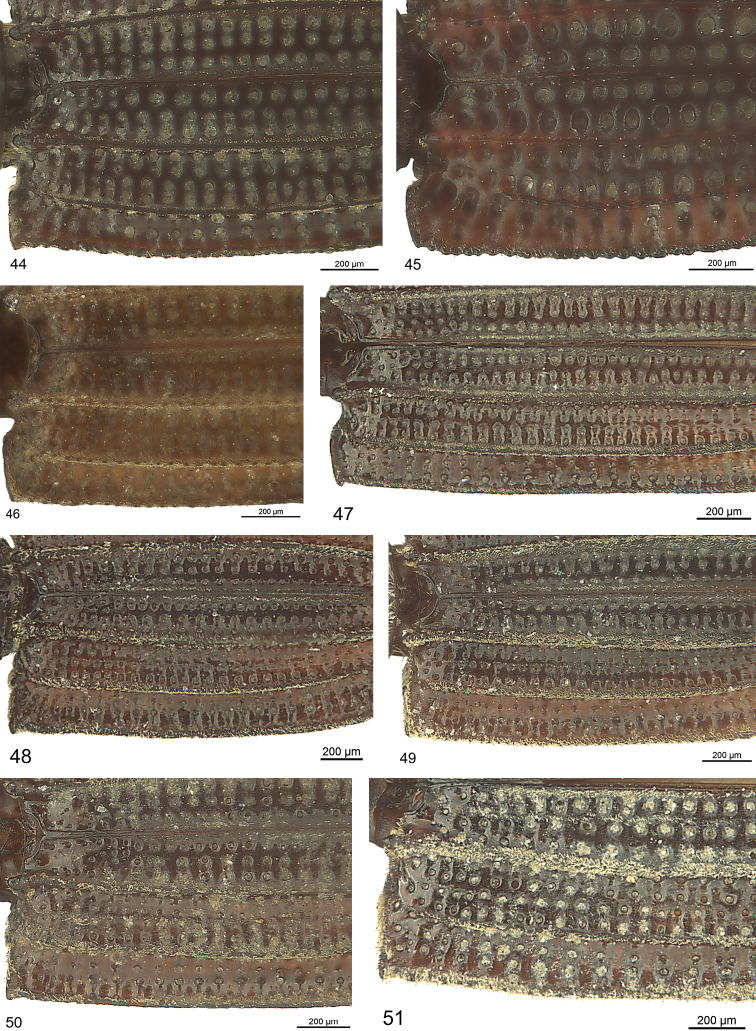
*Salcedia* species, left elytron, basal half, dorsal view **44***S. perrieri* Fairmaire **45***S. unifoveata* sp. nov. **46***S. elongata* Alluaud **47***S. africana* (Britton) **48***S. schoutedeni* Alluaud **49***S. nigeriensis* Alluaud **50***S. baroensis* sp. nov. **51***S. miranda* (Andrewes).

### Salcedia robusta sp. nov.

Taxon classificationAnimaliaColeopteraCarabidae

BF971697-AFEA-53F3-BB38-CFE85DBA14E3

http://zoobank.org/FF2CBE1C-3CD7-44DF-A479-26690C829D10

Figs 22 , 40 , 65 , 81 , 84

#### Type material.

***Holotype:*** ♂, with labels and data: white, printed, “MALAWI centr., Salima env., 5.–6.i.2002 J. Bezdĕk leg.” / “SALCEDIA elongata Alluaud, 1932 P. Bulirsch det. 2012” (SMNS). ***Paratypes***: 1 spec., same data as holotype (SMNS); 1 spec., “MALAWI centr., Nkhotakota env., 2.–3.i.2002, J. Bezdĕk leg.” / “Salcedia sp. det. SCHAWALLER 2003” / “SALCEDIA elongata Alluaud, 1932 P. Bulirsch det. 2012” (SMNS); 1 ♂, “MALAWI centr., Salima env., 5.–6.i.2002 J. Bezdĕk leg.” (CBP); 1 ♂ “MOZ.: CABO DELGADO Mareja (site 1), P. N. Quirimbas 100 m, 12°50'36"S, 40°10'53"E 23.xii.2012, degraded E Miombo woodlands; light traps; 2012-05 F. & S. Génier & M.Denja leg.“ (CBP); 1 ♂, 5 ♀♀, 183 specs., with labels and data: white, printed, “MOZ.: CABO DELGADO Mareja (site 1), P. N. Quirimbas 100 m, 12°50'36"S, 40°10'53"E 23.xii.2012, degraded E Miombo woodlands; light traps; 2012-05 F. & S. Génier & M.Denja leg.” (CFGC, MHNM, CBP, CBB); 1 ♂, 1 ♀, “MOZ.: CABO DELGADO Taratibu (site 7) P.N. Quirimbas 310 m., 12°48'25"S, 39°42'20"E 10.I.2013, eastern Miombo woodlands, light trap prairie edge F. & S. Génier & M. Denja, 2013-68” (CFGC);

#### Diagnosis.

A large sized species, with sub-elongate outline of the elytra with maximum width at middle and the pronotum with three additional carinae of which the inner lateral carina is distinctly shortened and the outer lateral carina slightly shortened anteriorly and posteriorly. The pseudohumerus is nearly rectangular with a tubercle at the edge not tooth-like projecting. The antennomeres are elongate. Distinguished most clearly from the similar species *S. schoutedeni* Alluaud. *S. nigeriensis* Alluaud, and *S. baroensis* sp. nov. by the 8–10 tubercles at the lateral margin of the pronotum. In addition, and unlike all other species, the two paramedian carinae of the pronotum are conspicuously raised with roughly sculptured tubercles which are distinctly interrupted by two deep gap-like notches.

**Figures 52–57.**
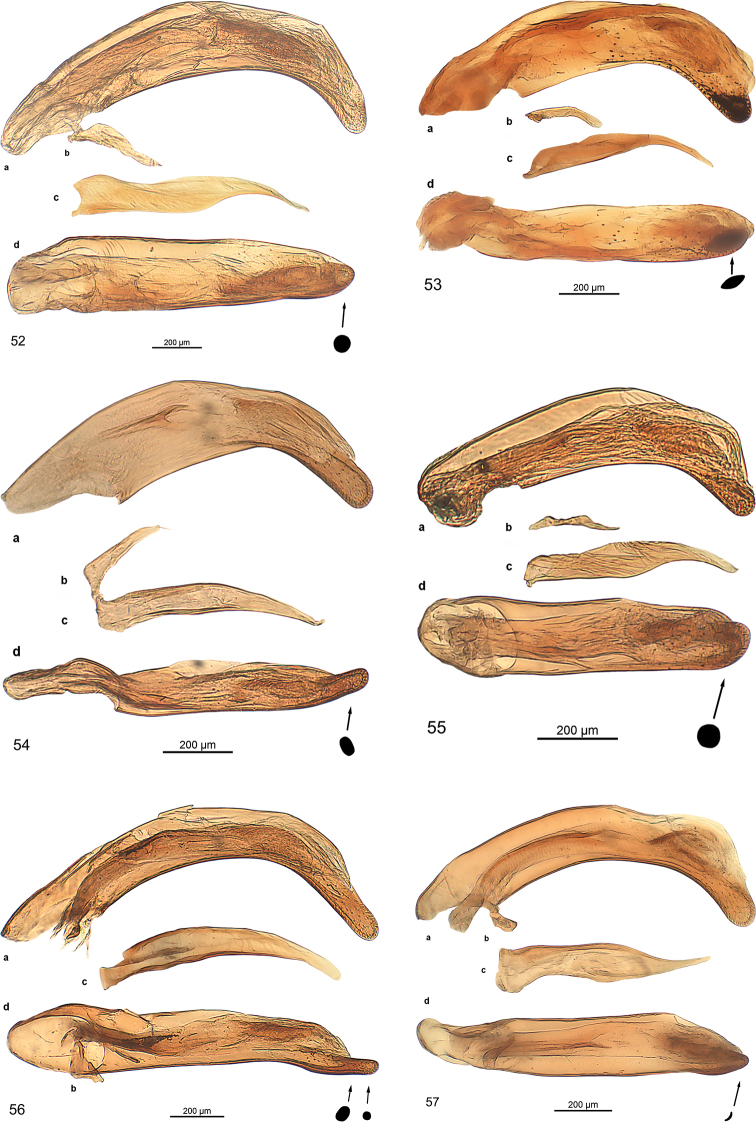
*Salcedia* species, male genitalia, dorsal view of aedeagus (**a**) and parameres (**b, c**), lateral view (**d**). Small sketches in black symbolising the apex in cross section **52***S. perrieri* Fairmaire **53***S. unifoveata* sp. nov. **54***S. faillei* sp. nov. **55***S. coquilhati* Alluaud **56***S. elongata* Alluaud **57***S. africana* (Britton).

#### Description.

Measurements in Table 1.

***Colour and surface:*** Anthracite grey to piceous, areas between carinae and pits shiny, carinae on both sides densely covered with band of grey pili; lateral fifth of pronotum fuscous; mandibles, legs and antennae piceous, palpi fuscous. Surface in general with less mud, but the layer is thicker.

***Head:*** Three-quarters of pronotum width. Outline shaped like a frustum of a pyramid. Clypeus wide, straight anteriorly, fused with clypeal wings, separated from supra-antennal plates by distinct notches, with transverse convex field at middle, separated from frons by moderately deep transverse furrow: Frons with two paramedian carinae tubercle-like raised, joining anteriorly, prolonged anteriorly into a central horn-like erected tubercle, with two small acute teeth bilaterally anterior to central tubercle, with two parallel sharp carinae paralaterally near base; frons and clypeus separated from supra-antennal and supraorbital plates by deep broad furrows, each furrow with deep transverse pit between supra-antennal plate and clypeus; supra-antennal and supraorbital plates distinctly margined, margin raised, carina-like, smooth, supra-antennal plates slightly vaulted, surface of supraorbital and supra-antennal plates reticulated, with some flat rugae. Basal border emarginated at middle, obtuse angled laterally (angle 115–117°). Eyes convex, with transverse triangular shape in lateral view, with small part just visible in dorsal view; genae slightly convex, with small but distinct notch anterior angle, separated from supraorbital plates by distinct notch. Antenna with segments five to ten elongate (L/W 1.3), densely pubescent, segments two to four scarcely pubescent, scapus and pedicellus with irregular reticulation. Labrum just visible from above, slightly pointed anteriorly. Mandible robust, wide, irregularly reticulated, slightly arcuate at apex. Epilobe of mentum moderately slender, flattened, projecting and distinctly angled anteriorly, not margined laterally, surface covered with flattened small pits; mentum indistinctly isodiametrically reticulated.

***Pronotum*** (Fig. 40): Outline rectangular, transverse, one third wider than long. Lateral margin distinctly convex, converging to anterior angles, maximum width slightly behind middle. Lateral margin distinctly crenulated, with eight to ten tubercles, with distinct emargination at posterior angles. Base straight laterally, posteriorly slightly produced, with small notch between wing and central part of base, with longitudinal keel at middle. Disc with two raised paramedian carinae parallel to median line, nearly parallel, diverging at posterior end, with conspicuously raised and roughly sculptured tubercles, distinctly interrupted by two to three deep gap-like notches, with median line moderately long, ending in pits anteriorly and posteriorly, with four additional shorter carinae bilaterally, the paralateral one joining with the paramedian carinae at base and forming tooth-like tubercle pointing posteriorly, extended anteriorly as distinctly raised paralateral carina. With minute rudiment of inner lateral carina, and anteriorly and posteriorly shortened outer lateral carina. With exception of paramedian carina, all others nearly smooth on top. Lateral margin and space between lateral margin and paralateral carina wing-like bent up, with six large transverse pits.

***Elytron:*** Flattened in anterior two-thirds (lateral view), regularly convex in frontal view. Sub-elongate, margin slightly convex, straight but indistinctly diverging in anterior fifth, maximum width at middle. Pseudohumerus slightly obtuse angular (angle 96°), tubercle at edge not tooth-like projecting. Apex retracted, denticulate. Disc with interneur six sub-crenulate, interneur three with indistinct traces of carina in anterior two thirds, interneur two running up to apex as indistinct convex line, moderately raised, not reaching apex; interneur four convex, running nearly in parallel to lateral carina, not reaching apex, shortened at base. Interneur five and six with two rows of serial pits partly merging transversally.

***Hind wings:*** Fully developed.

***Lower surface:*** Antennal channel of pronotum with irregular reticulation. Pseudoepipleura with two rows of pits, lateral margin of elytron smooth. Metepisternum distinctly elongated, with broad longitudinal groove. Last visible sternum with acute longitudinal carina at middle, hollowed out laterally.

***Legs:*** Profemora with dorsal surface indistinctly irregularly reticulated. Protibia with terminal spine curved at apex, laterally with four teeth of decreasing size, dorsally and ventrally with two carinae. Movable spur short, length a third of first tarsomere, curved slightly ventrally. Meso- and metafemora of moderate length, slender. First tarsomere distinctly elongated, almost as long as tarsomeres two to five together, front and intermediate legs with tarsomeres two to four broadened and somewhat flattened.

***External sexual dimorphism:*** Last visible sternum in males with surface more roughly sculptured.

***Male genitalia*** (Fig. 65): Median lobe relatively large, in dorsal view slightly to moderately arcuate from base up to apical fifth, in lateral view indistinctly bisinuate, with fine pili in apical quarter, apex spatulate, in cross section explanate at apex. Oroficium half as long as median lobe. Endophallus with conspicuous group of microtrichia, with finer and short trichia basally and apically, with long spines at middle. Dorsal paramere elongated, bisinuate, with elongated narrow apophyses; ventral one like an elongated triangle; both parameres slightly distorted.

***Female genitalia*** (Fig. 81): Coxostylus distinctly sclerotised, broad, distinctly curved, with distinct carina dorsally in apical third, apex somewhat rounded, at end of basal third with one strong and five slender nematiform setae laterally, SSO with one fine pilus.

***Variation:*** The lateral margin of the pronotum shows in the majority of the specimens eight tubercles but tend to then in some specimens. On the pronotum the inner lateral carina is small in general but varies in size. At the front tibia one of the lateral teeth is sometimes missing at one side. In the single male specimen from Malawi the front tibia shows an additional small tooth basally which adds up to five lateral teeth.

#### Etymology.

The species name refers to its size and robust appearance and is expressed as a Latinised adjective.

#### Distribution

(Fig. 84). The species was collected at different locations in Malawi and at two sites in the north-east of Mozambique.

**Figures 58–63.**
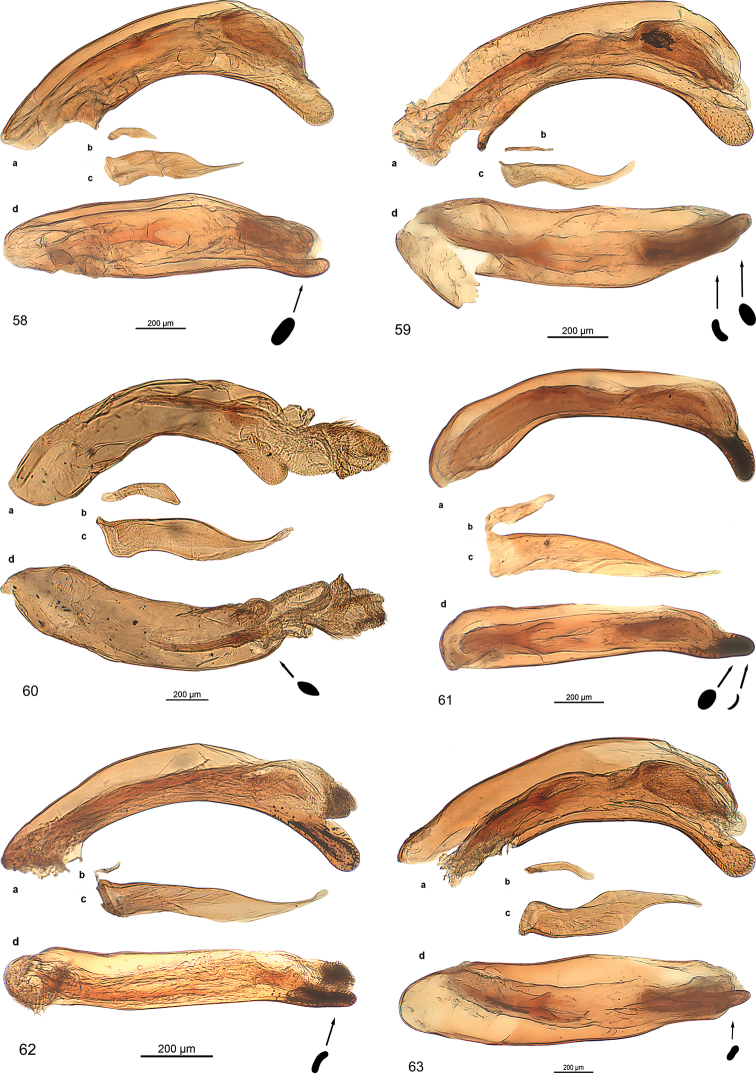
*Salcedia* species, male genitalia, dorsal view of aedeagus (**a**) and parameres (**b, c**), lateral view (**d**). Small sketches in black symbolising the apex in cross section **58***S. schoutedeni* Alluaud **59***S. nigeriensis* Alluaud **60***S. putzeysi* (Oberthür) **61***S. matsumotoi* sp. nov. **62***S. procera* sp. nov. **63***S. baroensis* sp. nov.

### Salcedia tuberculata sp. nov.

Taxon classificationAnimaliaColeopteraCarabidae

704FD768-39B5-5F92-ACDA-9F7A31B9BF51

http://zoobank.org/7367647f-c04e-4326-a9ab-8502fc1a4ae2

Figs 23 , 41 , 66 , 84

#### Type material.

**Holotype**: ♂, with labels and data: white, printed “S. Afr; KrugerNat. Pk PundaMariaNgotsodam 21.26S, 31.14E / 7.2.1994; E-Y: 2984 shorewashing leg. Endrödy-Younga” / “Salcedia africana (BRITT.) det.M.Baehr’03” / “SALCEDIA africana (Britton 1947) P. Bulirsch det. 2012” (SMNS).

#### Diagnosis.

A medium sized species, with oblong-elongate outline of the elytra with maximum width at middle and the pronotum with three additional complete carinae. The pseudohumerus is nearly rectangular and not dentate. The antennomeres are sub-elongate. Distinguished most clearly from the similar species *S. africana* by the pronotum with its base laterally with straight but obtuse lobe produced posteriorly and with a distinct peg-like tubercle. Distinguished at the first glance from all other species by the interneur four of the elytron which shows a distinct knob-like tubercle at base (in addition to the tubercle at base of the interneur two present in all species).

**Figures 64–67.**
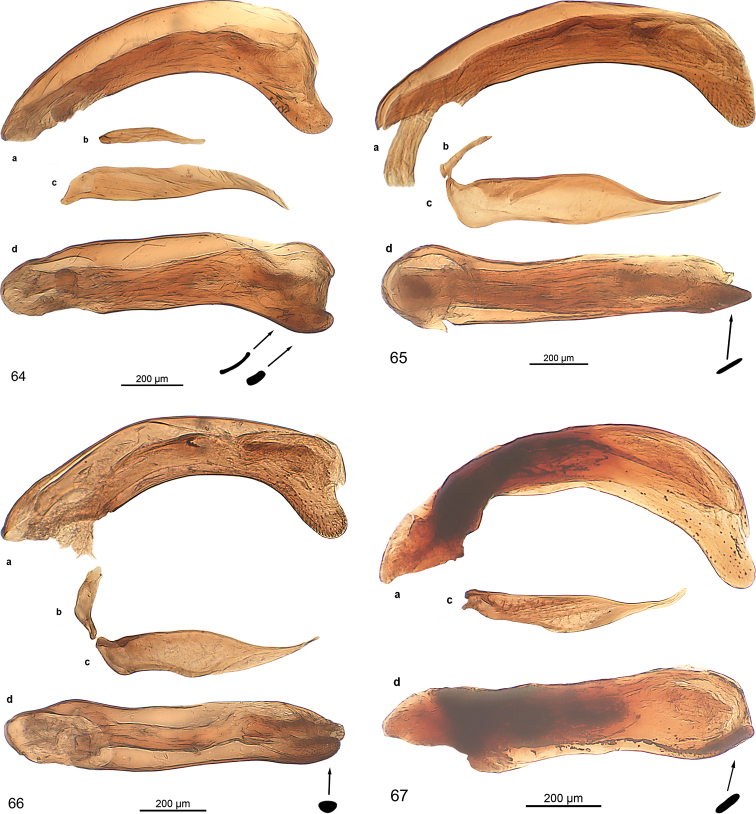
*Salcedia* species, male genitalia, dorsal view of aedeagus (**a**) and parameres (**b, c**), lateral view (**d**). Small sketches in black symbolising the apex in cross section **64***S. utetea* sp. nov. **65***S. robusta* sp. nov. **66***S. tuberculata* sp. nov. **67***S. miranda* (Andrewes).

#### Description.

Measurements in Table 1.

***Colour and surface:*** Fuscous to piceous, areas between carinae and pits shiny; lateral fifth of pronotum fuscous; mandibles piceous; legs, antennae and palpi fuscous; pronotum with posterior and lateral margin densely covered with beige-grey pili, more intensive around posterior angles.

***Head:*** Four-fifths of the pronotum width. Outline shaped like a frustum of a pyramid. Clypeus wide, indistinctly convex anteriorly, fused with clypeal wings, separated from supra-antennal plates by distinct obtuse notches, with square elevated field at middle, separated step-like from frons. Frons with two raised paramedian carinae, joining anteriorly, prolonged anteriorly into a central keel, with two small acute glossy teeth bilaterally anterior to central keel, with two parallel carinae paralaterally near base, converging anteriorly, broadened basally; frons and clypeus separated from supra-antennal and supraorbital plates by deep broad furrows, each furrow with deep circular pit between supra-antennal plate and clypeus; supra-antennal and supraorbital plates margined, margin raised, carina-like, at supraorbital plates excised concavely, supra-antennal plates slightly vaulted, surface of supraorbital and supra-antennal plates with numerous small flat irregular impressions. Basal border indistinctly emarginated at middle, distinctly and obtuse angled laterally (angle 118–120°). Eyes convex, oval shaped in lateral view, with small part just visible in dorsal view; genae slightly convex, with small notch anterior angle. Antenna with segments five to ten sub-elongate (L/W 1.17), densely pubescent, segments two to four scarcely pubescent, scapus and pedicellus with indistinct reticulation. Labrum clearly visible from above, nearly straight anteriorly. Mandible moderately short, wide, regularly arcuate at apex. Mentum pentagonal; epilobe of mentum moderately wide, projecting and distinctly angled anteriorly, completely margined, somewhat hollowed out, surface covered regularly with pits.

***Pronotum*** (Fig. 41): Outline rectangular, transverse, one fifth wider than long. Lateral margin convex, converging to anterior angles, maximum width at end of second third. Lateral margin distinctly crenulated, with thirteen tubercles, with distinct emargination at posterior angles, posterior angles acute. Base laterally with straight but obtuse lobe produced posteriorly, lobe with tubercle towards middle, with broad and deep emargination, with broad flat keel at middle. Disc, with two raised paramedian carinae parallel to median line, diverging posteriorly, finely tuberculate on top, with median line small, ending in pits anteriorly and posteriorly, with four additional shorter carinae bilaterally, the paralateral one joining with the paramedian carinae at base and forming tooth-like tubercle pointing posteriorly, extended anteriorly as less raised paralateral carina. With shorter not raised inner lateral carina and quite long more raised outer lateral carina. Carinae uneven on top. Lateral margin and space between lateral margin and paralateral carina wing-like bent up, with six large transverse pits.

***Elytron:*** Indistinctly depressed in anterior half (lateral view), moderately convex in frontal view. Oblong-elongate, margin at middle straight but indistinctly diverging, maximum width at middle. Pseudohumerus nearly rectangular, not dentate. Apex long-oval, retracted. Disc with interneur six indistinctly crenulated, interneur two running up to apex as indistinctly convex line, moderately raised; interneur four running slightly convex at middle, distinctly convex towards apex, not reaching apex, reaching base, with distinct knob-like tubercle at base. Interneur five and six with two rows of serial pits.

***Hind wings:*** Reduced by approximately 50%.

***Lower surface:*** Antennal channel of pronotum with isodiametric reticulation. Pseudoepipleura with two rows of pits, lateral margin of elytron smooth. Metepisternum moderately long, with flat longitudinal groove. Last visible sternum with obtuse longitudinal carina at middle, with rough pits laterally.

***Legs:*** Profemora with dorsal surface roughly reticulated. Protibia longer, with terminal spine curved regularly, laterally with four teeth of decreasing size, dorsally and ventrally with two carinae. Movable spur short, length a third of first tarsomere, slightly curved ventrally. Meso- and metafemora of moderate length, slender. First tarsomeres distinctly elongated, almost as long as tarsomeres two to five together.

***External sexual dimorphism:*** Not observed.

***Male genitalia*** (Fig. 66): Median lobe moderately short, in dorsal view cracked at end of basal third and beginning of apical third, in lateral view bisinuate, with fine scattered pili from basal third nearly up to apex, apex sub-spatulate, in cross section explanate dorsally, convex ventrally. Oroficium half as long as median lobe. Endophallus with elongated group of microtrichia conspicuously regularly arranged, with additional two small groups of finer and short trichia basally and apically. Dorsal paramere elongated, bisinuate, with elongated apophyses, broader at base; ventral one like spatulate, hyaline at apex; both parameres slightly distorted.

***Female genitalia:*** Unknown.

***Variation:*** Unknown.

#### Etymology.

The name refers to the distinct tubercle at base of the interneur four of the elytra as well as to the distinct tubercles at the base of the pronotum.

#### Distribution

(Fig. 84). Known from the type locality, in the Kruger National Park, Republic of South Africa.

**Figures 68–76.**
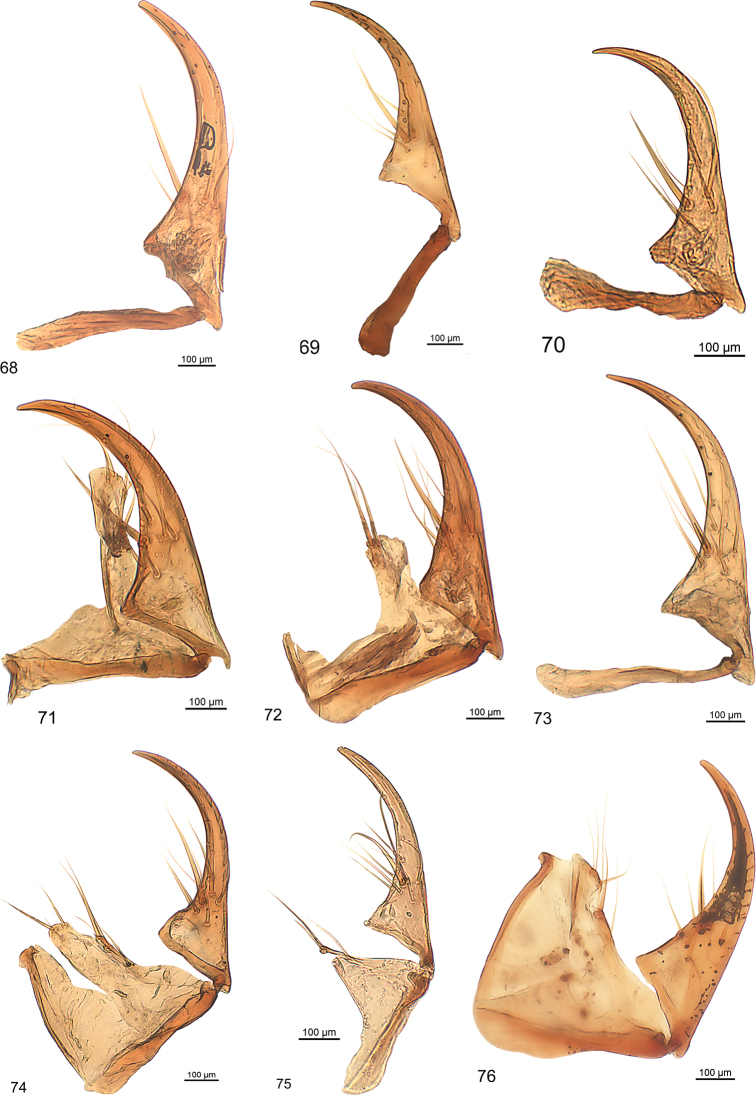
*Salcedia* species, female coxostyli (gonopod IX) and laterotergite IX (epipleutite IX) **68***S. perrieri* Fairmaire **69***S. unifoveata* sp. nov. **70***S. coquilhati* Alluaud **71***S. elongata* Alluaud **72***S. africana* (Britton) **73***S. schoutedeni* Alluaud **74***S. nigeriensis* Alluaud **75***S. putzeysi* (Oberthür) **76***S. matsumotoi* sp. nov.

### Salcedia miranda

Taxon classificationAnimaliaColeopteraCarabidae

(Andrewes, 1920)

41ECB288-7740-5AA8-BBF0-6E3A05795857

Figs 24 , 42 , 51 , 67 , 82

Zelma miranda Andrewes, 1920: 453: Csiki 1927: 546; Andrewes 1929: 418; Andrewes 1930: 358.
Salcedia miranda (Andrewes, 1920): Alluaud 1930: 22; Alluaud 1935: 19; Andrewes 1936: 64; Jeannel 1946: 233.
Zelma miranda Andrewes, 1920: Britton 1947: 127.
Salcedia miranda (Andrewes, 1920) Reichardt 1975: 103; Dostal 1993: 121; Baehr 1998: 2; Lorenz 2005: 155.

#### Type material.

**Holotype**: ♂, with labels and data: white, printed “Tharrawaddy Burma” / white, handwritten and printed “Zelma miranda Type Andr. H.E.Andrewes det.” / red, black printed “Type” / white, black printed “figured specimen”, backside black handwritten “F.B.I.” / white, printed “H.E.Andrewes Coll. B.M.1945-97.” (BMNH).

#### Additional material.

1 ♀, with labels and data: beige, printed, black framed “Palon (Pegu) L.Fea VIII.IX.87” / “Mus. Civ. Genova” / white, printed “H. E. Andrewes Coll. B. M. 1945-97.” (BMNH); 1 ♀, with labels and data: beige, printed, black framed “Palon (Pegu) L. Fea VIII.IX.87” / “MUSEUM PARIS COLL. A. GROUVELLE 1917” / white, handwritten “Versim.: Salcedia miranda Andr.” / beige, handwritten “nettoyage necessaire pour determ. precise. Alluaud 1934” (MNHN).

#### Remark.

In the holotype there is an additional mounting card on the pin where the following parts are glued: one front and one hind leg, complete mentum with right paragena and pair of labial palpi, right glossa with right maxillary palpus. An additional drop of glue indicates there might have been another piece of the body which is obviously lost. The following parts are missing from the specimen: left front leg with tibia onwards, left intermediate tarsomeres, right mandible.

#### Diagnosis.

A large sized species, with sub-elongate outline of the elytra with maximum width at middle and the pronotum with three additional carinae of which the inner lateral carina is distinctly shortened. The pseudohumerus is rectangular and with a non-projecting tooth. The antennomeres are oblong-elongate. Distinguished most clearly from the similar species *S. parallela* by the pronotum with the lateral margin convex and with seven to nine distinct tubercles. Moreover, in *S. miranda* the intervals five and six of the elytron each have two rows of pits.

**Figures 77–83.**
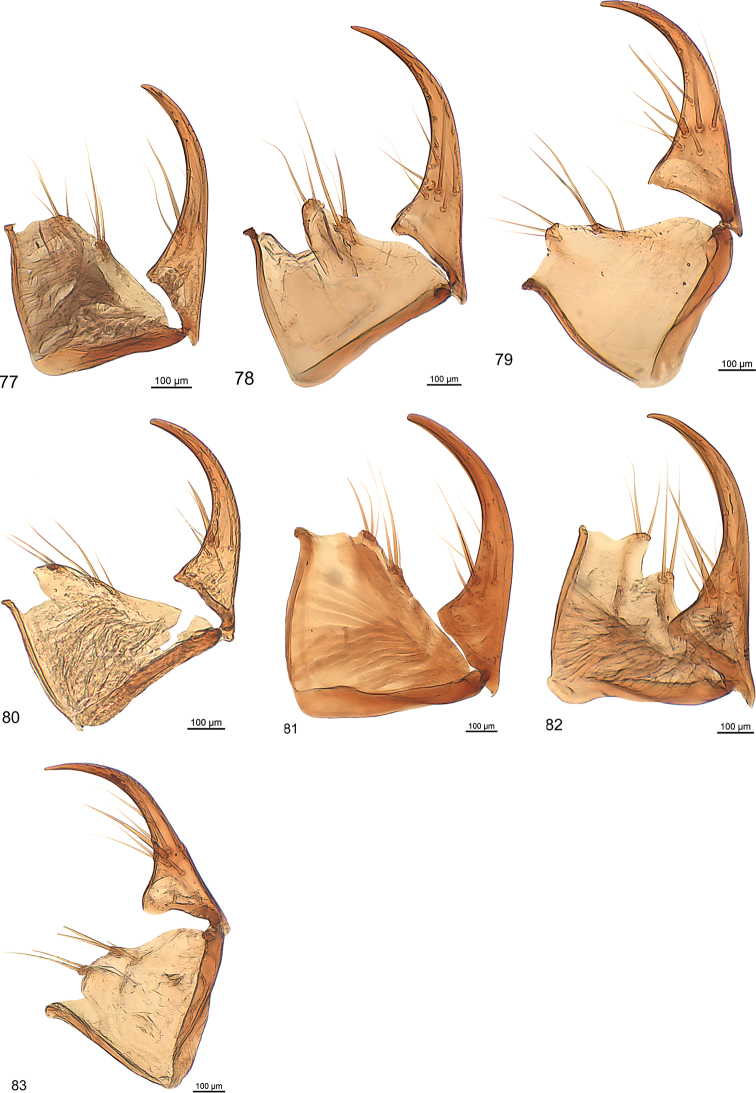
*Salcedia* species, female coxostyli (gonopod IX) and laterotergite IX (epipleutite IX). n. **77***S. procera* sp. nov. **78***S. baroensis* sp. nov. **79***S. utetea* sp. nov. **80***S. lukulua* sp. nov. **81***S. robusta* sp. nov. **82***S. miranda* (Andrewes) **83***S. parallela* Baehr.

#### Redescription.

Measurements in Table 1.

***Colour and surface:*** Piceous, shiny; top of supraorbital and supra-antennal plates, carinae on head, pronotum and elytra as well as margin of pronotum and elytra densely covered with beige-grey pili; legs fuscous, mandibles and first segments of antennae piceous, antennae brightened apically, palpi leoninous.

***Head:*** Four-fifths of the pronotum width. Outline semi-circular. Clypeus wide, slightly convex anteriorly, fused with clypeal wings, separated from supra-antennal plates by distinct notches and deep pit at each side, with raised semi-circular-shaped field at middle, separated from frons by deep transverse furrow. Frons with two raised paramedian carinae, carinae not joining anteriorly, with two small glossy teeth bilaterally to median carinae, with two shorter diverging carinae paralaterally at base; frons separated from supraorbital plates by deep broad furrows; with conspicuously broad pit at front-eye level; supra-antennal and supraorbital plates margined, margin raised, carina-like, supra-antennal plates vaulted. Base with angled emargination at middle (angle 100°), laterally right-angled (angle 90°). Eyes large, convex, genae higher as eyes, convex, both of them clearly visible from above, in lateral view shaped like a broad strip. Antenna with segments five to ten oblong-elongate (L/W 1.44), densely pubescent, segments two to four scarcely pubescent, scapus with longitudinal reticulation. Labrum slightly convex anteriorly. Mandible moderately short, wide, slightly arcuate at apex. Mentum small, with slight rounded tooth at middle, epilobes wide, projecting, acutely angled anteriorly, margined apically, surface uneven, with irregular reticulation.

***Pronotum*** (Fig. 42): Outline rectangular, transverse, a third wider than long. Lateral margin slightly convex, maximum width behind middle. Lateral margin crenulated, with nine to eleven small tubercles, with notch at each posterior angle. Base straight laterally, with obtuse keel at middle pointing posteriorly. Disc moderately convex in lateral view, with two raised paramedian carinae parallel to median line and diverging posteriorly, with median line narrow, long, and ending in pits anteriorly and posteriorly, with four additional shorter carinae bilaterally, the paralateral one joining with the paramedian carinae at base and forming tooth-like tubercle pointing posteriorly, extended anteriorly as less raised paralateral carina. With inner lateral carina short, not connected to long outer lateral carina. All carinae sub-crenulate. Lateral margin broadly wing-like bent up, with six large and deep transverse pits existing of partly connected circular pits, the frontal one partly situated directly at the frontal margin and separated from the second one by a striking ridge.

***Elytron*** (Fig. 51): Flattened in anterior half (lateral view), moderately convex in frontal view. Sub-elongate, margin slightly convex in anterior half up to pseudohumerus, moderately convex to apex, maximum width at middle, sub-tuberculate. Pseudohumerus rectangular, with tooth not projecting. Apex rounded, with small but acute tooth at suture. Disc with interneur six sub-tuberculate, interneur one with indistinct trace of carina at base, interneur three with indistinct carina in anterior two thirds, interneur two running towards apex as convex line, conspicuously raised, not reaching apex; interneur four running in parallel to interneur two, not reaching base, shortened at apex. Interneur five and six with two rows of serial pits. Interneur five nearly not visible due to transversally connected pits.

***Hind wings:*** Fully developed.

***Lower surface:*** Antennal channel of pronotum with surface isodiametrically reticulated. Pseudoepipleura densely covered with beige-grey pili, with two rows of transversally connected pits, lateral margin of elytron smooth. Metepisternum elongate, with broad longitudinal groove. Metasternum, abdominal sternites one to five with numerous irregularly situated larger and smaller pits, sternites four to six sulcate, with band of small punctures at middle. Sternum six hollowed out concavely, with small blunt longitudinal carina at middle.

***Legs:*** Profemora with dorsal surface nearly smooth. Protibia with moderately long and curved terminal spine, laterally bidentate and with two strong setae towards base, dorsally and ventrally with two carinae. First tarsomere distinctly elongated, nearly as long as tarsomeres two to five together.

***External sexual dimorphism:*** Not observed.

***Male genitalia*** (Fig. 67): Median lobe small, in dorsal view cracked in basal third and at middle, thickened and gently arcuate in apical half, in lateral view nearly straight, thickened in apical third, with nearly no pili, apex spatulate, in cross section with bilaterally flattened at apex. Oroficium half as long as median lobe. Endophallus with group of indistinct microtrichia, with cluster of few short small spines towards apex. Dorsal paramere slender, bisinuate apically, with hyaline apex, with apophyses distinctly asymmetric, ventral one indistinct, both parameres distorted.

***Female genitalia*** (Fig. 82): Coxostylus slender, somewhat broadened in basal half, nearly straight in apical third, directly at apex with indistinct hook, at end of basal third with one large and eight slender nematiform setae laterally.

***Variation:*** At the lateral margin of the pronotum the number of tubercles varies inter- and intra-individually from nine to eleven. In the holotype the basal margin of the head is more distinctly emarginated.

#### Distribution

**Figure 84.**
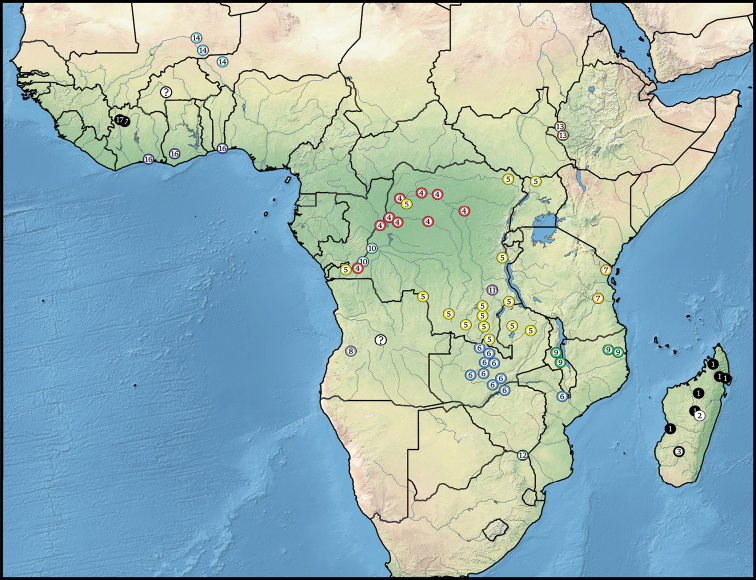
Map of tropical Africa providing an overview of the occurrence of all African *Salcedia* species described to date (recorded localities plotted). 1 (black *S. perrieri* Fairmaire; 2 (white) *S. unifoveata* sp. nov.; 3 (black circle) *S. faillei* sp. nov.; 4 (red) *S. coquilhati* Alluaud; 5 (yellow) *S. elongata* Alluaud; 6 (blue) *S. africana* (Britton); 7 (orange) *S. utetea* sp. nov.; 8 (grey) *S. procera* sp. nov.; 9 (light green) *S. robusta* sp. nov.; 10 (turquoise) *S. schoutedeni* Alluaud; 11 (purple) *S. lukulua* sp. nov.; 12 (dark green) *S. tuberculata* sp. nov.; 13 (brown) *S. baroensis* sp. nov.; 14 (light blue) *S. nigeriensis* Alluaud; 16 (dark blue) *S. putzeysi* (Oberthür); 17 (black) *S. matsumotoi* sp. nov.; ? in a circle (twice): collection locality of a single female *Salcedia* specimen of uncertain species. Basic schematic map of Africa taken from SimpleMappr.net.

(Fig. 84). Known from the type locality in Tharrawaddy, Burma (today Myanmar), and from Palon in Pegu (Myanmar). According to Andrewes (1929) another, fragmentary, specimen (“Ind. Mus.”) was collected at light in the Eden Gardens in Calcutta, Bengal, India. This specimen could not be located.

### Salcedia parallela

Taxon classificationAnimaliaColeopteraCarabidae

Baehr, 1998

6695FA9F-9C9A-5860-9C09-C160B0500964

Figs 25 , 43 , 83

Salcedia parallela Baehr, 1998: 2; Lorenz 2005: 155.

#### Type material.

**Holotype**: ♀, with labels and data: yellow, black printed and handwritten in black ink “BURMA (central) Mandalay, 20.9. 1984 D. Grohmann leg.” / red, black printed “HOLOTYPE *Salcedia parallela* sp. nov. det. M. Baehr 1997” (SMNS).

#### Remark.

The tarsomere of the right intermediate leg are missing.

#### Diagnosis.

A large sized species, with oblong-elongate outline of the elytra with maximum width at middle and the pronotum with three additional carinae of which the inner lateral carina is shortened to a rudiment and the outer one anteriorly shortened. The pseudohumerus is rectangular and with a distinct tooth. The antennomeres are elongate. Distinguished most clearly from the similar species *S. miranda* by the pronotum with the lateral margin straight and parallel and with eleven indistinct tubercles. Moreover, in *S. parallela* the interneur five and six of the elytron shows three rows of pits at middle.

#### Redescription.

Measurements in Table 1.

***Colour and surface:*** Anthracite grey with piceous traces, shiny; carinae of pronotum and elytra as well as margins of pronotum and elytra and surface of supra-antennal and supraorbital plates densely covered with beige-grey pili; legs, mandibles and scapus of antennae fuscous, other antennomeres lighter apically, palpi leoninous.

***Head:*** Five-sixths of width of pronotum. Outline semi-circular. Clypeus wide, straight anteriorly, fused with clypeal wings, separated from supra-antennal plates by indistinct obtuse notches and deep pit at each side, with raised oval-shaped field at middle, separated from frons by broad transverse furrow. Frons with two raised paramedian carinae, carinae convex, not joining anteriorly, with two small glossy teeth bilaterally to median carinae, with two shorter diverging carinae paralaterally at base; frons separated from supraorbital plates by deep broad furrows; with conspicuously broad pit at front-eye level; supra-antennal and supraorbital plates keel-like raised, supra-antennal plates vaulted. Base with angled emargination at middle (angle 130°), laterally obtuse angled (angle 102°). Eyes large, convex, genae higher as eyes, convex, both of them just visible from above, in lateral view shaped like a broad strip. Antenna with segments five to ten elongate (L/W 1.22), densely pubescent, segments two to four scarcely pubescent, scapus with longitudinal reticulation. Labrum straight anteriorly. Mandible moderately short, wide, slightly arcuate at apex. Mentum of moderate size, oval, epilobes wide, projecting, acutely angled anteriorly, with flattened margin at base, inner side and anteriorly, surface with longitudinal flat pits.

***Pronotum*** (Fig. 43): Outline rectangular, transverse, a third wider than long. Lateral margin almost straight, maximum width in middle third. Lateral margin crenulated, with eleven small tubercles larger basally, with notch at each posterior angle. Base straight laterally, with obtuse keel at middle pointing posteriorly. Disc nearly flat in lateral view, with two raised paramedian carinae parallel to median line and diverging posteriorly, with median line narrow, long, and ending in pits anteriorly and posteriorly, with four additional shorter carinae bilaterally, the paralateral one joining with the paramedian carinae at base and forming tooth-like tubercle pointing posteriorly and fringed with fine setae, extended anteriorly as less raised paralateral carina. With inner lateral carina very short, not connected to long outer lateral carina. All carinae sub-crenulate. Lateral margin broadly wing-like bent up, with six large and deep transverse pits existing of partly connected circular pits, the frontal one partly situated directly at the frontal margin and separated from the second one by a conspicuous ridge.

***Elytron:*** Flattened in anterior half (lateral view), moderately convex in frontal view. Oblong-elongate, margin straight in middle third, maximum width at middle, sub-tuberculate. Pseudohumerus rectangular (slightly over 90°), with distinct tooth. Apex rounded, with small but acute tooth at suture. Disc with interneur six sub-tuberculate, interneur three with indistinct longitudinal convexity at base, interneur two running towards apex as convex line, conspicuously raised, week apically, almost reaching apex; interneur four running in parallel to interneur two, almost reaching base, shortened at apex. Interneur five and six with three rows of serial pits. Pits of interneurs five and six partly connected transversally.

***Hind wings:*** Fully developed.

***Lower surface:*** Antennal channel of pronotum with surface irregular reticulated. Pseudoepipleura densely covered with grey pili, with partly doubled row of circular pits, lateral margin of elytron smooth. Metepisternum elongate, with broad longitudinal reticulated groove. Metasternum, abdominal sternites one to five with numerous irregularly situated smaller and larger pits, sternites four to six sulcate, four and five with band of small punctures at middle. Sternum six bilaterally slightly hollowed out concavely, with blunt longitudinal carina at middle.

***Legs:*** Profemora with dorsal surface irregularly reticulated. Protibia with moderately long and distinctly laterally curved terminal spine, laterally bidentate and with two strong setae towards base, dorsally and ventrally with two carinae. First tarsomere distinctly elongated, nearly as long as tarsomeres two to five together.

***External sexual dimorphism:*** Not observed.

***Male genitalia:*** Unknown.

***Female genitalia*** (Fig. 83): Coxostylus distinctly slender, obtusely and regularly curved, distinctly acute at apex, at end of basal third with one large and seven slender nematiform setae laterally, SSO with one fine pilus.

***Variation:*** Intra-individually, one side of the pronotum is straight and the other indistinctly convex.

#### Distribution

(Fig. 84). Known from the type locality in Mandalay, central Burma (today Myanmar).

## Discussion

### Taxonomy and relationships

A historical account is provided by Reichardt (1975) who summarised the three genera *Salcedia*, *Holoprizus* and *Solenogenys* in the tribe Salcediini, forming a very close but isolated group among the Scaritinae. Even after revising *Salcedia*, I do not see an African or Oriental group among the Civinini that the tribe Salcediini is more closely related to. Specific characters found in *Salcedia* can be found in Clivinini as well. For example, the campanulate outline of the head and the missing setae on the dorsal surface are found in *Sinesetosa* Balkenohl, 1996, the female coxostyli strongly remember to those of *Ancus*, the narrowed lateral channel of the elytron, especially at middle (enclosed by interneur seven and eight), with or without interruption of the umbilical setigerous punctures can be found in members of *Trilophus* Andrewes, *Triplophidius* Jeannel, *Syleter* Andrewes, 1941, and Clivina (subgenus Physioclivina Kult, 1959). However, these characters are seen at present as convergent development. Therefore, I share the opinion of Reichardt (1975) the group is isolated.

The monotypic genus *Androzelma* Dostal, 1993 keyed out among the Salcediini (Dostal 1993) does not, in my opinion, belong to the Salcediini but is clearly a member of the tribe Clivinini. Dostal himself considered its position in the Salcediini as uncertain (Dostal 1993: 120) and he listed the correct characters which place *Androzelma* into the Clivinini, an opinion also shared by Balkenohl (1996, 2001) and Baehr (1998). Moreover, and according to the description and figures, *Androzelma* possesses labial and maxillary palpi comparable to typical *Clivina* species. They are not small and cannot be retracted as is in Salcediini. The wax-like layer on the surface, which is evident in *Salcedia* and also to a lesser degree in *Solenogenys* and *Holoprizus* is absent in *Androzelma*. The short channel for the reception of the antennae in *Androzelma* is approximately as long as the eye, comparable with those of *Orictites* Andrewes, 1931 and *Sinesetosa* and is not prolonged up to the base of the head as it is in members of Salcediini. In my opinion, *Androzelma* belongs to the genus *Orictites* because it shares very many character states of that genus, which was recently revised (Balkenohl 2017a, b). However, this should be investigated in detail with the holotype at hand before a taxonomic change is performed.

### Zoogeography

At first glance, the collecting localities and the distribution pattern of the species look puzzling (Fig. 84). However, when looking in more detail at the African localities, a few trends can be outlined, although the distribution of the species is far from being well investigated.

The majority of the older material was collected in the Democratic Republic of the Congo. Obviously, more intensive collection took place there at the large river system of the Congo and its catchment area. At the middle and lower course of the River Congo three species occur, *Salcedia coquilhati*, *S. elongata*, and *S. schoutedeni*. *Salcedia elongata* seems to be the most widely distributed species. It populates the upper confluences of the Congo in the north-east up to the northern borders of Uganda, and in the south up to the watershed to the river system of the Zambesi in the north-west of Zambia. South of this watershed and at the Zambesi River, *S. africana* occurs. To the east, the East African rift valley with the Viphya Mountains obviously act as a barrier. East of the rift valley, the species *S. utetea* sp. nov. occurs in Tanzania and *S. robusta* sp. nov. in Mozambique.

The River Limpopo at the northern border of the Republic of South Africa is obviously populated by a different species, *S. tuberculata* sp. nov., and at the influx to the River Nile, the Baro River in the east of Ethiopia *S. baroensis* sp. nov. occurs. In West Africa, the river Niger is populated by *S. nigeriensis* but the influxes of the Volta River system are obviously populated by the different species, *S. matsumotoi* sp. nov., and at the lagoons in the south of West Africa *S. putzeysi* occurs.

This and recent finds of smaller and larger series of specimens from Zambia 2002, Guinea 2004, Mozambique 2012/13, and Ivory Coast 2018 lead to the assumption the genus might be much more widely radiated around inland waterways throughout tropical Africa and might possibly populate nearly all the larger river systems.

For the Oriental region, only five specimens have been found, from non-specific documented localities. It is possible the genus has not radiated as widely over the Oriental region, but it is also possible that the genus was simply not often found there until today, due to their camouflaged way of life. The species occur at or near the River Tharrawaddy and the specimen from Calcutta mentioned by Andrewes (1929) as being deposited in the “Ind. Mus” was not available for study.

### Biology

Knowledge about the way of life of this genus is poor, and if available at all it refers only to adult specimens. According to the labels and few hints in the literature, the following can be summarised, and some conclusions might be discussed.

For the 648 African specimens examined, documentation on the labels is available directly or as coordinates for 40% that they were collected at or very close to water bodies (banks of rivers or lakes). For an additional 31% it could be ascertained in detail that the collecting spot is situated close to a river or a lake (e.g., with the aid of Google Earth Pro). Specimens of *S. procera* sp. nov. were collected at Lac Calundo (Angola) by two methods, collecting by hand under stones (“récolté sous des pierres”) and “à la lumière”. This shows the species obviously lives under stones at shores but becomes active nocturnally. In case of *S. putzeysi*, localities are on a languet between the Ébrié-Lagoon and the sea (Addah, Côte de Ivoire) or close to but not at the sea (Porto-Novo, Benin). In nearly all of these cases there is forest nearby, e.g., gallery forests. So, there is evidence for occurrence near inland water bodies for around 70% of the material investigated. None of the finds were made directly on the sea shore. The substrate adhering on many specimens consist of fine sediment (mud or clay), but not sand (as it is for example in *Lophocoryza* Alluaud, 1941). Consequently, the hypothesis can be drawn that specimens occur on the banks of rivers and lakes, dominantly in woodland, and many of the species are riparian. According to Erwin (1979) they can be categorised as “Waterside” and “Forest Floor”. Burgeon (1935: 159) quotes a report Alluaud provided when sifting detritus on the bank of a river after flooding: “les *Salcedia* restent longtemps immobiles et dans cet état, ils sont impossibles à distinguer des menus fragments végétaux”. This citation agrees with the shape and appearance of the surface of the species and some of the morphological specifics. Head, pronotum and hind body closely and exactly attach together in resting position, protecting the intersegmental connections. The integument is in general covered with mud. This all gives the surface a homogenous look like a small half-rotten leaf. The labrum is widely covered by the clypeus and the palpi can be moved completely between the clypeus and the epilobes of the mentum as can be observed in several mounted specimens (compare examples in Fig. 6). The antennae are well sheltered in the channel of the head and pronotum. The lateral gap of the channel between the head and pronotum is closed by a tubercle of the pronotum furnished with a bunch of setae, and ventrally by the front legs with broadened femora. So, the sensitive parts of the body remain well protected and clean. In addition, there is a longitudinal groove ventro-laterally on the metepisternum where the terminal end and the tarsomeres of the intermediate tibia exactly fit in when the legs are in an angled resting position. The hind legs are evidently bent in parallel to the abdomen. It is assumed the specimens withdraw the legs close to the body and take this resting position for long periods of time until the environmental conditions are optimal again. The dirt adhering to the surface makes the specimens practically invisible. The specific morphological features including the dirt on the integument is considered as part of a protection mechanism and survival strategy on river banks and in gallery forests where inundations occur often. It is similar to the evident layers of other beetles living in the mud of rivers and lakes, e.g., *Georissus* Latreille, 1809 (Hydrophilidae). This information not only suggests a riparian way of life, but also the preference to sludge habitats of *Salcedia* species near water.

**Figure 85.**
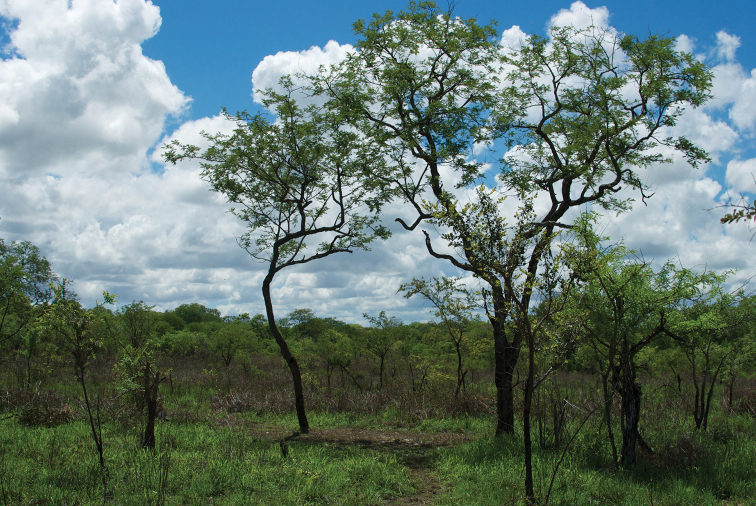
Habitat of *Salcedia robusta* sp. nov. in Mozambique, Cabo Delgado, Mareja P.N. Quirambas, with degraded Miombo woodlands. Photograph by François Génier, published with permission.

However, there is also a report of a collecting locality which is not directly located at a river or lake. According to François Génier, who collected abundant specimens of *Salcedia robusta* sp. nov. in Mozambique, “… there was no visible body of water near the trapping site (Fig. 85), but there was some heavy rain” (pers. comm.).

Forty-eight percent of the labels indicate collection at light. This matches with the observation of fully developed wings in 18 of the 20 species. It is astonishing what powerful fliers these small species are, because they have to carry relatively thick layers of dirt during flight, and the weight is greater when the dirt is wet. It is assumed the species are not able to fly high or for long periods of time. An exception is *S. unifoveata* sp. nov. from Madagascar, possibly representing a member of a different group within the genus. It was washed from the ground and does not possess the wax like adhesive film on the surface. However, the broader pits in this species are evidently filled with dirt. The hind wings of this species are reduced to a minute rudiment, and the eyes are reduced to a small reniform and concave bulge. It is assumed this species lives in a different type of microhabitat from the other species, possibly in deeper underground. In another species, *S. tuberculata* sp. nov., the hind wings are reduced by half. It is obviously brachypterous. The species was collected by “shorewashing” at an influx of the River Limpopo (Republic of South Africa). It is possible that the population of this species includes both brachypterous and macropterous specimens, which is also known in some other small Carabidae which inhabit river banks.

## Supplementary Material

XML Treatment for Salcedia

XML Treatment for Salcedia perrieri

XML Treatment for Salcedia unifoveata

XML Treatment for Salcedia faillei

XML Treatment for Salcedia coquilhati

XML Treatment for Salcedia elongata

XML Treatment for Salcedia africana

XML Treatment for Salcedia schoutedeni

XML Treatment for Salcedia nigeriensis

XML Treatment for Salcedia putzeysi

XML Treatment for Salcedia matsumotoi

XML Treatment for Salcedia procera

XML Treatment for Salcedia baroensis

XML Treatment for Salcedia utetea

XML Treatment for Salcedia lukulua

XML Treatment for Salcedia robusta

XML Treatment for Salcedia tuberculata

XML Treatment for Salcedia miranda

XML Treatment for Salcedia parallela
